# Supplementary material for: Parallel laboratory evolution and rational debugging reveal genomic plasticity to S. cerevisiae synthetic chromosome XIV defects
Source: Cell Genom. 2023 Nov 9;3(11):100379. doi: 10.1016/j.xgen.2023.100379 (PMC10667330; doi:10.1016/j.xgen.2023.100379)
Supplement: Document S1. Figures S1–S7 and Tables S2–S5 [file mmc1.pdf]

Cell Genomics, Volume *3*

Supplemental information

Parallel laboratory evolution and rational debugging reveal genomic plasticity to *S. cerevisiae* synthetic chromosome XIV defects

Thomas C. Williams, Heinrich Kroukamp, Xin Xu, Elizabeth L.I. Wightman, Briardo Llorente, Anthony R. Borneman, Alexander C. Carpenter, Niel Van Wyk, Felix Meier, Thomas R.V. Collier, Monica I. Espinosa, Elizabeth L. Daniel, Roy S.K. Walker, Yizhi Cai, Helena K.M. Nevalainen, Natalie C. Curach, Ira W. Deveson, Timothy R. Mercer, Daniel L. Johnson, Leslie A. Mitchell, Joel S. Bader, Giovanni Stracquadanio, Jef D. Boeke, Hugh D. Goold, Isak S. Pretorius, and Ian T. Paulsen

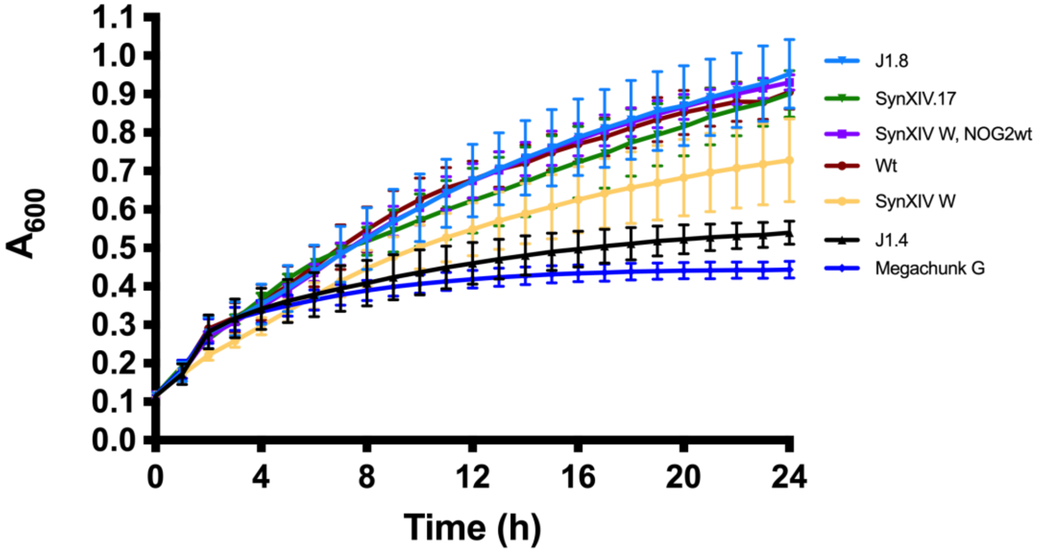

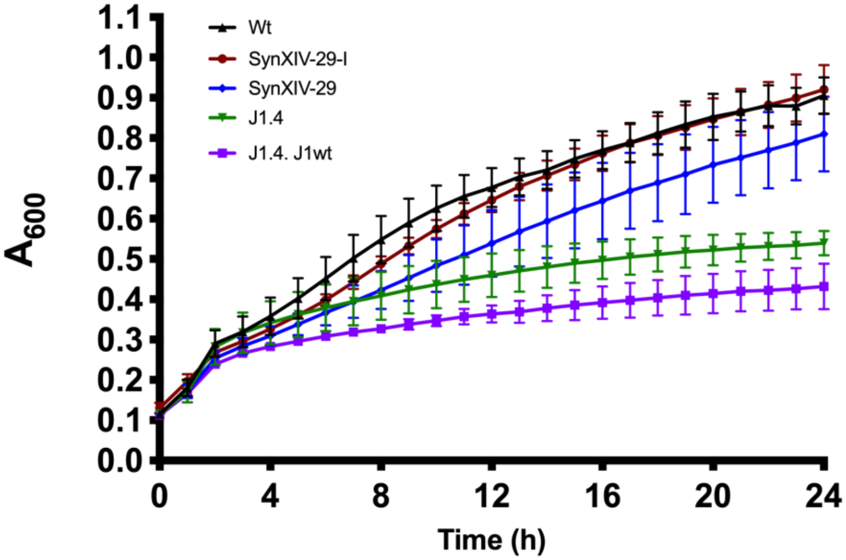

Figure S1. Fitness testing of major intermediate and defective strains made during synXIV construction

(**A**) Strains J1.8 (strain 40, Table S1), SynXIV.17 (strain 55, Table S1), SynXIV W, NOG2wt (strain 63, Table S1), Wt (strain 1 Table S1), SynXIV W (strain 57, Table S1), J1.4 (strain 39, Table S1), and megachunk G (strain 22, Table S1) were tested for fitness in liquid YPG medium. (**B**) Liquid medium fitness test of strains involved in the lead-up to the discovery of the *MRPL19* loxPsym defect (strains 37, 38, 39, 79 Table S1). Lines and error bars represent mean and ± 1 standard deviation of biological triplicate cultures. Related to Figure 1.

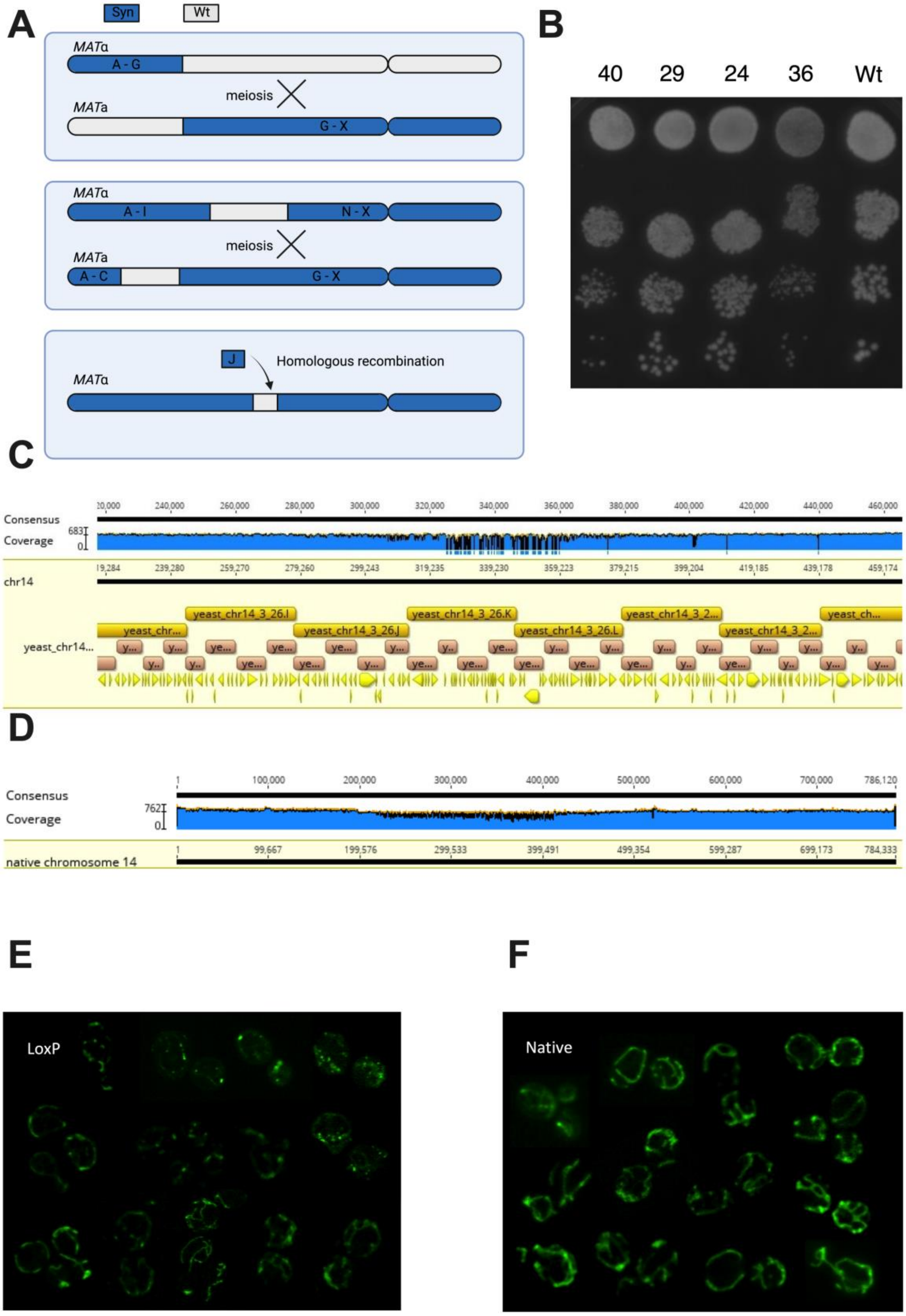

Figure S2. Pooled backcross sequencing and synthetic chromosome strain crossing reveals a fitness defect in megachunk J

(**A**) Megachunks A to X were integrated across two separate strains in parallel, with *URA3* and *MET17* markers integrated at the *YNL223W* and *YNR063W* wild-type loci of the synthetic A-G strain (strain 31, Table 3), respectively, prior to crossing with a synthetic G-X strain (strain 22, Table 3). Haploid strains resulting from the first cross (34 and 35, Table 3) were crossed together with screening for *LEU2*

(*YNL125C* locus) and *URA3* marker (*YNL269W* locus) loss from wild-type regions to give a fully synthetic version of chromosome XIV. (**B**) Haploid progeny of a meiotic cross between two partially synthetic versions (A-G and G-X) of synXIV (colonies 40, 29, 24, 36) were tested for fitness alongside a BY4741 control (Wt) on YPD agar at 30°C for 3 d. Colonies 40 and 36 contain fully synthetic chromosome XIV, while colonies 29 and 24 contain synthetic DNA in all megachunk regions except I-J and J respectively. (**C**) Pooled sequencing of 'fast' growing haploid progeny of a cross between synthetic chromosome XIV strain G-X and BY4742. Each set of reads were aligned to a reference S288c genome that also had a copy of the synthetic chromosome XIV sequence. (**D**) 'Slow' reads mapping to native chromosome XIV, with low-coverage regions equivalent to synXIV megachunk regions H to L. Dark yellow annotations correspond to megachunk regions while pink represent chunks, and yellow coding sequences. Average sequence coverage was 183 for the 'Fast' pool and 218 for the 'Slow'. Chromosome annotation and coverage graphs were generated using Geneious Pro Software. (**E**) Versions of the *MRPL19*-GFP fusion genes were designed with LoxP and (**F**) without LoxPsym motifs in the 3´ UTR GFP and expressed from pRS416 in the wild-type BY4741 strain. GFP fluorescence and distribution was visualised using an Olympus FV 1000 confocal laser-scanning microscope. Microscopy images were analysed using ImageJ (https://imagej.nih.gov/ij/index.html). Images shown are representative of cells in independent biological triplicate populations. Related to Figure 2.

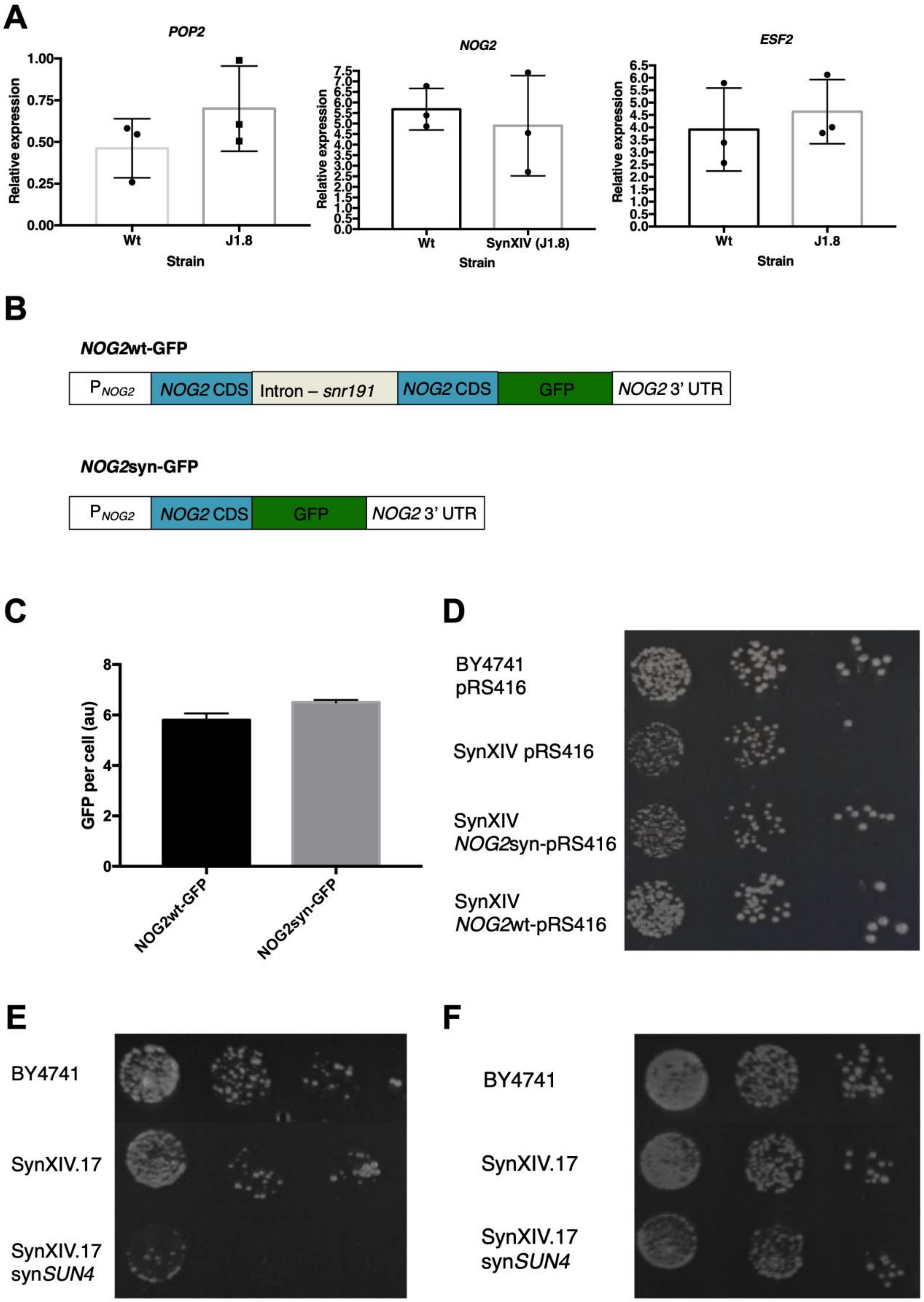

Figure S3. Expression of the *NOG2* and *SUN4* introns restores fitness

(**A**) mRNA levels of the three genes on chunk W1 were quantified using RT-qPCR. (**B**) Synthetic *NOG2-GFP* fusion expression constructs with and without the intron encoded *snr191* gene were designed and (**C**) tested for changes in protein expression in the wild-type (strain 1, Table 3). (**D**)

Expression of *NOG2-GFP* with its intron (strain 61, Table 3) but not without it (strain 62, Table 3) restores growth to wild-type levels in synXIV on YP-glycerol medium at 37°C. Images were taken after three days. RT-qPCR and GFP fluorescence levels are reported as the mean of triplicate cultures with error bars plus or minus one standard deviation. (**E**) Serial 10-fold dilutions of wild-type (BY4741), SynXIV (strain 63, Table 3), and SynXIV with the *SUN4* intron removed (strain 64, Table 3) on powdered yeast extract, and (F) on granulated yeast extract. Strains were plated on YP-glycerol (YPG) with non-granulated yeast extract (Merck 70161) at 37°C for 4 d prior to imaging. Related to Figure 4.

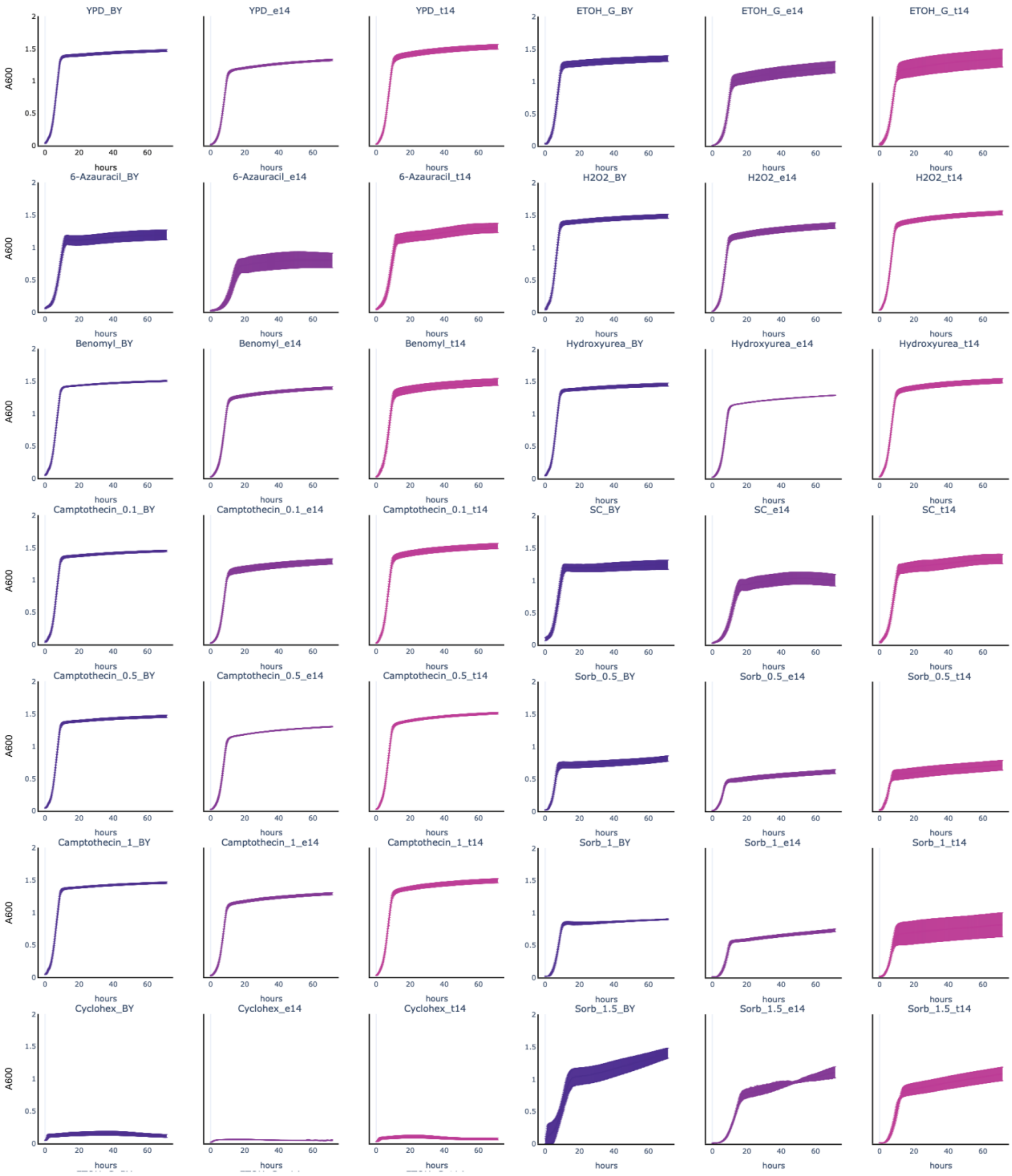

Figure S4. Fitness testing of final synXIV strain on different liquid media

Wild-type (BY4741 strain 75, Table S1), synXIV (strain 76, Table S1), and synXIV with tRNA genes reintroduced (strain 77, Table S1) were fitness tested in parallel on solid YPD, YPGE (1% Yeast extract 2% Peptone, 2% Glycerol, 2% Ethanol), or SC (Synthetic Complete) media with the indicated additives. YPD was used as the base medium for testing sorbitol, hydroxyurea, hydrogen peroxide, benomyl, 6-Azauracil, camptothecin, and cycloheximide. Lines and error bars represent mean and standard deviation from three biological replicates. Related to Figure 5.

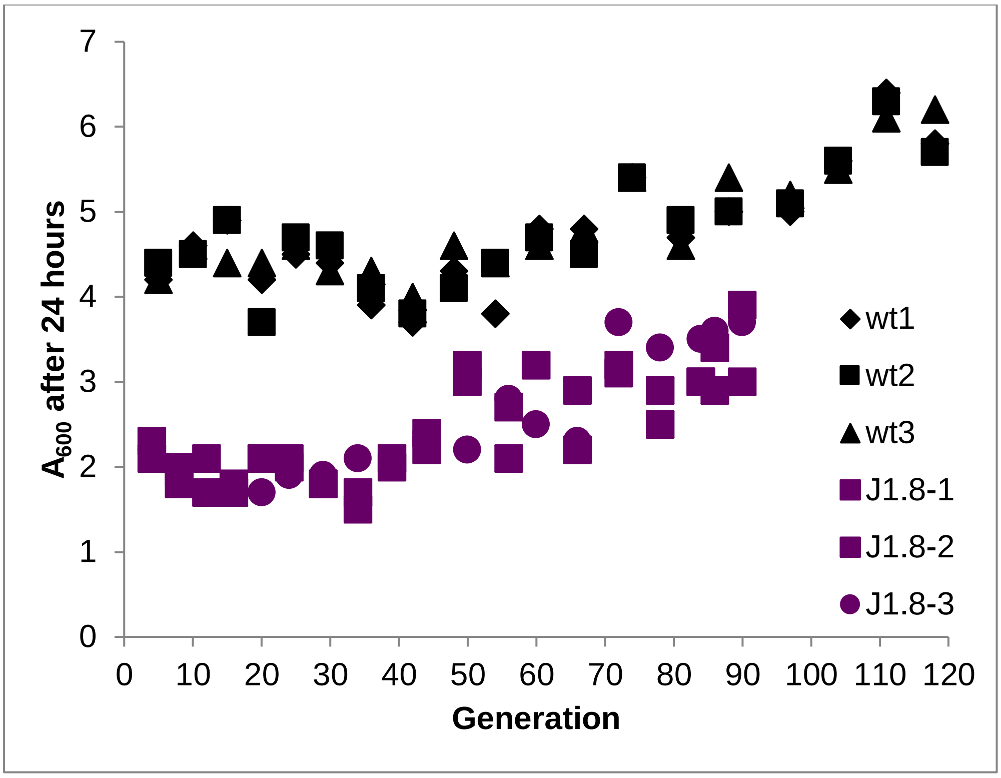

Figure S5. Fitness tracking of Adaptive Laboratory Evolution strains
A_{600} of three independent lineages for the BY4741 wild-type (strain 1 Table S1) and syn14 J1.8 strains (strain 40, Table S1) was measured every 24 h to track fitness and generation numbers in YP-glycerol medium. Related to Figure 6.

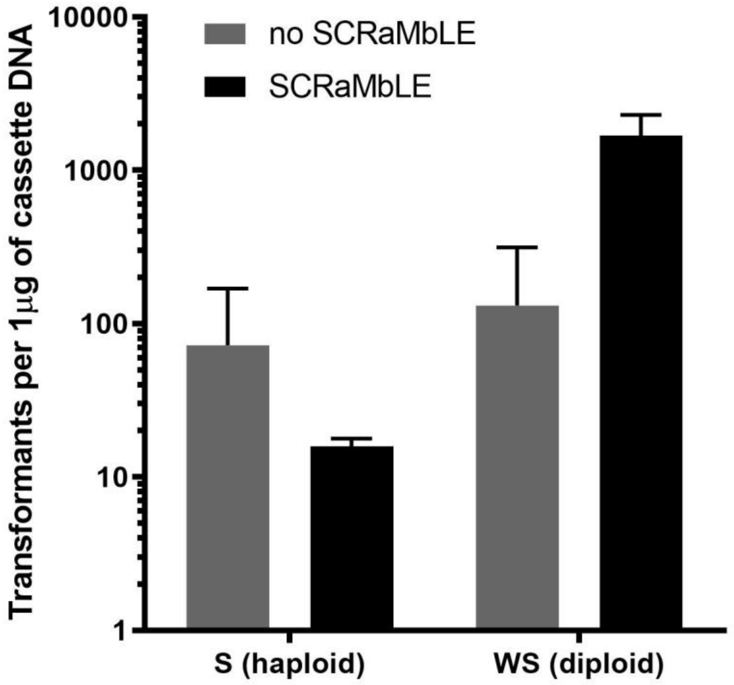

Figure S6. SCRaMbLE-mediated genome integration of a transformed *URA3* marker gene

Transformation efficiency in SCRaMbLE induced and uninduced haploid (strain 80, Table S4) and diploid strains (strain 86, Table S4). Both strains have the same number of synthetic chromosomes and, by extension, same number of available loxPsym integration sites. Related to Figure 7.

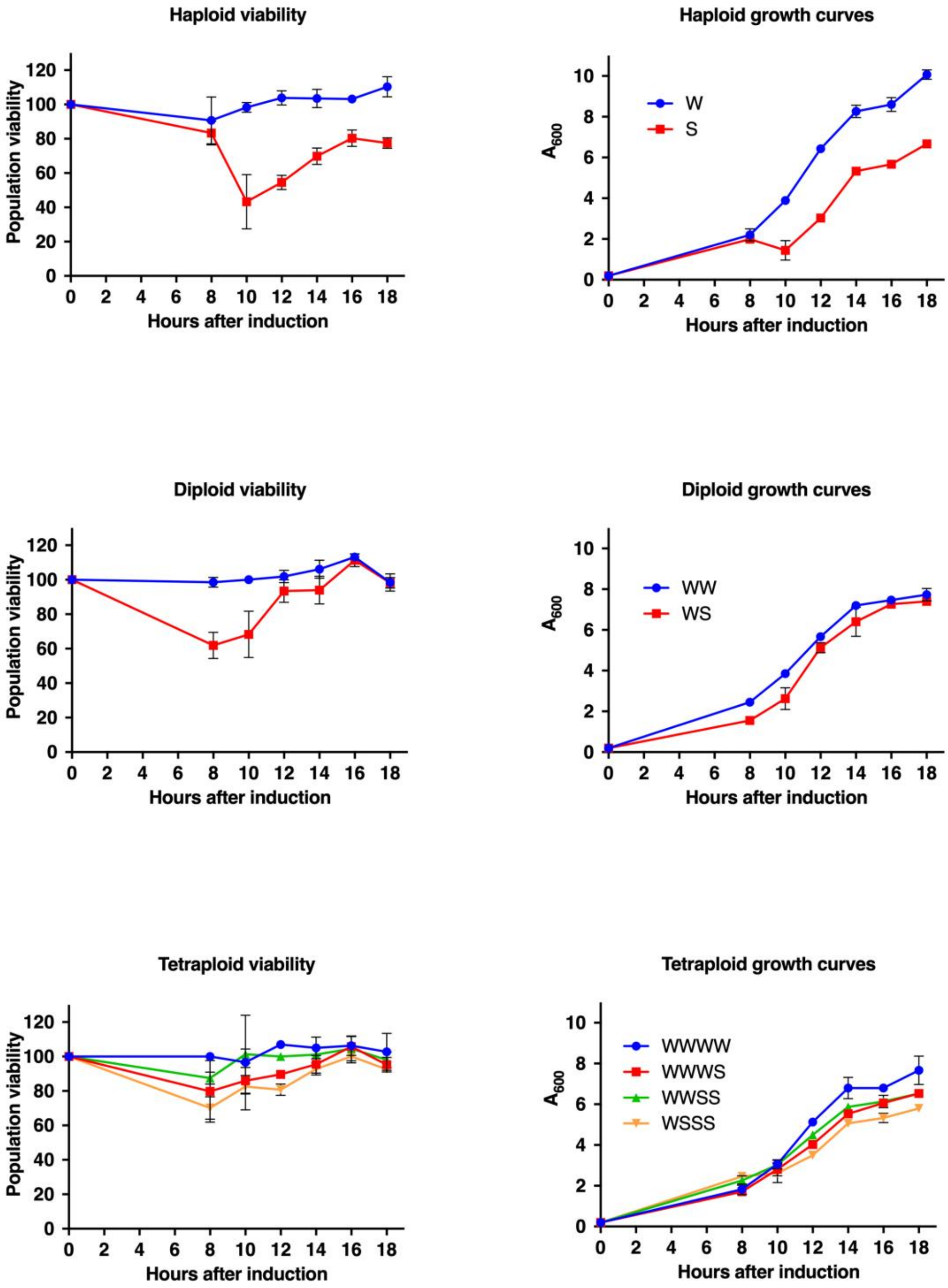

Figure S7. Growth and viability after SCRaMbLE of synthetic polyploid strains
The difference in cell density between SCRaMbLE'd and non-SCRaMbLE'd synthetic and wild-type cells of each strain were plotted for the haploid (A), diploid (C) and tetraploid (E) strains. The growth profiles of haploid (B), diploid (D) and tetraploid (F) strains in the presence of 1uM estradiol are shown. Data points and errors bars represent mean and standard deviation from three biological replicates. Related to Figure 7.

Table S2. Corrected sequence discrepancies in final synXIV strain, relates to STAR Methods.

Whole genome sequencing of synXIV strain revealed a number of missing Sc2.0 features and point mutations that deviated from the intended synXIV sequence. A subset of these were selected for repair according to their relative importance to the project, and ease of re-introduction. All missing TAA stop codons were repaired, as this modification will serve to free up the TAA codon to encode for non-natural amino acids in the future. All non-synonymous mutations in open reading frames were repaired to enable functional expression of the relevant proteins. Some missing loxPsym sites were corrected if they were nearby other features already being repaired, but were otherwise left as-is due to the fact that SCRaMbLE has a high degree of redundancy. PCR-tags are only used to verify the correct insertion of megachunks during the construction phase and were therefore left unaltered if missing, unless they were nearby another fix. Synonymous point mutations in open reading frames were also left unaltered.

Discrepancy Number	Discrepancy type	Original chunk/chromosome location	Gene(s)	Protein affect
1	T to C substitution	A3, 12735 bp	*YNL329C*	R276G
2	T insertion	A3, 13932 bp	*YNL328C*	Frame-shift
3	T to G substitution	A3, 13944 bp	*YNL328C*	N98H
4	C to T substitution	A3, 18438 bp	*YNL326C*	R302Q
5	G to T substitution	A3, 18512 bp	*YNL326C*	F277L
6	A to G substitution	A3, 18709 bp	*YNL326C*	Y212H
7	T insertion	A4, 19902 bp	*YNL325C*	Frame-shift
8	A to G substitution	A4, 22042 bp	*YNL325C*	synonymous
9	G to A transition	A4, 25505 bp	*intergenic*	-
10	G to A substitution	A4, 26370 bp	*YNL321W*	synonymous
11	Missing TAA stop codon	B4, 52811 bp	*YNL304W*	synonymous
12	Missing LoxP site	D4, 121257 bp	*YNL270C*	-
13	Missing TAA stop codon and LoxP site	E1, 125772 bp	*YNL268W*	synonymous

14	T to A substitution	E1, 125952 bp	*intergenic*	-
15	C to T substitution	E3, 110,759 bp	*YNL261W*	T377I
16	T to C substitution	K3, 336, 837 bp	*YNL149C*	None, TAA to TAG stop codon
17	Missing TAA stop codon	M3, 400807 bp	*YNL113C*	synonymous
18	Missing *Bsu36I* restriction site	M3-M4, 401355 bp	*YNL112W*	synonymous
19	T to C substitution	R1, 539,674 bp	*YNL037C*	T295A
20	Chunk V1 missing	V1, 2972 bp deletion beginning at 663,572 bp		YNR034 and YNR034W looped out between LoxP sites
21	Missing LoxP site	V4, 690721 bp	*YNR051C*	-
22	Missing TAA stop codon	V4, 690758 bp	*YNR051C*	synonymous
23	Missing PCR-tag	V4, 690776 bp	*YNR051C*	synonymous
24	Duplication on chromosome 12	*ECM22* to *HAP1*		

Table S3. Plasmids used in this study, relates to STAR Methods

Name	Details	Origin
pRS415	Yeast centromeric plasmid, *LEU2* marker	Euroscarf (Sikorski and Hieter 1989)
pRS416	Yeast centromeric plasmid, *URA3* marker	Euroscarf (Sikorski and Hieter 1989)
pRS413	Yeast centromeric plasmid, *HIS3* marker	Euroscarf (Sikorski and Hieter 1989)
TAR1-pRS413	*TAR1* expression from native promoter on the pRS416 plasmid	This study
pWAR1-crRNA-cas9-pRS423	*WAR1* promoter targeting guide RNA and Cas9 expression from the pRS423 plasmid. Template DNA for new crispr guide creation via PCR.	(Williams, Xu et al. 2017)
pPDR12-yEGFP-pRS416	*PDR12* promoter mediated expression of cytosol localised GFP. Positive ‘Free GFP’ control for confocal microscopy	(Williams, Xu et al. 2017)
MRPL19-GFP-MRPL19-LoxP-pRS416	MRPL19-GFP fusion protein expression construct with LoxPsym present in 3′ UTR	This study
MRPL19-GFP-MRPL19-Native-pRS416	*MRPL19*-GFP fusion protein expression construct with native 3′ UTR	This Study
NOG2wt-GFP-pRS416	Native *NOG2* promoter, intron, ORF, and terminator with in-frame ORF-yEGFP fusion	This study
NOG2syn-GFP-pRS416	Native *NOG2* promoter, no intron, ORF, and terminator with in-frame ORF-yEGFP fusion	This study
tRNA-pRS413		This study
pHK-Cre-EBDh	amp, CEN6/ARS4, SCW11p-CRE_EBD-ter, hphMX4 - For estradiol induced expression of Cre-recombinase. Contains a hygromycin resistance marker	This study
pLM006	amp, CEN6/ARS4, SCW11p-CRE_EBD-ter, HIS3 - For estradiol induced expression of Cre-recombinase. Contains a histidine auxotrophic marker	(Hochrein, Mitchell et al. 2018)

Table S4. Yeast strains used for polyploid construction, relates to Figure 7 and STAR Methods

Strain number	Strain name	Ploidy	Relevant genotype	Reference
79	BY4741(k)	n	*MAT*a, *his3Δ1, leu2Δ0, met15Δ0, ura3Δ0, mnn9::kanMX4*	Brachmann et al. (1998)
80	yLM896	n	*MAT*α, *leu2Δ0, MET15, his3Δ1, ura3Δ0;* synIII *HO::syn.SUP61;* SynIXL-synIXR; synVI *WT.PRE4*	Annaluru et al. (2014)
81	yLM896(L)	n	*MAT*α, *LEU2, MET15, his3Δ1, ura3Δ0;* synIII *HO::syn.SUP61;* SynIXL-synIXR; synVI *WT.PRE4*	This study
82	yLM896(H)	n	*MAT*α, *leu2Δ0, MET15, HIS3, ura3Δ0;* synIII *HO::syn.SUP61;* SynIXL-synIXR; synVI *WT.PRE4*	This study
83	BY4742(L)	n	*MAT*α, *his3Δ1, LEU2, lys2Δ0, ura3Δ0*	This study
84	BY4742(H)	n	*MAT*α, *HIS3, leu2Δ0, lys2Δ0, ura3Δ0*	This study
85	BY4742 x BY4741(k)<br><br>(WW)	2n	*MAT*α/a, *his3Δ1/his3Δ1, leu2Δ0/leu2Δ0, lys2Δ0/LYS2, MET15/met15, ura3Δ0/ura3Δ0, MNN9/mnn9::kanMX4*	This study
86	yLM896 x BY4741(k)<br><br>(WS)	2n	*MAT*α/a, *his3Δ1/his3Δ1, leu2Δ0/leu2Δ0, lys2Δ0/LYS2, MET15/met15, ura3Δ0/ura3Δ0, MNN9/mnn9::kanMX4;* chr III/synIII *HO::syn.SUP61;* chr IX/SynIXL-synIXR; chr VI/synVI *WT.PRE4*	This study
87	BY4742 x BY4741(k) x BY4742(L)<br><br>(WWW)	3n	*MAT*a/a/α, *his3Δ1/his3Δ1/his3Δ1, leu2Δ0/leu2Δ0/LEU2, lys2Δ0/LYS2/lys2Δ0, MET15/met15/MET15, ura3Δ0/ura3Δ0/ura3Δ0/, MNN9/mnn9::kanMX4/MNN9*	This study
88	BY4742 x BY4741(k) x yLM896(L)<br><br>(WWS)	3n	*MAT*a/a/α, *his3Δ1/his3Δ1/his3Δ1, leu2Δ0/leu2Δ0/LEU2, lys2Δ0/LYS2/lys2Δ0, MET15/met15/MET15, ura3Δ0/ura3Δ0/ura3Δ0/, MNN9/mnn9::kanMX4/MNN9;* chrIII/chrIII/synIII *HO::syn.SUP61;* chrIX/chrIX/SynIXL-synIXR; chrVI/chrVI/synVI *WT.PRE4*	This study
89	yLM896 x BY4741(k) x yLM896(L)	3n	*MAT*a/a/α, *his3Δ1/his3Δ1/his3Δ1, leu2Δ0/leu2Δ0/LEU2, lys2Δ0/LYS2/lys2Δ0,*	This study

	(WSS)		*MET15/met15/MET15, ura3Δ0/ura3Δ0/ura3Δ0/, MNN9/mnn9::kanMX4/MNN9;* chr III/synIII *HO::syn.SUP61//*synIII *HO::syn.SUP61;* chr IX/SynIXL-synIXR/SynIXL-synIXR; chr VI/synVI *WT.PRE4/*synVI *WT.PRE4*	
90	BY4742 x BY4741(k) x BY4742(L) x BY4742(H) (WWWW)	4n	*MAT*a/a/a/α, *his3Δ1/his3Δ1/his3Δ1/HIS3, leu2Δ0/leu2Δ0/LEU2/leu2Δ0, lys2Δ0/LYS2/lys2Δ0/lys2Δ0, MET15/met15/MET15/MET15, ura3Δ0/ura3Δ0/ura3Δ0/ura3Δ0, MNN9/mnn9::kanMX4/MNN9/MNN9*	This study
91	BY4742 x BY4741(k) x BY4742(L) x yLM896(H) (WWWS)	4n	*MAT*a/a/a/α, *his3Δ1/his3Δ1/his3Δ1/HIS3, leu2Δ0/leu2Δ0/LEU2/leu2Δ0, lys2Δ0/LYS2/lys2Δ0/LYS2, MET15/met15/MET15/MET15, ura3Δ0/ura3Δ0/ura3Δ0/ura3Δ0, MNN9/mnn9::kanMX4/MNN9/MNN9;* chrIII/chrIII/chrIII/synIII *HO::syn.SUP61;* chrIX/chrIX/chrIX/SynIXL-synIXR; chrVI/chrVI/chrVI/synVI *WT.PRE4*	This study
92	yLM896 x BY4741(k) x yLM896(L) x BY4742(H) (WWSS)	4n	*MAT*a/a/a/α, *his3Δ1/his3Δ1/his3Δ1/HIS3, leu2Δ0/leu2Δ0/LEU2/leu2Δ0, lys2Δ0/LYS2/lys2Δ0/lys2Δ0, MET15/met15/MET15/MET15, ura3Δ0/ura3Δ0/ura3Δ0/ura3Δ0, MNN9/mnn9::kanMX4/MNN9/MNN9; chrIII/*chr III/synIII *HO::syn.SUP61//*synIII *HO::syn.SUP61;* chrIX/SynIXL-synIXR/SynIXL-synIXR/chrIX; chrVI/synVI *WT.PRE4/*synVI *WT.PRE4/*chrVI	This study
93	yLM896 x BY4741(k) x yLM896(L) x yLM896(H) (WSSS)	4n	*MAT*a/a/a/α, *his3Δ1/his3Δ1/his3Δ1/HIS3, leu2Δ0/leu2Δ0/LEU2/leu2Δ0, lys2Δ0/LYS2/lys2Δ0/lys2Δ0, MET15/met15/MET15/MET15, ura3Δ0/ura3Δ0/ura3Δ0/ura3Δ0, MNN9/mnn9::kanMX4/MNN9/MNN9; chrIII/*synIII *HO::syn.SUP61/*synIII *HO::syn.SUP61//*synIII *HO::syn.SUP61;* chrIX/SynIXL-synIXR/SynIXL-synIXR/ SynIXL-synIXR/ chrVI/synVI *WT.PRE4/*synVI *WT.PRE4/*synVI *WT.PRE4*	This study

Table S5. Strain version table, relates to STAR Methods

Version name	Strain number	Comment	Details
yeast_chr14_0_00	NA	Wild-type chromosome 14 sequence	GenBank: BK006947.3
yeast_chr14_3_26	NA	Original design sequence	Final design by BioStudio
yeast_chr14_9_01	SynXIV 29 I J1.4, Strain 39, Table S1	synXIV Draft strain, with 5 TAG stop codons, 10 loxPsym sites missing, 23 wild-type, 4 point mutations causing amino acid changes, *YNL066W* intron present.	**Remaining TAG stop codons**: 125772 A->G, 336549 T->C, 400292 T->C, 400807 A->G, 690758 T->C. **Missing loxPsym sites**: 120565-120598, 125051-125084, 250019-250052, 337131-337164, 374188-374221, 438943-438976, 603336-603369, 690721-690754, 746919-746952, 747387-747420. **Wild-type PCRTags**: 108692-109106 YNL273W_525, 131105-131132 YNL264C_193,174923-174950 YNL243W_1380, 175389-175416 YNL243W_2607, 180372-180399 YNL242W_3522, 249297-249324 YNL201C_2362, 250194-250221 YNL200C_139, 250428-250455 YNL200C_373, 281076-281103 YNL183C_1189, 281403-281430 YNL183C_1516, 337177-337204 YNL148C_10, 337465-337492 YNL148C_298, 341073-341100 YNL144C_1021, 410750-410777 YNL106C_3133, 489097-489118 YNL066W_673, 491634-491661 YNL065W_609, 491925-491952 YNL065W_900, 495487-495514 YNL063W_360, 595657-595684 YNL005C_388, 658820-658847 YNR031C_4591, 689809-689836 YNR051C_19, 725470-725497 YNR065C_2761, 747793-747820 YNR073C_370, 748138-748165 YNR073C_715. **Point mutations that cause amino acid changes**: 12071 G->A, 140826 C->T, 396611 T->A, 450624 T->C.
yeast_chr14_9_02	SynXIV 29 I J1.8, Strain 40, Table S1	synXIV Draft strain, removed *MRPL19* LoxP site	**Remaining TAG stop codons**: 125772 A->G, 336549 T->C, 400292 T->C, 400807 A->G, 690758 T->C. **Missing loxPsym sites**: 120565-120598, 125051-125084, 250019-250052, 337131-337164, 374188-374221, 438943-438976, 603336-603369, 690721-690754, 746919-746952, 747387-747420, 278873-278906. **Wild-type PCRTags**: 108692-109106 YNL273W_525, 131105-131132 YNL264C_193,174923-174950 YNL243W_1380, 175389-175416 YNL243W_2607, 180372-180399 YNL242W_3522, 249297-249324 YNL201C_2362, 250194-250221 YNL200C_139, 250428-250455 YNL200C_373, 281076-281103 YNL183C_1189, 281403-281430 YNL183C_1516, 337177-337204 YNL148C_10, 337465-337492

			YNL148C_298, 341073-341100 YNL144C_1021, 410750-410777 YNL106C_3133, 489097-489118 YNL066W_673, 491634-491661 YNL065W_609, 491925-491952 YNL065W_900, 495487-495514 YNL063W_360, 595657-595684 YNL005C_388, 658820-658847 YNR031C_4591, 689809-689836 YNR051C_19, 725470-725497 YNR065C_2761, 747793-747820 YNR073C_370, 748138-748165 YNR073C_715. **Point mutations that cause amino acid changes**: 12071 G->A, 140826 C->T, 396611 T->A, 450624 T->C.
yeast_chr14_9_03	SynXIV.17c NOG2 wt, A2-A3, V4, V1, R1, *YNL114W*, Chr12, K3, *YNL116W*^{L697I}, E3. Strain 77, Table S1	SynXIV draft strain. 3 TAG stop codons changed to TAA, 4 LoxPsym sites added, 4 non-synonymous point mutations corrected, 3 PCR-tags added, and 18 PCR tags removed during reconstruction and repair. *NOG2* intron re-inserted,	**Remaining TAG stop codons**: 400292 T->C. **Missing loxPsym sites**: 125051-125084, 250019-250052, 278873-278906, 337131-337164, 374188-374221, 438943-438976, 603336-603369, 715110-715143. **Wild-type PCRTags**: 108692-109106 YNL273W_525,116368-116395 YNL271C_1528, 124292-124319 YNL268W_390, 124640-124667 YNL268W_738, 124700-124727 YNL268W_798, 124901-124928 YNL268W_999, 126914-126941 YNL267W_684, 127169-127196 YNL267W_939, 128195-128222 YNL267W_1965, 128627-128654 YNL267W_2397, 129047-129074 YNL267W_2817, 129242-129269 YNL267W_3012, 131105-131132 YNL264C_193,174923-174950 YNL243W_1380, 175389-175416 YNL243W_2607, 180372-180399 YNL242W_3522, 249297-249324 YNL201C_2362, 250194-250221 YNL200C_139, 250428-250455 YNL200C_373, 337177-337204 YNL148C_10, 337465-337492 YNL148C_298, 341073-341100 YNL144C_1021, 410750-410777 YNL106C_3133, 489097-489118 YNL066W_673, 491634-491661 YNL065W_609, 491925-491952 YNL065W_900, 495487-495514 YNL063W_360, 595657-595684 YNL005C_388, 658820-658847 YNR031C_4591, 691283-691310 YNR051C_484, 691337-691364 YNR051C_538, 691670-691697 YNR051C_871, 696935-696962 YNR054C_58, 697238-697265 YNR054C_361, 715318-715345 YNR061C_172, 715507-715534 YNR061C_361. **Point mutations that cause amino acid changes**: 450624 T->C.
yeast_chr14_9_04	SynXIV.17c NOG2 wt, A2-A3, V4, V1, R1, *YNL114W*, Chr12, K3, *YNL116W*^{L697I},	SynXIV final strain. Non-synonymous point mutation in *IDH1* reverted to wild-type sequence	**Remaining TAG stop codons**: 400292 T->C. **Missing loxPsym sites**: 125051-125084, 250019-250052, 278873-278906, 337131-337164, 374188-374221, 438943-438976, 603336-603369, 715110-715143. **Wild-type PCRTags**: 108692-109106 YNL273W_525,116368-116395 YNL271C_1528, 124292-124319 YNL268W_390, 124640-124667 YNL268W_738, 124700-124727 YNL268W_798, 124901-124928 YNL268W_999, 126914-126941 YNL267W_684, 127169-127196

	E3, R1. Strain 78, Table S1		YNL267W_939, 128195-128222 YNL267W_1965, 128627-128654 YNL267W_2397, 129047-129074 YNL267W_2817, 129242-129269 YNL267W_3012, 131105-131132 YNL264C_193,174923-174950 YNL243W_1380, 175389-175416 YNL243W_2607, 180372-180399 YNL242W_3522, 249297-249324 YNL201C_2362, 250194-250221 YNL200C_139, 250428-250455 YNL200C_373, , 337177-337204 YNL148C_10, 337465-337492 YNL148C_298, 341073-341100 YNL144C_1021, 410750-410777 YNL106C_3133, 489097-489118 YNL066W_673, 491634-491661 YNL065W_609, 491925-491952 YNL065W_900, 495487-495514 YNL063W_360, 595657-595684 YNL005C_388, 658820-658847 YNR031C_4591, 691283-691310 YNR051C_484, 691337-691364 YNR051C_538, 691670-691697 YNR051C_871, 696935-696962 YNR054C_58, 697238-697265 YNR054C_361, 715318-715345 YNR061C_172, 715507-715534 YNR061C_361.
