# Supplementary material for: Parallel laboratory evolution and rational debugging reveal genomic plasticity to S. cerevisiae synthetic chromosome XIV defects
Source: Cell Genom. 2023 Nov 9;3(11):100379. doi: 10.1016/j.xgen.2023.100379 (PMC10667330; doi:10.1016/j.xgen.2023.100379)
Supplement: Document S2. Article plus supplemental information [file mmc4.pdf]

Cell Genomics

Article

Parallel laboratory evolution and rational debugging reveal genomic plasticity to *S. cerevisiae* synthetic chromosome XIV defects

Graphical abstract

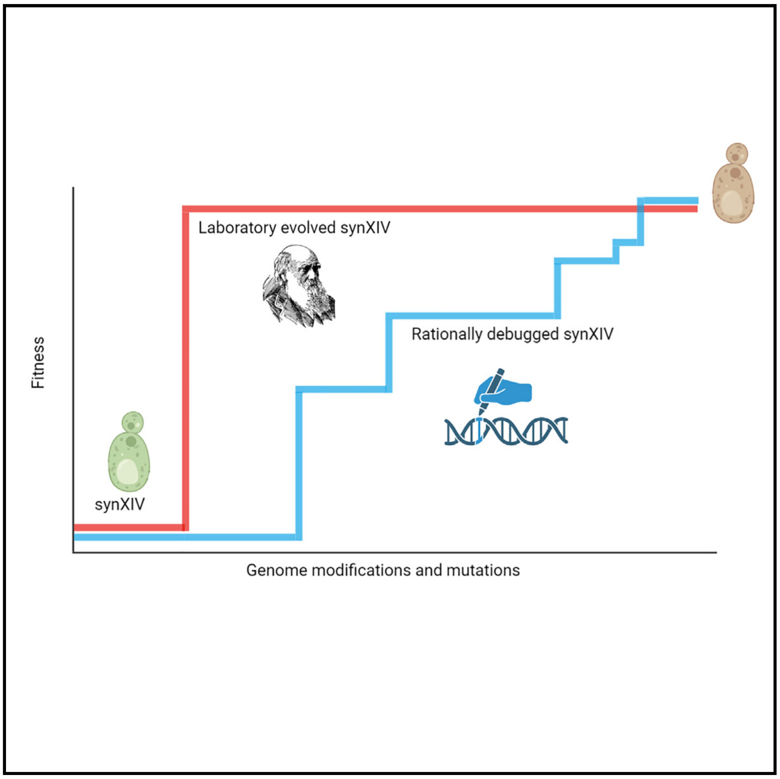

Highlights

- *Saccharomyces cerevisiae* synthetic chromosome XIV was synthesized and debugged
- Directed evolution and rational engineering were used in parallel to restore fitness
- Both approaches were successful but utilized different genotypes and mutations
- Hybrid synthetic-wild-type tetraploid strains improve post-SCRaMbLE viability

Authors

Thomas C. Williams, Heinrich Kroukamp, Xin Xu, ..., Hugh D. Goold, Isak S. Pretorius, Ian T. Paulsen

Correspondence

tom.williams@mq.edu.au (T.C.W.), sakkie.pretorius@mq.edu.au (I.S.P.), ian.paulsen@mq.edu.au (I.T.P.)

In brief

Williams et al. report on the design, construction, and debugging of *Saccharomyces cerevisiae* synthetic chromosome XIV (synXIV) as part of the international Sc2.0 project. SynXIV design changes led to severe growth defects, which were corrected through independent mechanisms discovered through parallel use of laboratory evolution and rational debugging techniques.

Williams et al., 2023, Cell Genomics *3*, 100379
November 8, 2023 © 2023 The Author(s).
https://doi.org/10.1016/j.xgen.2023.100379

CellPress

Cell Genomics

CellPress OPEN ACCESS

Article

Parallel laboratory evolution and rational debugging reveal genomic plasticity to *S. cerevisiae* synthetic chromosome XIV defects

Thomas C. Williams,[1,2,*] Heinrich Kroukamp,[1] Xin Xu,[1] Elizabeth L.I. Wightman,[1] Briardo Llorente,[1,2,3] Anthony R. Borneman,[4,5] Alexander C. Carpenter,[1,2] Niel Van Wyk,[1,6] Felix Meier,[1] Thomas R.V. Collier,[1] Monica I. Espinosa,[1] Elizabeth L. Daniel,[1] Roy S.K. Walker,[1,7,8] Yizhi Cai,[7,9] Helena K.M. Nevalainen,[1] Natalie C. Curach,[1,10] Ira W. Deveson,[11,12] Timothy R. Mercer,[11,12,13] Daniel L. Johnson,[1,4] Leslie A. Mitchell,[14] Joel S. Bader,[15] Giovanni Stracquadanio,[16] Jef D. Boeke,[14,17,18] Hugh D. Goold,[1,19] Isak S. Pretorius,[1,*] and Ian T. Paulsen[1,3,20,*]

[1]School of Natural Sciences, ARC Centre of Excellence in Synthetic Biology, Macquarie University, Sydney, NSW, Australia
[2]CSIRO Synthetic Biology Future Science Platform, Canberra, ACT 2601, Australia
[3]The Australian Genome Foundry, Sydney, NSW, Australia
[4]The Australian Wine Research Institute, Adelaide, SA 5064, Australia
[5]School of Agriculture, Food & Wine, Faculty of Sciences, University of Adelaide, Adelaide, SA 5005, Australia
[6]Department of Microbiology and Biochemistry, Hochschule Geisenheim University, Geisenheim, Germany
[7]School of Biological Sciences, University of Edinburgh, Edinburgh EH9 3BF, Scotland, UK
[8]School of Engineering, Institute for Bioengineering, The University of Edinburgh, Edinburgh EH9 3BF, Scotland, UK
[9]Manchester Institute of Biotechnology, University of Manchester, 131 Princess Street, Manchester M1 7DN, UK
[10]Bioplatforms Australia, Research Park Drive, Macquarie University, Macquarie Park, NSW 2109, Australia
[11]St Vincent's Clinical School, University of New South Wales, Sydney, NSW 2010, Australia
[12]The Kinghorn Centre for Clinical Genomics, Garvan Institute of Medical Research, Sydney, NSW 2010, Australia
[13]Australian Institute for Bioengineering and Nanotechnology, University of Queensland, Brisbane, QLD, Australia
[14]Institute for Systems Genetics, NYU Langone Health, New York, NY 10016, USA
[15]Department of Biomedical Engineering, Johns Hopkins University, 3400 North Charles Street, Baltimore, MD 21218, USA
[16]Institute of Quantitative Biology, Biochemistry, and Biotechnology, SynthSys, School of Biological Sciences, University of Edinburgh, Edinburgh EH9 3BF, UK
[17]Department of Biochemistry and Molecular Pharmacology, NYU Langone Health, New York, NY 10016, USA
[18]Department of Biomedical Engineering, NYU Tandon School of Engineering, Brooklyn, NY 11201, USA
[19]New South Wales Department of Primary Industries, Orange, NSW 2800, Australia
[20]Lead contact
*Correspondence: tom.williams@mq.edu.au (T.C.W.), sakkie.pretorius@mq.edu.au (I.S.P.), ian.paulsen@mq.edu.au (I.T.P.)
https://doi.org/10.1016/j.xgen.2023.100379

SUMMARY

Synthetic chromosome engineering is a complex process due to the need to identify and repair growth defects and deal with combinatorial gene essentiality when rearranging chromosomes. To alleviate these issues, we have demonstrated novel approaches for repairing and rearranging synthetic *Saccharomyces cerevisiae* genomes. We have designed, constructed, and restored wild-type fitness to a synthetic 753,096-bp version of *S. cerevisiae* chromosome XIV as part of the Synthetic Yeast Genome project. In parallel to the use of rational engineering approaches to restore wild-type fitness, we used adaptive laboratory evolution to generate a general growth-defect-suppressor rearrangement in the form of increased *TAR1* copy number. We also extended the utility of the synthetic chromosome recombination and modification by *loxP*sym-mediated evolution (SCRaMbLE) system by engineering synthetic-wild-type tetraploid hybrid strains that buffer against essential gene loss, highlighting the plasticity of the *S. cerevisiae* genome in the presence of rational and non-rational modifications.

INTRODUCTION

The field of synthetic genomics encompasses the design, construction, and characterization of whole genomes and chromosomes. This new approach to genomics provides several unique opportunities. For example, the ability to make global genetic changes that are too numerous to implement in a stepwise manner, the capacity to discover new biological phenomena through the classic "design-build-test-learn" cycle of synthetic biology, and the potential to design genomes that encode superior industrial phenotypes[1,2] are all enabled by synthetic genomics. The Synthetic Yeast Genome project (Sc2.0) exemplifies these new possibilities via genome streamlining (removal of transposons and non-essential introns),

This is an open access article under the CC BY license (http://creativecommons.org/licenses/by/4.0/).

genome "defragmentation/refactoring" via the relocation of all tRNA genes to a separate neochromosome, telomere standardization, and through the placement of heterologous *loxP*sym recombination motifs just after the stop codon of every non-essential gene.[3] The 12-Mb *Saccharomyces cerevisiae* genome consists of 16 chromosomes, built by an international consortium adhering to central design principles.[4] The Sc2.0 consortium has already completed six and one-half synthetic yeast chromosomes, resulting in new fundamental biological knowledge and genome construction technology. For example, novel growth-defect "debugging" (identifying and repairing deleterious design changes)[5] and chromosome consolidation[6] techniques have been developed, an in-depth phenotypic characterization of designed chromosomes[7,8] and the degree of genome plasticity with regard to ribosomal gene clusters,[9] and the effects of chromosome re-design on the three-dimensional genomic architecture have been investigated.[10]

The most significant design feature incorporated in Sc2.0 is an inducible evolution system termed synthetic chromosome recombination and modification by *loxP*sym-mediated evolution (SCRaMbLE). Induction of a heterologous Cre-recombinase enzyme results in inversions, duplications, translocations, and deletions of genes between *loxP*sym sites.[10–15] The induction of SCRaMbLE can in theory generate a virtually unlimited number of genomes with unique gene content and genomic architecture,[15] making it an extremely powerful tool for generating genetic diversity prior to laboratory evolution experiments and for understanding the genomic basis of selected phenotypes.[16,17] However, there are significant limitations to SCRaMbLE in its current form. For example, due to the relatively high incidence of gene deletions, there is a high frequency of lethal modifications in a SCRaMbLE'd population, significantly reducing genomic diversity. Specifically, potentially useful genomic rearrangements could be unobservable because they occur in a genome that also loses essential genes. This problem has been partially solved through the use of synthetic-wild-type heterozygous diploid strains, where the presence of non-SCRaMbLE-able chromosomes buffers against essential gene loss.[18,19] In addition to issues with lethality, SCRaMbLE is predominantly used to vary the gene content of synthetic yeast chromosomes, pathway-encoding linear DNA, or plasmids.[20] An ideal scenario would be for SCRaMbLE to give rise to the highest possible level of genetic variation without excessive cell death, and simultaneously enable the incorporation of multiple heterologous gene expression cassettes.

The main objective of this study was to re-design, construct, and debug *S. cerevisiae* synthetic chromosome XIV according to Sc2.0 principles. In addition to this core objective, we also explored the utility of adaptive laboratory evolution (ALE) to debug defects caused by chromosome re-design and expanded the utility of the Sc 2.0 SCRaMbLE system by introducing genomic redundancy via a series of novel hybrid tetraploid strains. Together, these parallel and complementary genome engineering techniques reveal the extreme plasticity of the *S. cerevisiae* genome to designed, semi-rational, and non-rational modifications.

RESULTS

SynXIV design and construction

S. cerevisiae synXIV was redesigned according to Sc2.0 principles using the BioStudio software package.[4] Briefly, 256 *loxP*sym sites were inserted 3 bp after the stop codons of non-essential genes, 14 introns were removed, open reading frames (ORFs) were synonymously recoded to contain a total of 1,040 PCR tags, 90 stop codons were swapped to TAA to free-up the TAG codon for potential future reassignment, native telomeres were replaced with standardized synthetic versions, and all transposon and tRNA sequences were removed. These changes resulted in a 753,097-bp synXIV divided into 24 "megachunks" labeled A–X, composed of four or five chunks labeled A1, A2, A3, A4, etc. (File S1), representing a 4% size reduction of the native 784,333-bp version. SynXIV was constructed according to the Sc2.0 Swap-In approach[4] across two different strains that were crossed to generate a near-complete version of synXIV (Figure S2A). Figure 1 shows an overview of the entire synXIV construction and debugging process. The initial strain was constructed via megachunk integration and multiple rounds of backcrossing to identify a defect caused by *loxP*sym insertion in the 3′ UTR of *MRPL19* (strain 39; Figure 1A) and from background mutations on other chromosomes (crossing of strains 2 and 40) to generate a near-complete version of synXIV (strain 56; Figure 1A). This strain was used to integrate missing megachunk segments and discover growth defects associated with *NOG2* intron removal (strain 57; Figure 1B). Finally, sequence discrepancies between the designed and obtained synXIV sequencing such as missing TAA stop codons, non-synonymous mutations, and some *loxP*sym sites were repaired, generating a fully synthetic version of *S. cerevisiae* chromosome XIV. The growth profile of each major strain and its defective derivatives in liquid medium is shown in Figure S1.

*LoxP*sym insertion in the 3′ UTR of *MRPL19* causes a respiratory growth defect

The synXIV strain with megachunks (i.e., ~30- to 60-kb synthetic DNA fragments) G–X showed a growth defect after the integration of the first megachunk, which was shared with all subsequent strains. However, the growth defect was not observed when megachunk G was re-integrated in a wild-type BY4741 strain. Whole-genome re-sequencing of the original megachunk G integrant strain revealed that chunks (i.e., ~10-kb synthetic DNA fragments) G1 and G2 had been integrated approximately four times, as indicated by coverage relative to surrounding chromosomal loci. When a descendant of this strain with megachunks G–O integrated was sequenced, the multiple copies were no longer present, and we conclude it had presumably been spontaneously looped out of the genome via homologous recombination. However, this strain and all subsequent megachunk integration strains shared a severe growth defect on YP-glycerol (YPG) medium. To ascertain the cause of this problem, backcrossing and pooled fast/slow strain sequencing was carried out as previously described.[5] A synthetic chromosome region spanning megachunks J–L was found to have low-coverage in "fast-grower" pool reads. Conversely, a wild-type chromosome region from megachunks H–L had low coverage in the

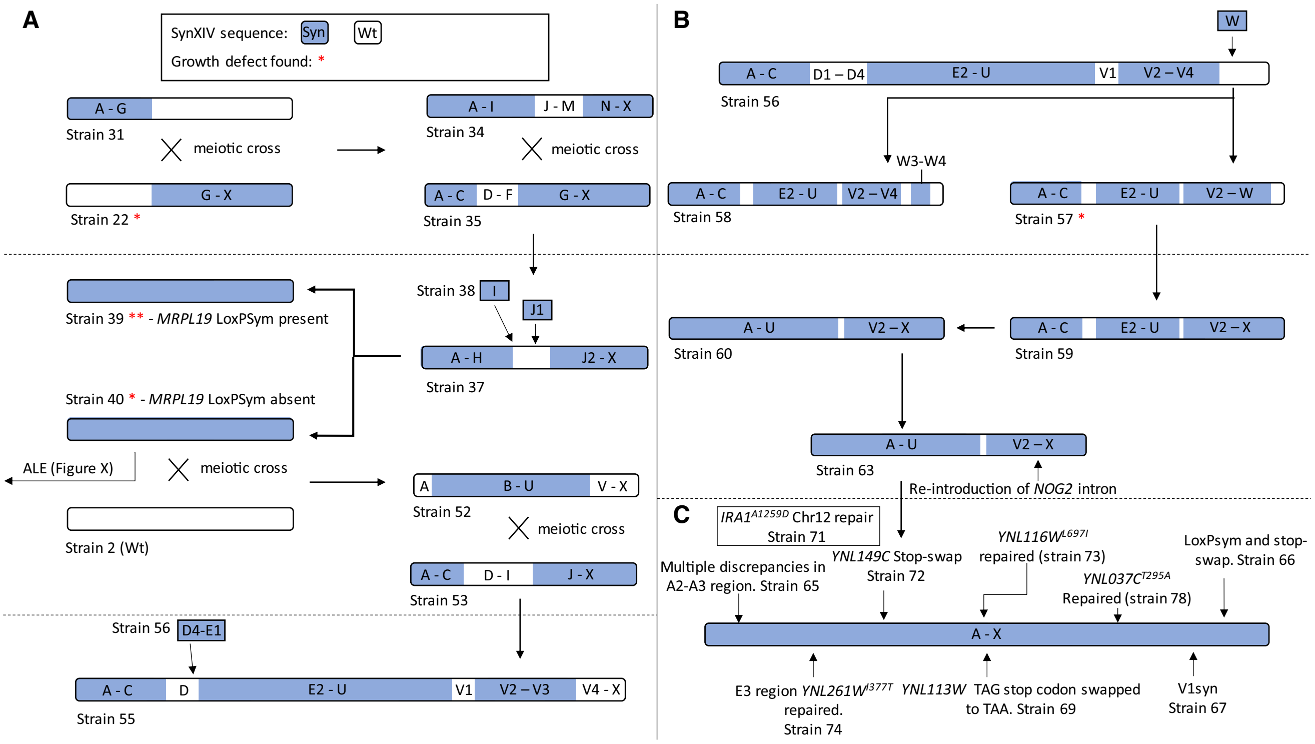

Figure 1. SynXIV construction and debugging overview

Semi-synthetic versions of *Saccharomyces cerevisiae* chromosome XIV with different synthetic megachunks present (blue) or absent (white).

(A) After initial parallel construction in two halves, semi-synthetic chromosome strains were progressively crossed with megachunk re-integration to generate a mostly synthetic strain free of growth defects (red asterisks).

(B) Strain 56 was used to re-integrate missing synthetic DNA and monitor for growth defects.

(C) Major discrepancies between the intended and obtained synXIV sequence were corrected to discover and correct final growth defects and generate a complete strain. Strain numbers correspond to Table S1.

pooled "slow-grower" reads (Figures S2C and S2D), suggesting that a synXIV modification within megachunks J–L led to the defect.

An independent line of inquiry also indicated that the megachunk J–L region was the cause of a major growth defect and further narrowed the location down to chunk J1. During the final meiotic cross of partially synthetic strains to produce a fully synthetic version of chromosome XIV (Figure S2B), two near-complete strains were identified that had wild-type I-J and J regions, respectively. These strains had improved fitness relative to two strains with fully synthetic versions of chromosome XIV on YPD at 30°C (Figure S2B), suggesting that the cause of a growth defect lay within the megachunk I-J region, independently supporting the backcrossing and pooled sequencing analysis. Integration of megachunk I in one of these faster-growing strains (SynXIV-29) did not cause any growth defect, indicating that the defect lay outside megachunk I (Figure S1B). When synthetic chunk J1 was then introduced, the severe growth defect on YPG was established (Figure 2A). Subsequent integration of the wild-type J1 region did not restore normal growth, initially leading us to dismiss this region as the cause of the growth defect (Figure S1B). During the integration of synthetic chunk J1, several strains were identified as having correct integration according to PCR-tag analysis, and one of these strains (J1.8) was found not to have the growth defect (Figure 2A). Whole-genome sequencing of slow- and fast-growing versions of the J1 integrants revealed that the fast-growing isolate J1.8 was missing a single *loxP*sym site immediately 3′ of the *MRPL19* gene (Figure 2B), whereas, in the slow-growing isolate (J1.4), this *loxP*sym was present. The *MRPL19* gene encodes a mitochondrial ribosomal protein, and deletion of this gene causes a respiratory growth defect.[21] Further analysis of the re-sequenced genomes showed that the slow-growing isolate had no reads mapping to the yeast mitochondrial reference genome, while the fast-growing isolate did. Loss of mitochondrial DNA is consistent with the fact that re-integration of wild-type chunk J1 did not restore growth (Figure S1B), as yeast cannot *de novo* regenerate the mitochondrial genome once it has been lost.[22] It is also consistent with the complete lack of respiratory growth on YPG seen from this defect (Figure 2A). YPG is commonly used in fitness testing since glycerol is a non-fermentable carbon source in *S. cerevisiae* and therefore leads to a slower growth rate and requires functional mitochondria.

We hypothesized that the presence of a *loxP*sym site in the 3′ UTR of *MRPL19* could modulate transcriptional termination efficiency or alter gene expression and hence performed RT-qPCR on RNA extracted from exponentially growing

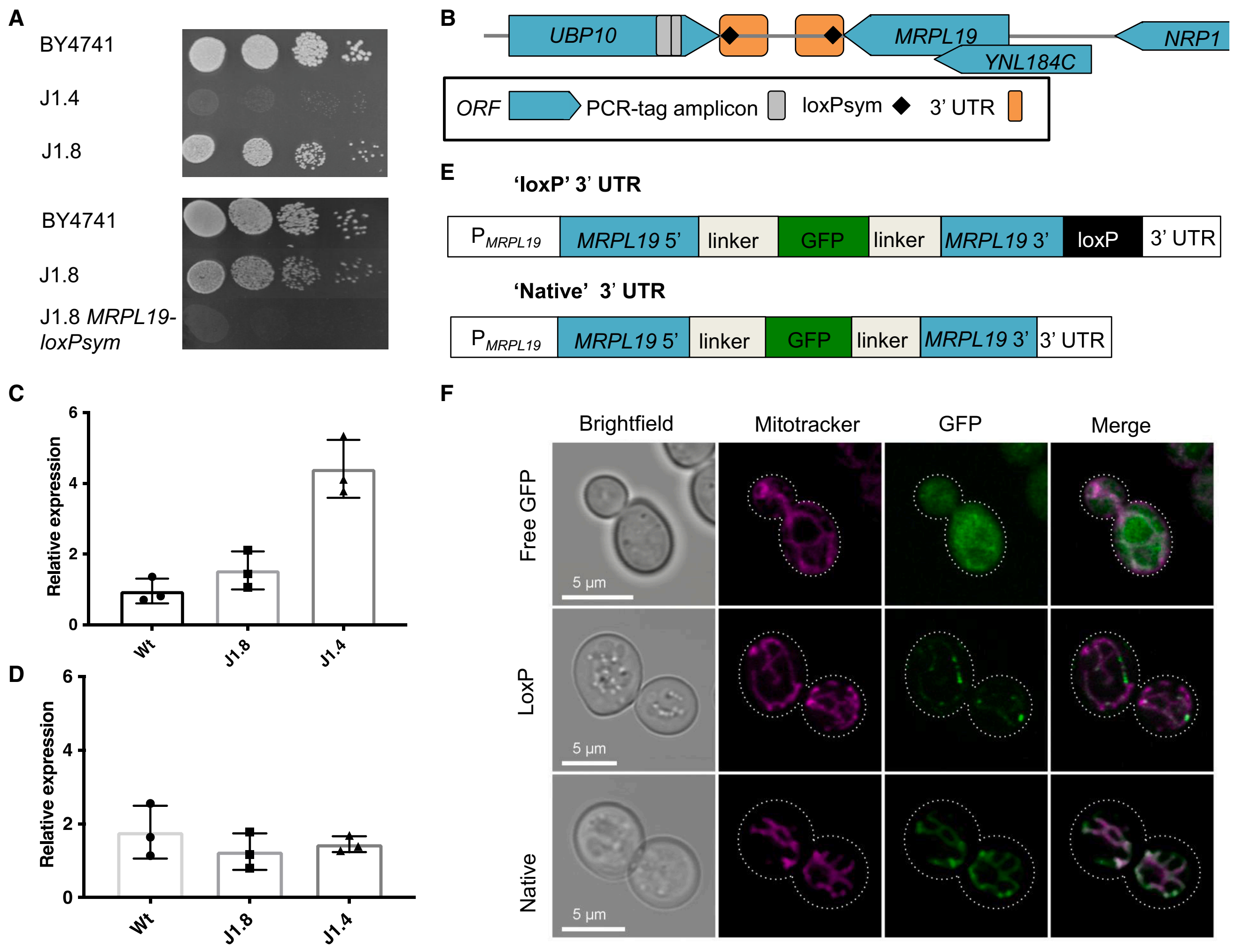

Figure 2. Chunk J1 growth defect and gene expression analysis

(A) YPG fitness test of chunk J1 integrants 4 and 8 (strains 39 and 40; Table S1) and the wild-type (BY4741, strain 1; Table S1) and wild-type, J1 integrant 8, and J1 integrant 8 with the *MRPL19 loxP*sym (strain 79; Table S1). Plates were incubated at 30°C for 3–4 days prior to imaging and are representative of two repeated experiments.

(B) Genetic context of the *MRPL19* gene and the surrounding synthetic chromosome design features.

(C and D) RT-qPCR of the *MRPL19* (C) and *NPR1* (D) genes was carried out on cDNA from BY4741 (wild-type), repaired synXIV (J1.8, strain 40; Table S1), and growth defect synXIV (J1.4, strain 39; Table S1) strains. Expression was normalized to the *ALG9* gene using the modified Livak method as previously described.[23] Bars and error bars represent mean and standard deviation from three biological replicates. Individual expression values of replicates are also shown.

(E) Two synthetic *MRPL19* promoter-gene-3′ UTR constructs were designed with a super-folder GFP encoded in the middle of the native ORF, separated by peptide linkers. One version contained a *loxP*sym motif 3 bp after the stop codon (termed *loxP*), while the second version contained no *loxP* within the native 3′ UTR (termed native).

(F) BY4741 strains expressing either of these two constructs (strains 49 and 48) or a cytosol-localized GFP (termed free GFP,[24] strain 50; Table S1) were grown in the presence of 100 nM Mitotracker Red (Thermo Fisher) to stain mitochondria. An Olympus FV 1000 confocal microscope was used to visualize yeast cells with bright field, MitoTracker, and GFP signals.

wild-type, J1.4, and J1.8 strains to test this. Interestingly, *MRPL19* transcript levels were significantly upregulated by approximately 5-fold in the slow-growing J1.4 isolate but were not significantly different between the wild-type and fast-growing J1.8 isolate (Figure 2C; 2-sided student t test, $p < 0.05$). The mRNA levels of the nearby *YNL184C* and *NPR1* ORFs were also measured to determine if the *MRPL19* 3′ UTR *loxP*sym site had any effects on their transcript levels. No mRNA was detected from the *YNL184C* ORF in any of the strains, while *NPR1* expression levels were not significantly different between the strains (Figure 2D). To test whether the observed upregulation of *MRPL19* mRNA could cause the growth defect in the slow-growing J1.4 strain, the native *MRPL19* gene and terminator were over-expressed from the strong-constitutive *TDH3* promoter[25] in the wild-type strain from the pRS413 vector. *MRPL19* over-expression did not cause a growth defect, suggesting the mechanism of the growth defect is unrelated to *MRPL19* over-expression.

CellPress
OPEN ACCESS

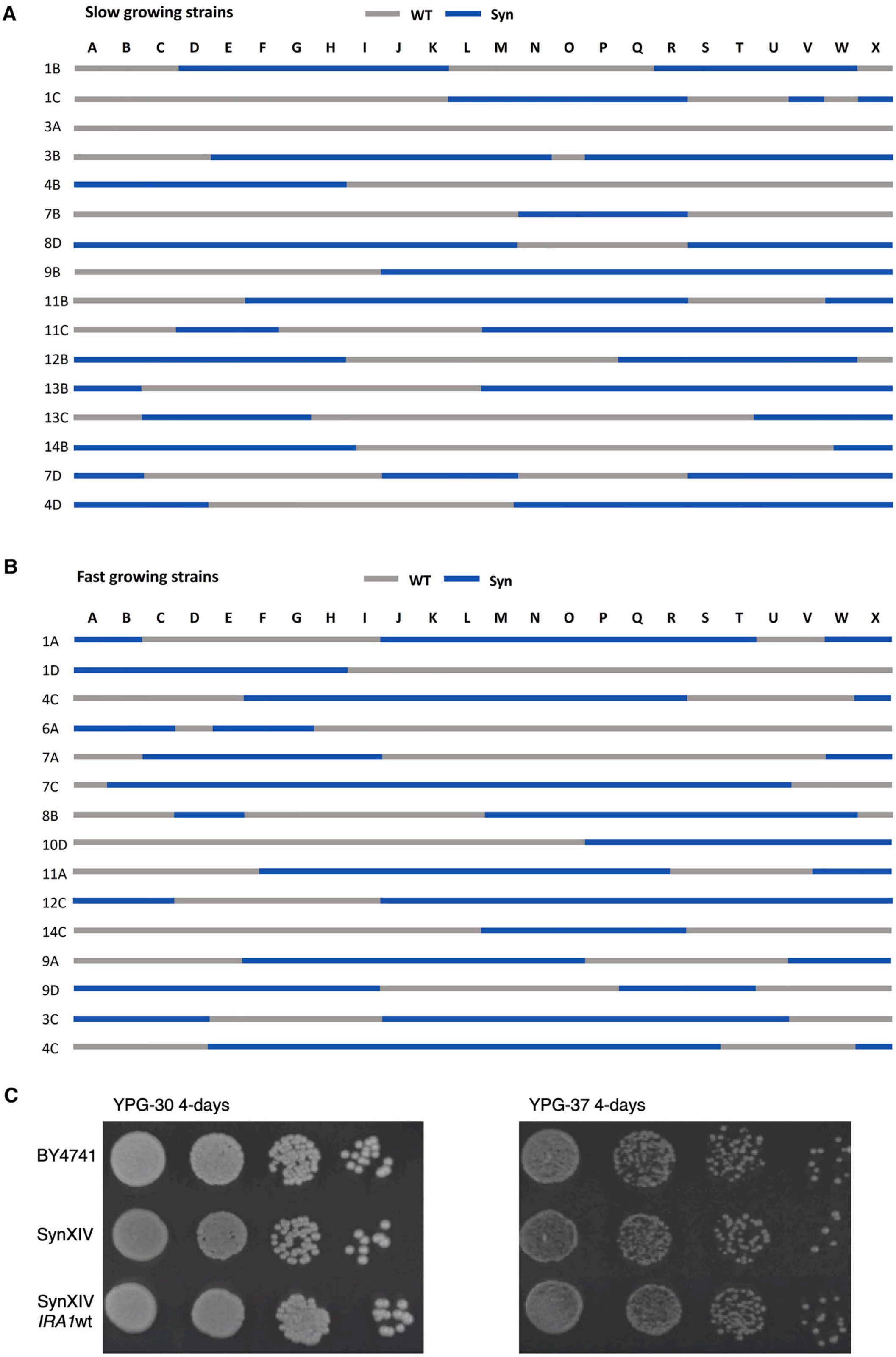

(legend on next page)

The *MRPL19* mRNA has a Puf3p recognition motif, and, when *PUF3* is deleted, there is no *MRPL19* mRNA localization to the mitochondria.[26] The addition of a *loxP*sym site to the 3′ UTR of *MRPL19* might therefore interfere with mitochondrial mRNA targeting, leading to the observed growth defect. To test this hypothesis, we designed a GFP fusion protein that retained the entire *MRPL19* coding sequence in order to account for the possibility that other RNA or protein signals are important for mitochondrial protein import (Figure 2E). Versions with the native 3′ UTR and with the *loxP*sym containing 3′ UTR were synthesized and analyzed for mitochondrial GFP signal in yeast (Figure 2F). Confocal microscopy of yeast cells with stained mitochondria (MitoTracker) showed that the cytosol-localized control ("free GFP") had no GFP signal correlation with the MitoTracker signal, while the native *MRPL19-GFP* construct ("native") resulted in uniform co-localization of GFP with the mitochondria (Figure 2F). In contrast, the insertion of a *loxP*sym motif in the 3′ UTR of *MRPL19* appeared to interfere with efficient mitochondrial delivery, as, although GFP and MitoTracker signals overlapped, the GFP signal was commonly reduced (Figures 2F, S2E, and S2F). Taken together with the observed growth defect, it is therefore likely that the *loxP*sym motif in the 3′ UTR of *MRPL19* interferes with correct mitochondrial delivery of this protein, leading to the observed growth defect under respiratory conditions (Figure 2A). This could involve either reduced mitochondrial targeting or import, resulting in the weak GFP mitochondrial signal.

SynXIV-wild-type backcrossing restores synXIV fitness

Although the J1.8 strain had near wild-type fitness, obvious defect(s) remained on solid YPG medium (Figure 2A). To investigate potential additional bugs in the synXIV J1.8 strain, it was backcrossed to the wild-type BY4742 strain. Haploid colonies resulting from individual randomly isolated spores were fitness tested and classified as fast or slow growers. Individual fast- and slow-growing spores were whole-genome sequenced, and the synthetic/wild-type complement of chromosome XIV was mapped in each case. In both the fast- and slow-growing spores, there were no synthetic or wild-type regions of chromosome XIV that clearly correlated with growth (Figures 3A and 3B). Additionally, there was one slow-growing haploid that had a completely wild-type version of chromosome XIV, and two fast-growing spores on YPG-37 medium that had almost complete versions of synXIV (12c and 7c). These observations suggested that there were mutations elsewhere in the genome that contributed to the slow-growth phenotype. Background mutations (outside of synXIV) that were not present in the original wild-type parental strain that were present in the backcrossed isolates included $MSH1^{P80A}$, $ATP1^{A424T}$, $ATP3^{I303V}$, $IRA1^{A1259D}$, $CMR1^{P87L}$, $DIA3^{Y273F}$, and $PDR5^{P496T}$ (File S2). This complement of genes and strains had two interesting features. First, there was an enrichment of genes associated with mitochondrial processes such as *ATP1*, *MSH1*, and *ATP3*. Second, 11 of 13 fast-growing isolates had the mutated *IRA1* gene, while 14 of 16 slow-growing isolates did not, suggesting this mutation might suppress defects encoded by either synXIV genes or other background mutations. Loci that were over-represented in the slow-growing isolates and under-represented in the fast-growing isolates included *PDR5* (69% compared to 31%) and megachunk W (69% compared to 38%). In order to remove deleterious background mutations and generate a synXIV strain with wild-type fitness, the fast-growing 12c and 7c (52 and 53; Table S1) strains were crossed and the resulting haploids screened for both fitness and synXIV completeness. One strain was identified (synXIV.17 strain 55; Table S1) that had both wild-type fitness and a near-complete synthetic chromosome XIV, with wild type DNA only present in megachunk D, W, and X regions. All background mutations were absent from synXIV.17 except $IRA1^{A1259D}$. Subsequent correction of the *IRA1* mutation on chromosome II during the final sequence discrepancy repair process (Figure 1C) with the wild-type sequence in this strain had no effect on fitness (Figure 3C, strain 71;; Table S1). This backcrossing process successfully generated a partially synthetic version of chromosome XIV in a genetic background free of deleterious mutations on other chromosomes. In addition to removing these putative deleterious background mutations, it was still possible that synthetic DNA in the megachunk D, W, and X regions could cause a growth defect, as these regions were wild-type in the fast-growing synXIV.17 isolate. We therefore re-integrated megachunks D, W, and X, in synXIV.17 while closely monitoring growth phenotypes.

Re-integration of megachunk W in the fast-growing near-complete synXIV.17 led to a fitness defect on YPG at 37°C (strain 57; Table S1), which was absent from a strain with only chunks W3 and W4 present (Figures 4A and 4B, strain 58; Table S1). The main Sc2.0 design change present on chunks W3 and W4 was the removal of the *NOG2* intron, which encodes a small nucleolar RNA (*snr191*) previously shown to cause a growth defect when deleted.[27] There were no differences in *NOG2* mRNA and protein expression with and without the *snr191* encoding intron present (Figures S3A–S3C), while reintroduction of the *NOG2* intron into the synXIV chromosome (Figure 4C) or via a plasmid (Figure S3D) restored fitness to wild-type levels. Functional expression of the *NOG2* intron was therefore important for growth independent of Nog2p and *NOG2* mRNA levels (Figure S3), and the intron was retained in synXIV. Similar to the *NOG2* intron, the *SUN4* intron located in the megachunk P region is also a "stable" intron that accumulates under stress conditions.[28] The *NOG2* and *SUN4* introns are the only known stable introns on chromosome XIV. Removal of the *SUN4* intron caused a minor growth defect in the complete synXIV strain (Figures S3E and S3F, strain 64; Table S1, strain version number yeast_chr14_9_01), leading us to retain the intron in the final design (yeast_chr14_9_04).

Figure 3. Synthetic DNA regions of chromosome XIV in haploid progeny of a synXIV-BY4741 meiotic cross

(A and B) Slow- (A) and fast-growing (B) strains were tested for their synthetic DNA content using PCR tag analysis. Megachunk regions A–X of chromosome XIV are displayed for each strain, with blue representing synthetic DNA and white wild-type DNA. All strains are haploid derivates of a cross between strains 1 and 40 (Table S1).

(C) Serial 10-fold dilutions of wild-type (BY4741), synXIV (strain 70; Table S1), and SynXIV with the $IRA1^{A1259D}$ mutation reverted to wild type (strain 71; Table S1). Each strain was plated on YP glycerol (YPG) at 30°C and 37°C for 4 days prior to imaging.

CellPress
OPEN ACCESS

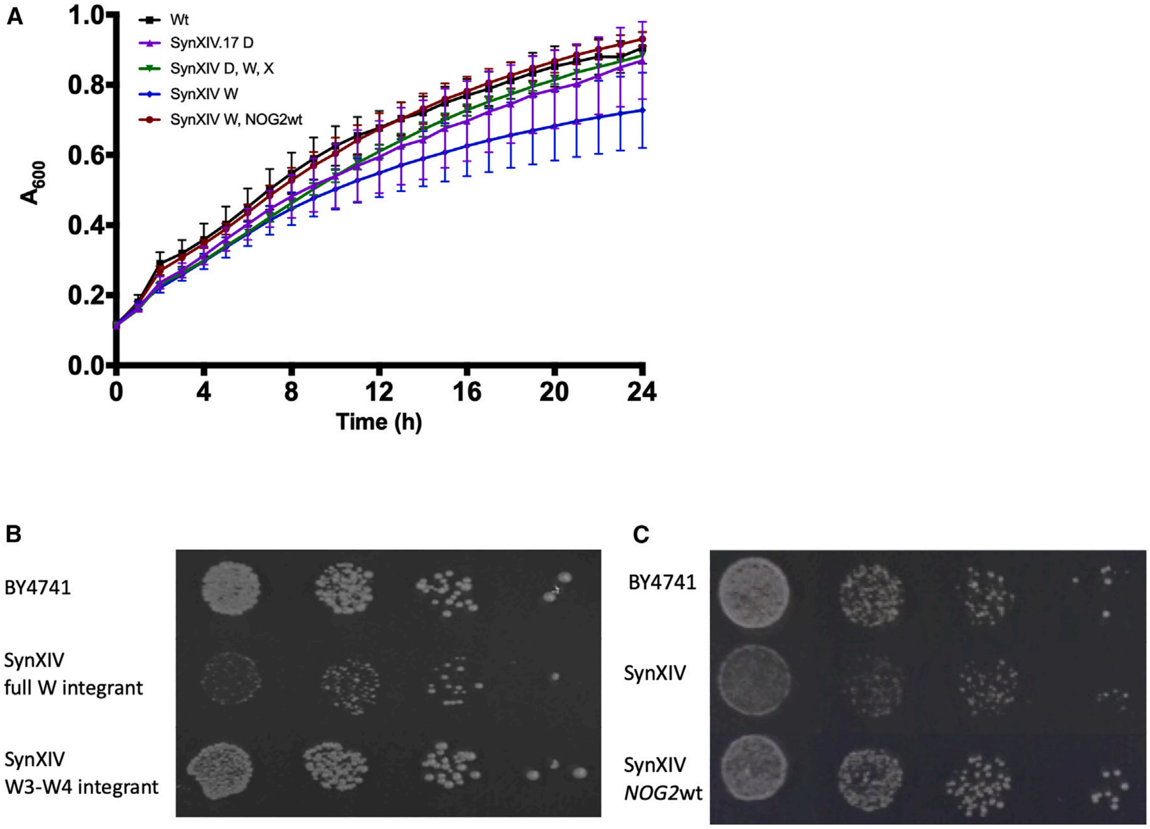

Figure 4. ***NOG2*** **intron growth-defect analysis**

(A) Growth in liquid YPG medium of synXIV (strain 56; Table S1) strains integrated with megachunks W, X, and with wild-type *NOG2* (strains 57, 59, and 63; Table S1). Lines and error bars represent mean and ±1 standard deviation of A_{600} from biological triplicates.

(B) Integration of megachunk W (strain 57; Table S1) but not chunks W3-W4 (strain 58; Table S1) causes a growth defect in SynXIV.17.

(C) Re-insertion of the *NOG2* intron in the synXIV.17c strain restores wild-type fitness (strain 63; Table S1). Spot assays are 10-fold serial dilutions of exponentially growing cultures on YPG medium at 37°C. Images were taken after 5 days (B) and 3 days (C).

Sequence discrepancy repair

Sequences that deviated from the intended synXIV sequence were introduced during the construction and debugging of synXIV, with important features repaired to make the final strain (Table S2). While features such as missing PCR tags, restriction enzyme ligation sites, and absence of a small number of *loxP*sym motifs is not critical for the intended function of the synthetic yeast genome, other features, such as stop-codon reassignment in verified ORFs, are expected to be critical in the event of future reassignment of the TAG codon. Additionally, it was possible that non-synonymous mutations in coding sequences could contribute to unidentified phenotypes. All erroneously remaining TAG stop codons were therefore swapped to TAA (except the stop codon of the dubious ORF *YNL114C*), and non-synonymous coding sequences were repaired to produce the final synXIV strain (Table S2). Non-critical discrepancies listed in Table S2 were repaired due to their proximity to essential discrepancy repairs.

Fitness testing of final synXIV strain with and without tRNA complementation

An important design parameter of the Sc 2.0 project is to maintain as close to wild-type fitness as possible in synthetic chromosome strains. Having healthy single synthetic chromosome strains is expected to make the process of combining individual synthetic chromosome strains more feasible, as well as improve the subsequent utility of the strain for fundamental and applied research. We therefore tested our final synXIV strain with (strain 77; Table S1) and without (strain 76; Table S1) complementation of the deleted tRNA genes on a pRS413 plasmid on multiple different media types alongside the BY4741 wild-type strain (Figures 5 and S4). The synXIV strain had wild-type fitness in each of the solid media conditions tested, with no obvious effect from the presence of the tRNA plasmid (Figure 5). Interestingly, when the same tests were performed in liquid media (Figure S4), slight fitness defects were seen in the synXIV strain in media containing 6-azauracil, MMS (methyl-methanesulfonate), and 1.5 mM sorbitol, with these defects abrogated by the presence of the tRNA plasmid in 6-azauracil and MMS, but not in 1.5 mM sorbitol. These tests showcase the high level of fitness achieved with our synXIV strain and its appropriateness to move forward in the chromosome consolidation process. They also demonstrate the functional expression and utility of synthetic tRNA genes under stressful conditions.

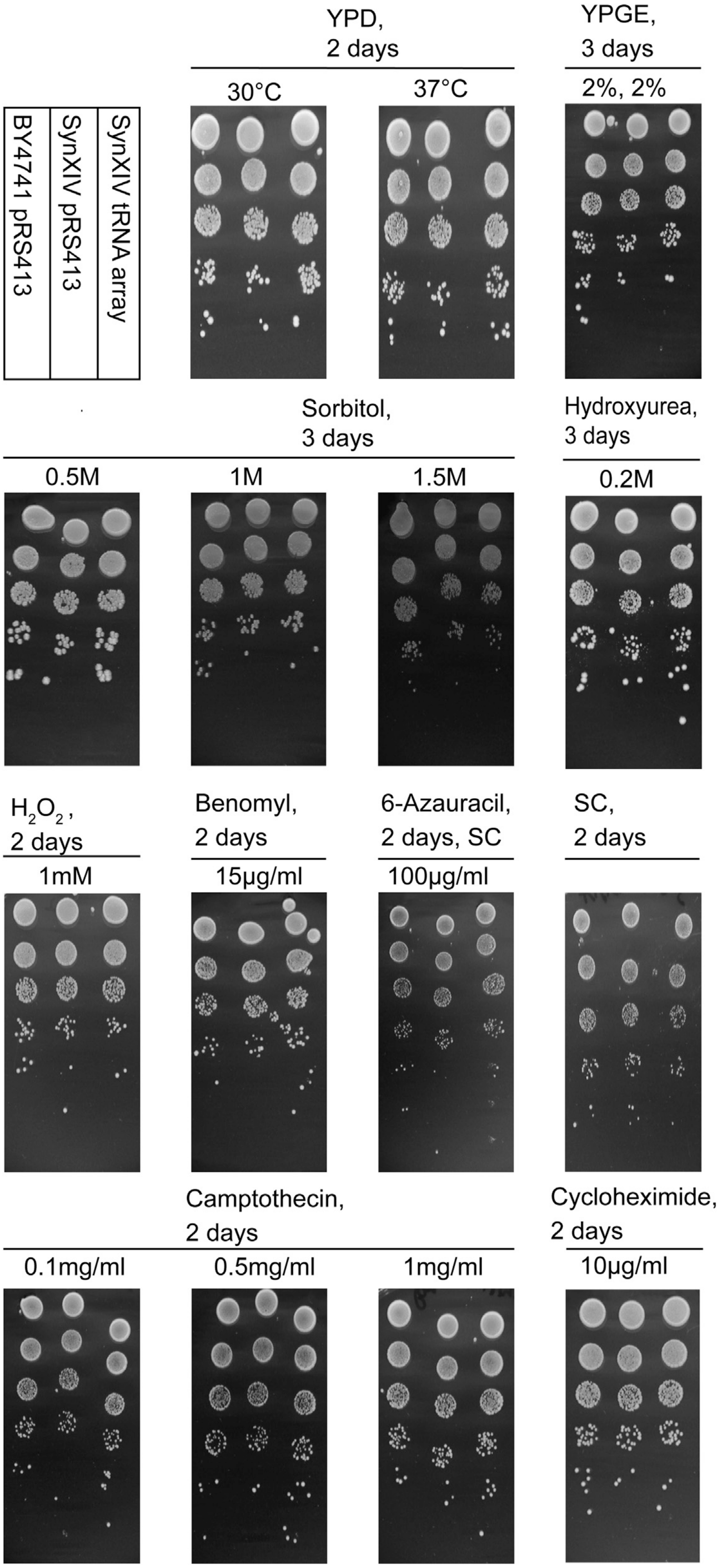

Figure 5. Fitness testing of final synXIV strain on different media

Wild-type (BY4741 strain 75; Table S1), synXIV (strain 76; Table S1), and synXIV with tRNA genes reintroduced (strain 77; Table S1) were fitness tested in parallel on solid YPD, YPGE (1% yeast extract, 2% peptone, 2% glycerol, 2% ethanol), or synthetic complete (SC) medium with the indicated additives. YPD was used as the base medium for testing sorbitol, hydroxyurea, hydrogen peroxide, benomyl, 6-azauracil, camptothecin, and cycloheximide. Strains were precultured twice in liquid YPD medium prior to spotting of 10-fold serial dilutions in 3-μL aliquots on agar plates, with images taken after the indicated number of days' incubation. All plates without temperatures indicated were incubated at 30°C. Images are representative of two repeated experiments.

CellPress
OPEN ACCESS

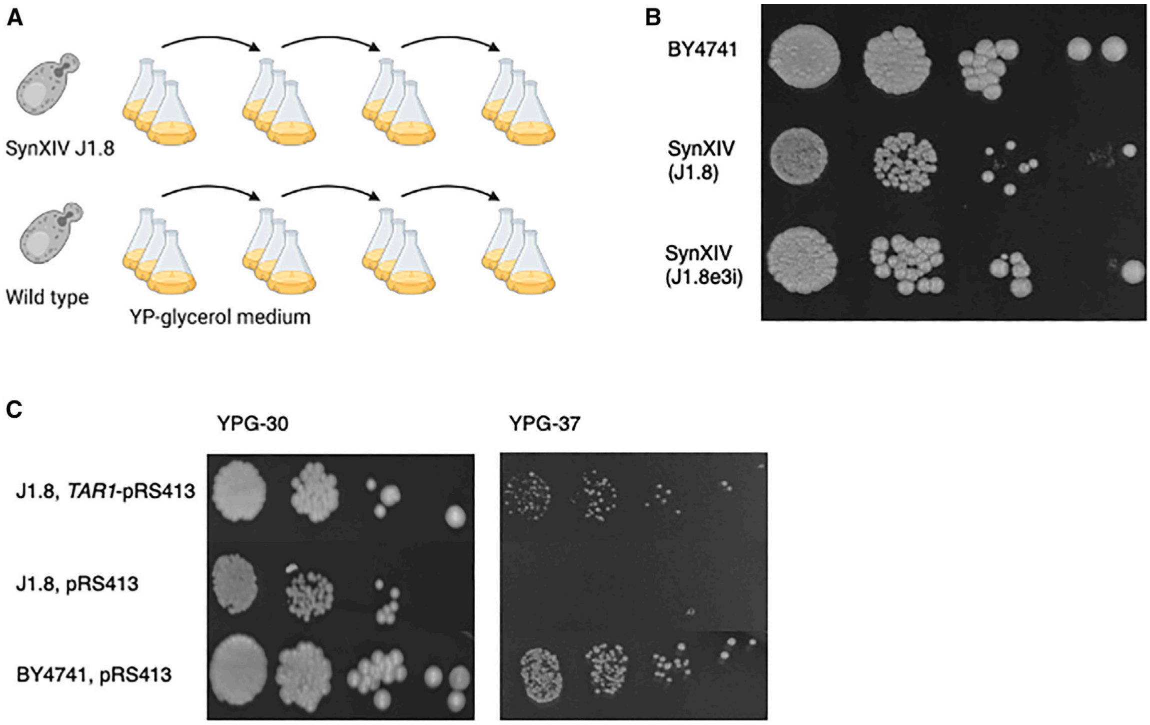

Figure 6. ALE of synXIV and wild-type strains on YPG medium

(A) BY4741 (wild-type, strain 1; Table S1) and synXIV strains (J1.8, strain 40; Table S1) were grown in YPG medium with passaging to fresh medium every 24 h.
(B) At the end of the evolution experiment, the fitness of the parental wild-type and J1.8 strains was compared with one of the evolved J1.8 lineages (J1.8e3i, strain 47; Table S1) on YPG at 30°C.
(C) Fitness test of the SynXIV intermediate strain J1.8 (strain 40; Table S1) with and without *TAR1* expression from its native promoter on the pRS413 plasmid in YPG at 30°C and 37°C. BY4741 (wild-type, strain 1; Table S1) transformed with empty pRS413 plasmid is shown as a control. Photos were taken after 5 days and are representative of repeated experiments. The image in (A) was made using Biorender.com.

ALE restores respiratory growth through increased *TAR1* copy number

Although we were able to use rational debugging and backcrossing to construct and restore wild-type growth to the synXIV strain, we also explored the potential of ALE to restore wild-type fitness in one of the intermediate strains afflicted with a particularly difficult to solve growth defect. Strain 40 (J1.8) had a series of background genomic mutations that potentially arose from the strain's life-history, presumably without mitochondrial DNA (Figure 2), which we solved via wild-type backcrossing and selecting a fit haploid to proceed with. In order to explore the potential of ALE to solve complex fitness defects during synthetic chromosome construction, strain 40 (J1.8; Table S1) was evolved in liquid YPG medium in triplicate for approximately 90 generations by passaging into fresh medium every 24 h (Figure 6A). The wild-type BY4741 strain was also evolved in parallel to enable the exclusion of mutations that enhance glycerol utilization, or mutations related to general adaptation to YP media, and to assess the accumulation of "hitch-hiker" mutations occurring due to genetic drift under these conditions. Initially, each passage was inoculated at an A_{600} of 0.1, but inoculation density was decreased to 0.05 after 24 generations to enable faster accrual of generations and DNA replication errors. A_{600} was measured after each 24-h period to serve as a proxy for fitness. The J1.8 strain showed a 50% improvement in final A_{600} relative to the ancestral J1.8 strain on YPG medium after 90 generations, whereas the wild-type control strain showed only a 38% improvement after 120 generations (Figure S5). Fitness testing of the wild-type strain (BY4741), parental synXIV strain (J1.8), and a mixed population from one of the J1.8 evolutionary lineages (J1.8e3i) revealed that growth on YPG was restored to wild-type levels (Figure 6B).

Whole-genome sequencing of isolates and final evolved mixed populations was carried out to compare mutations that might have caused the initial growth defect during the construction of synXIV. While no point mutations were detected anywhere in the genomes of the J1.8 evolved lineages that were absent from the control lineages, they did have a higher relative copy number of the ribosomal DNA repeat located on chromosome XII, with the evolved J1.8 lineages sharing approximately eight more rDNA copies compared to the parental J1.8 strain. The *TAR1* gene is encoded antisense to the *RDN25-1* gene on the rDNA locus, and plays a role in the quality control of defective mitochondria,[29] particularly when mixed populations of defective and functional mitochondrial populations are inherited after mating.[30] The Tar1p response to defective mitochondria is mediated via the formation of extra-chromosomal rDNA circles (ERCs), which relieve *TAR1* expression from Sir2p-mediated repression by physically locating the gene away from the native chromosomal

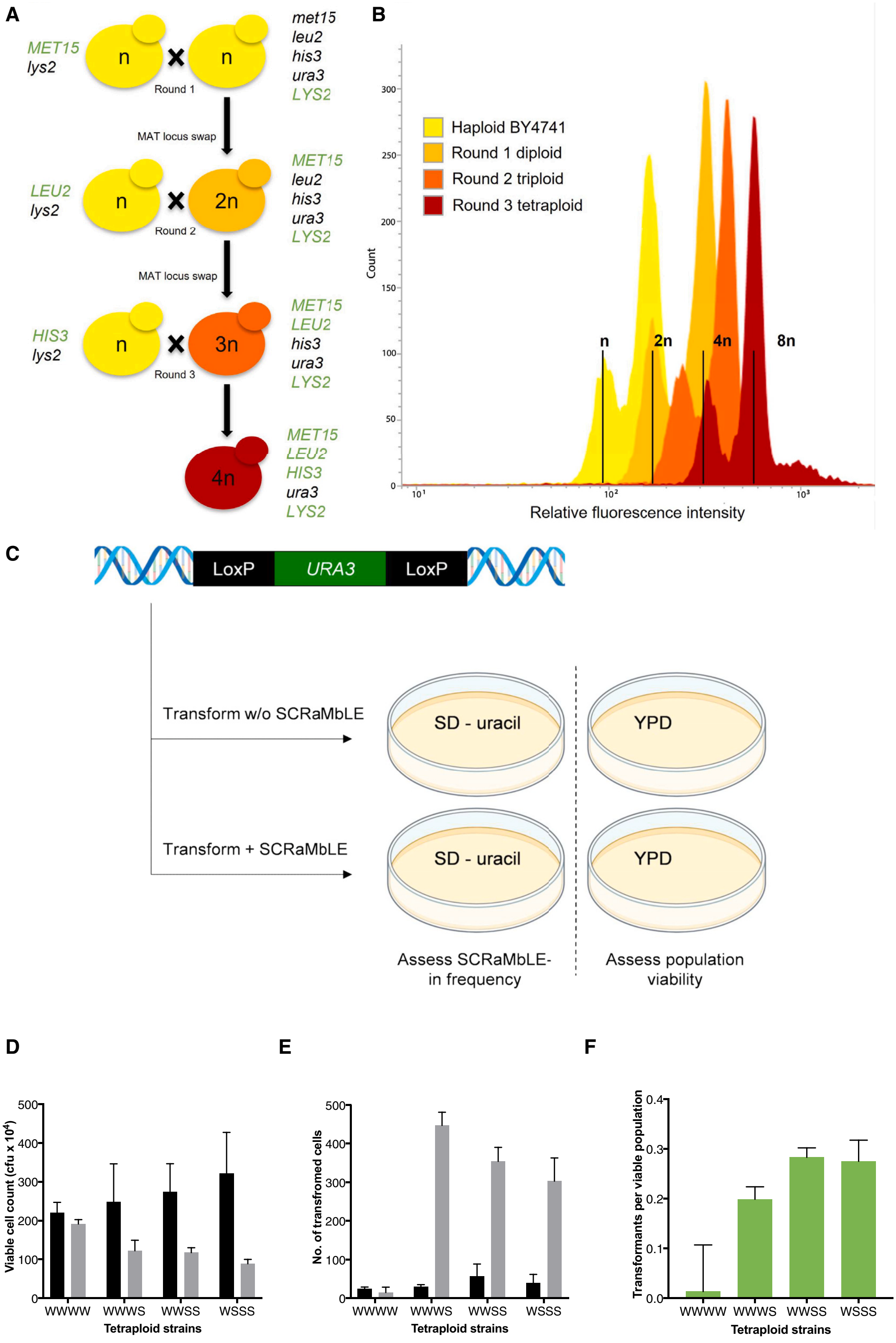

(legend on next page)

locus.[30,31] This process occurs as a result of the yeast retrograde response, which facilitates glutamate synthesis in the absence of a complete tricarboxylic acid (TCA) cycle in defective mitochondria.[32] Long-read nanopore sequencing and *de novo* assembly of evolved J1.8 isolate genomes resulted not only in full-length contigs for each of the 16 chromosomes (File S3) but also in additional contigs containing *TAR1*-rDNA repeats. These extra rDNA repeats were not observed in the genome sequences of evolved wild-type populations and were not contiguous with the chromosome XII sequence, suggesting that they represent extra *TAR1* copies presumably in the form of ERCs in the J1.8 evolved isolates. This phenomena of circular extra-chromosomal *TAR1*-encoding circular DNA has previously been observed in yeast cells with defective mitochondria.[33] Expression of the *TAR1* gene from its native promoter on a pRS413 vector improved growth of the parental synXIV strain on YPG at 30°C and 37°C (Figure 6C), suggesting that increased rDNA copy number enabled higher *TAR1* expression and normal respiratory growth in the evolved lineages.

Design and construction of synthetic-wild-type polyploid hybrids

The SCRaMbLE system is limited by the deletion of essential genes, and a subsequent reduction of viable cells in a population by over 100-fold after 24 h of induction.[15] This limitation is particularly important when the phenotype of interest cannot be easily screened for, or when SCRaMbLE is used to incorporate foreign DNA, a process referred to as SCRaMbLE-in.[20] In theory, SCRaMbLE-ing in heterologous DNA flanked by *loxP*sym sites would enable integration of genes of interest in addition to synthetic genome rearrangements. Integration of heterologous genes is highly desirable for certain phenotypes, such as cellulose degradation, where high concentrations of cellulase enzymes are required for optimal function.[34] We hypothesized that synthetic cells with higher ploidy would provide a viability buffer against the detrimental effects of essential gene loss, with the increased copy number of the synthetic chromosomes providing additional Cre-recombinase recognition sites for recombination, thereby enhancing the frequency of heterologous DNA SCRaMbLE-in. By sequential mating locus replacement and three rounds of mating (Figures 7A and 7B), strains with different combinations of native and synthetic chromosomes were isolated, except for a diploid and tetraploid strain exclusively harboring synthetic chromosomes III, VI, and IX-R. This could be due to unintended changes to gene expression levels in diploid specific genes of the synthetic chromosomes.[35]

SCRaMbLE-in of a *loxP*sym-flanked *URA3* gene (Figure 7C) in haploid yeast cells with synthetic chromosomes III, VI, and IXR[6] did not result in a significantly greater amount of transformants relative to a non-SCRaMbLE control culture (Figure S6) (two-sided Student's t test with $p > 0.05$). However, when the same experiment was carried out using diploids that had synthetic and wild-type copies of chromosomes III, VI, and IXR, there was a dramatic increase in *loxP*sym-*URA3*-*loxP*sym integration relative to a no-SCRaMbLE control (Figure S6). This indicated that the wild-type chromosome copies provided genetic redundancy to reduce the effect of essential gene loss, and that the creation of synthetic-wild-type hybrid polyploid strains may provide a mechanism for mitigating the limitations of haploid SCRaMbLE.

To further explore the effect of synthetic-wild-type hybrid genome ploidy on SCRaMbLE, tetraploid strains (Figure 7A) were tested for post-SCRaMbLE viability after 4 h of estradiol induction. Surprisingly, there was still a significant reduction in colony formation in synthetic chromosome-carrying strains relative to the wild-type control (Figure 7D; one-way ANOVA with Dunnet adjusted p value for multiple comparisons to the fully wild-type tetraploid WWWW strain, $p < 0.05$), but the effect did not increase proportionally with the number of synthetic chromosomes present. This indicated there is still a significant viability loss even in the presence of genetic redundancy in the form of wild-type chromosomes in polyploid strains. A possible explanation for this finding could be the generation of genetically unstable aneuploid strains or the loss of the *MAT*α locus during the SCRaMbLE procedure, as we only rarely observed fully triploid strains during tetraploid strain generation.

Figure 7. Variation of synthetic and wild-type chromosome ploidy for increased heterologous DNA SCRaMbLE-in efficiency

(A) The leucine (*LEU2*) and methionine (*MET15*) auxotrophic markers were restored in haploid *URA3* deficient *MAT*a and *MAT*α versions of the wild-type and syn 3, 6, 9R strains (strains 79–84; Table S4). These were sequentially mated, resulting in haploid, diploid, triploid, and tetraploid strains with different combinations of wild-type (W) and synthetic (S) chromosomes III, VI, and IXR. Cells from each round of ploidy increase were selected on the appropriate nutrient-deficient medium based on the combined nutrient prototrophy created by the correct parent mating event. Diploid cells had their *LYS2* and *MET15* functionality restored, triploids had an additional *LEU2* restored, while tetraploid cells had an additional *HIS3* restored, resulting in cells with only ura3-auxotrophy. This was verified by PCR amplification of the *MAT* locus genes and all the relevant auxotrophic marker genes. To achieve mating of the polyploid strains, the strains' mating type was made homozygous at each step by transforming with the required mating type replacement cassette.

(B) Ploidy was verified using propidium iodide DNA staining with reference to known haploid and diploid strains. We were unable to construct a fully synthetic tetraploid (red X). Actively dividing populations produced the distinctive double-peak profiles, with the first peak of each representing the ploidy of the cells in the population and the second peak, cells in the population that have undergone karyokinesis but not yet completed cytokinesis.

(C) A *loxP*sym-flanked *URA3* expression cassette was transformed into a series of semi-synthetic tetraploid yeasts with SCRaMbLE either activated or uninduced. Transformed tetraploids were identified with selective agar plates, while the effects of SCRaMbLE on cell viability were assessed using non-selective YPD agar medium.

(D) The viability of tetraploid strains with different combination of synthetic (S) and wild-type (W) chromosomes II, VI, and IXR (strains 90 [WWWW], 91 [WWWS], 92 [WWSS], 93 [WSSS]; Table S4) were determined with estradiol induction (gray bars) and without induction (black bars) of SCRaMbLE.

(E) Comparative *URA3* transformant numbers under SCRaMbLE-induced (gray bars) and SCRaMbLE-uninduced (black bars) conditions.

(F) To visualize the transformation frequency relative to the concomitant cell viability loss during SCRaMbLE, the transformants per viable population for each strain were plotted. For all figures, results from three independent transformations are shown with bars and error bars representing mean and standard deviation, respectively. SCRaMbLE-treated and non-SCRaMbLE populations were derived from the same transformed population.

In nature, diploid organisms are protected against essential gene loss by the presence of an extra copy of each gene, yet SCRaMbLE'd semi-synthetic diploid strains had a 40% reduction in viability after 8 h of growth before recovery (Figure S7). The extra wild-type chromosomes increased the survival of the semi-synthetic diploid over a synthetic haploid strain, which had a 60% reduction in cell density after 10 h of Cre-recombinase induction. Although the S and WS strain population densities recovered over longer growth periods, the initial high cell death rates allow cells with limited SCRaMbLE events (which are more fit) to dominate, resulting in an undesired final population with low genotypic diversity.[36] The tetraploid strains displayed improved viability during SCRaMbLE, with the WSSS strain containing the largest number of synthetic chromosomes, only losing ~30% viability.

DISCUSSION

This article describes the construction and debugging of *S. cerevisiae* synthetic chromosome XIV. We also demonstrate the plasticity and redundancy of the yeast genome through alternate rational and non-rational debugging approaches and through engineering post-SCRaMbLE viability using synthetic-wild-type tetraploid strains.

The first major growth defect we discovered was caused by the insertion of a *loxP*sym site 3′ of the mitochondrial ribosomal protein encoding *MRPL19* gene (Figures 1 and 2). Like all nuclear-encoded mitochondrial proteins, the *MRPL19* protein is targeted to the mitochondria. Consistent with this, the *MRPL19* protein sequence includes a predicted mitochondrial targeting peptide signal in the N terminus.[37] In addition to protein targeting signals, many nuclear-encoded mitochondrial genes have motifs in the 3′ UTR of their mRNA that facilitate localization to the outside of mitochondria via recognition by the *PUF3* protein for co-translation and import.[26] It is noteworthy that the Puf3p-binding motif (UCUGUAAAUA[38]) is located 81 bp 3′ of the *MRPL19 loxP*sym site, meaning that the observed defect may not be mediated by disruption of Puf3p binding to *MRPL19* mRNA. Alternatively, Crg1p, Mtq2p, and Scd6p have been shown to bind *MRPL19* mRNA,[39] and it is possible that they have a role in mediating the *MRPL19*-dependent defect phenotype (Figure 2).

Backcrossing of the fully synthetic J1.8 strain to a wild-type followed by whole-genome sequencing and fitness testing of haploid progeny then led to the discovery of a series of background mutations on other chromosomes that affected fitness (Figure 3). In particular, mutations detected in ATP synthase subunit genes that have been observed by others to suppress defects associated with mitochondrial DNA loss by allowing mitochondrial protein import in petite strains, yet would be deleterious when mitochondria are present and functional.[40] The history of the synXIV strain presumably involved a long period without mitochondrial DNA, during which time the observed $ATP1^{A424T}$ and $ATP3^{I303V}$ mutations may have arisen. When the two half-chromosome strains were crossed, we generated a strain with healthy mitochondria that lacked the synthetic DNA region that initially caused mitochondrial dysfunction (*MRPL19*) (strain 37; Figure 1), meaning that the background *ATP1* and *ATP3* mutations may have switched from suppressing a growth defect to causing one.

Integration of missing synthetic DNA regions into the backcrossed synXIV.17 strain showed that deletion of the *NOG2* intron on megachunk W also resulted in a growth defect (Figures 4 and S4). Although *NOG2-GFP* fluorescence levels were similar with and without the *NOG2* intron in the SynXIV.17.W.X strain (Figure S4C), growth was only restored to wild-type levels when the intron-containing wild-type *NOG2* gene was expressed from a plasmid (Figure S3D). *NOG2* is an essential, intron-containing gene encoding a putative GTPase that facilitates pre-60S ribosomal subunit maturation and export from the nucleus.[41] The *NOG2* intron encodes a small nucleolar RNA (snoRNA) that guides pseudouridylation of large-subunit (LSU) rRNA[27] at positions that are highly conserved across bacterial and eukaryotic domains. These rRNA pseudouridylations facilitate the formation of correct ribosome structure, and the *snr191* snoRNA has previously been reported to convey a growth advantage in yeast, but is not essential.[27] The linear *NOG2* intron also accumulates under stress conditions as a stable intron, where it is disadvantageous if deleted.[28] It would be interesting to try to produce a "refactored" linear version of the snr191 snoRNA, which would in principle allow the intron to be deleted without loss of fitness.

In parallel to our rational debugging approaches (Figure 1), the defective J1.8 strain had wild-type growth restored via ALE on YPG medium, which led to increased *TAR1* copy number (Figure 2). Nanopore sequencing showed that this increased *TAR1* copy number likely occurred in the form of ERCs, which are known to accumulate in yeast with defective mitochondria.[33] *TAR1* expression at the rDNA locus is repressed by the *Sir* complex,[31] meaning that relocation of *TAR1* to ERCs could increase Tar1p expression to mediate mitochondrial activity.[30] Recent work has shown that, when defective "selfish" mitochondria are inherited after mating, the retrograde response is triggered, leading to *TAR1* amplification to repress and remove defective mitochondria from the population.[30,42] The increased rDNA copy number we observed in evolved populations could have occurred in response to defective mitochondria that were inherited from the crossing of partially synthetic strains to make synXIV (Figure S2A). Defective mitochondria would have been present in the synthetic G-X strain for many generations after the introduction of megachunk J and the growth defect associated with the *MRPL19 loxP*sym motif.

In retrospect, given the number of potentially deleterious background mutations (Figure 3) and synXIV design changes (Figure 1) that had to be reverted to restore normal growth rationally, it makes sense that our parallel ALE approach resulted in a broad suppressor mutation in the form of *TAR1*-carrying ERCs. The increasing scale and complexity of synthetic genome projects means that synthetic lethality and synthetic growth defects will likely become more common, which will increase the difficulty of rational debugging approaches. ALE is therefore set to ultimately become an even more important tool in this field, where synthetic genomes will need to be

debugged with limited prior knowledge of the genetic basis of bugs. ALE can also be used to "polish" synthetic genomes toward improved industrial fitness, as recently demonstrated in a recoded *Escherichia coli* genome.[43,44] However, our results show that ,when multiple bugs are present, an ALE approach may result in broad suppressor mutations that are effective yet do not fully resolve the underlying "original" genetic basis of the poor growth phenotype. Whether evolved suppressor mutations can be tolerated will depend on individual project goals and parameters and whether rational debugging is technically feasible.

Conclusions

We successfully constructed and debugged synXIV as part of the Sc2.0 project. In the process, we discovered defects arising from a *loxP*sym site interfering with mitochondrial protein delivery, intron removal, and the accumulation of background mutations. We also demonstrated for the first time that a strain with a synthetic eukaryotic chromosome can be debugged using ALE, but that, in our case, this led to the selection of a broad suppressor mutation in the form of increased *TAR1* copy number, representing a different solution compared to our rational approaches. In addition to the construction and debugging of synXIV, we further developed the Sc 2.0 SCRaMbLE system to improve post-SCRaMbLE viability by engineering synthetic-wild-type tetraploid hybrid strains. The increased number of synthetic chromosomes within the tetraploid strains allowed a greater variety of recombination events through more *loxP*sym sites to facilitate SCRaMbLE-in (Figures 7 and S6), while promoting the recovery of a more diverse post-SCRaMbLE cell population. These synthetic-wild-type tetraploid strains further enhance the genetic redundancy and therefore the potential genotypic diversity of SCRaMbLE'd populations, in line with previous work on synthetic-wild-type diploid strains.[18,20] Together with our exploration of parallel rational and non-rational growth-defect debugging, our results on tetraploid SCRaMbLE-ing demonstrate the extreme plasticity of synthetic genomes to both designer and random changes in gene content and genomic architecture.

Limitations of the study

The construction and debugging of *S. cerevisiae* synthetic chromosome XIV involved the parallel use of laboratory evolution and rational techniques, which led to different solutions. This outcome will likely depend on the complexity of the bugs that exist in a strain and will not necessarily hold true for each analogous situation. Our finding of Mrpl19p mislocalization relative to the mitochondria due to the presence of a 3′ UTR *loxP*sym site is based on qualitative fluorescence data using confocal microscopy. This phenomenon could be further investigated through isolation of the mitochondria with subsequent fluorescence or proteomics analysis. Our analysis of post-SCRaMbLE viability in synthetic-wild-type tetraploid hybrid strains could be improved through long-read whole-genome sequencing to prove that rearrangements are more common in these hybrids than in haploids and that this approach improves genome plasticity.

CONSORTIA

This work is part of the international Synthetic Yeast Genome (Sc2.0) consortium. The chromosome design and building consortium includes research groups worldwide: Boeke Lab at Johns Hopkins University and New York University (led chromosomes I, III, IV, VI, VIII, IX), Chandrasegaran lab at Johns Hopkins (led chromosomes III and IX), Cai Lab at University of Edinburgh and University of Manchester (led chromosomes II, VII and tRNA neochromosome), Yue Shen's team at BGI-Research SHENZHEN (led chromosomes II, VII, XIII), Y.J. Yuan's team at Tianjin University (led chromosome V, X), Dai Lab at Tsinghua University and Shenzhen Institute of Advanced Technology, CAS (led chromosome XII), Ellis Lab at Imperial College London (led chromosome XI), Sakkie Pretorius's team at Macquarie University (led chromosomes XIV, XVI), Matthew Wook Chang's team at National University of Singapore (led chromosome XV), Bader and Boeke Labs at Johns Hopkins University (led design and workflow), and Build-A-Genome undergraduate teams at Johns Hopkins University and Loyola University Maryland (contributed to chromosomes I, III, IV, VIII, IX). The Sc2.0 consortium includes numerous other participants and are acknowledged on the project web site www.syntheticyeast.org.

STAR★METHODS

Detailed methods are provided in the online version of this paper and include the following:

- KEY RESOURCES TABLE
- RESOURCE AVAILABILITY
  - Lead contact
  - Materials availability
  - Data and code availability
- EXPERIMENTAL MODEL AND SUBJECT DETAILS
- METHOD DETAILS
  - Growth media
  - Growth conditions
  - Chunk preparation
  - Yeast transformation and marker-loss screening
  - DNA extraction and PCR-tag analysis
  - CRISPR-Cas9 mediated genome modification
  - Fitness testing
  - SynXIV discrepancy repair
  - *MRPL19*-GFP fusion protein design
  - *TAR1* expression construct cloning
  - *NOG2-GFP* expression construct design and cloning
  - tRNA-array design and cloning
  - Confocal microscopy
  - Diploid formation
  - Sporulation, random spore isolation, and random spore screening
  - RNA extraction
  - RT-qPCR
  - Whole-genome sequencing
  - Nanopore sequencing
  - Flow cytometry

- ○ Polyploid strain construction
- ○ Ploidy determination
- ○ SCRaMbLE-in
- ○ Growth analysis of the tetraploid strains
- QUANTIFICATION AND STATISTICAL ANALYSIS

SUPPLEMENTAL INFORMATION

Supplemental information can be found online at https://doi.org/10.1016/j.xgen.2023.100379.

ACKNOWLEDGMENTS

The synthetic biology initiative at Macquarie University is financially supported by an internal grant from the university and external grants from Bioplatforms Australia, the New South Wales (NSW) Chief Scientist and Engineer, the ARC Centre of Excellence in Synthetic Biology, the Australian Genome Foundry, and the NSW Government's Department of Primary Industries. I.T.P. is supported by an Australian Research Council Laureate Fellowship. T.C.W. and B.L. were supported by Fellowships from the CSIRO Synthetic Biology Future Science Platform and Macquarie University. T.C.W. and I.S.P. acknowledge the support of ARC Discovery Project DP200100717. B.L. acknowledges the support of the Gordon and Betty Moore Foundation (GBMF9319, grant DOI: https://doi.org/10.37807/GBMF9319), the ARC Centre of Excellence for Synthetic Biology, Twist Bioscience, and the Allen Foundation. Work in the J.D.B. lab was supported by US NSF grants MCB-766 1026068, MCB-1443299, MCB-1616111, and MCB-1921641. Work in the J.S.B. lab was supported by US NSF awards MCB-1445545 and EF-1935355.

AUTHOR CONTRIBUTIONS

J.D.B., I.S.P., D.L.J., Y.C., N.C., T.C.W., I.T.P., T.R.M., L.A.M., and J.S.B. conceived and coordinated the project. T.C.W., I.T.P., X.X., H.K., T.R.M., I.W.D., A.R.B., H.K.N., B.L., N.V.W., T.R.V.C., F.M., I.T.P., R.S.K.W., Y.C., H.D.G., G.S., and J.S.B. designed experiments. T.C.W., H.K., X.X., E.L.I.W., M.I.E., I.W.D., A.R.B., B.L., N.V.W., T.R.V.C., F.M., E.L.D., R.S.K.W., A.C.C., and H.D.G. conducted experiments. T.C.W., H.K., X.X., N.V.W., A.R.B., B.L., E.L.I.W., M.I.E., H.D.G., G.S., T.R.V.C., and F.M. analyzed data. T.C.W., H.K., I.T.P., I.S.P., and J.D.B. wrote the manuscript. All authors revised and approved the final manuscript.

DECLARATION OF INTERESTS

T.C.W. and A.C.C. are founders and shareholders of Number 8 Bio Pty Ltd. I.T.P. is an advisor of Number 8 Bio Pty Ltd. J.D.B. is a founder and director of CDI Labs, Inc.; a founder of and consultant to Neochromosome, Inc; a founder, SAB member of, and consultant to ReOpen Diagnostics, LLC; and serves or served on the Scientific Advisory Board of the following: Sangamo, Inc., Modern Meadow, Inc., Rome Therapeutics, Inc., Sample6, Inc., Tessera Therapeutics, Inc. and the Wyss Institute. J.S.B. is a founder of Neochromosome, Inc. and a consultant to Opentrons Labworks, Inc. L.A.M. is a founder of Neochromosome, Inc. and an employee of Opentrons Labworks, Inc.

Received: August 4, 2022
Revised: July 17, 2023
Accepted: July 19, 2023
Published: November 8, 2023

REFERENCES

1. Pretorius, I.S. (2017). Synthetic genome engineering forging new frontiers for wine yeast. Crit. Rev. Biotechnol. *37*, 112–136. https://doi.org/10.1080/07388551.2016.1214945.
2. Pretorius, I.S., and Boeke, J.D. (2018). Yeast 2.0—connecting the dots in the construction of the world's first functional synthetic eukaryotic genome. FEMS Yeast Res. *18*, foy032. https://doi.org/10.1093/femsyr/foy032.
3. Xu, X., Meier, F., Blount, B.A., Pretorius, I.S., Ellis, T., Paulsen, I.T., and Williams, T.C. (2023). Trimming the genomic fat: minimising and re-functionalising genomes using synthetic biology. Nat. Commun. *14*, 1984. https://doi.org/10.1038/s41467-023-37748-7.
4. Richardson, S.M., Mitchell, L.A., Stracquadanio, G., Yang, K., Dymond, J.S., DiCarlo, J.E., Lee, D., Huang, C.L.V., Chandrasegaran, S., Cai, Y., et al. (2017). Design of a synthetic yeast genome. Science *355*, 1040–1044. https://doi.org/10.1126/science.aaf4557.
5. Wu, Y., Li, B.-Z., Zhao, M., Mitchell, L.A., Xie, Z.-X., Lin, Q.-H., Wang, X., Xiao, W.-H., Wang, Y., Zhou, X., et al. (2017). Bug mapping and fitness testing of chemically synthesized chromosome X. Science *355*, eaaf4706.
6. Mitchell, L.A., Wang, A., Stracquadanio, G., Kuang, Z., Wang, X., Yang, K., Richardson, S., Martin, J.A., Zhao, Y., Walker, R., et al. (2017). Synthesis, debugging, and effects of synthetic chromosome consolidation: synVI and beyond. Science *355*, eaaf4831.
7. Shen, Y., Wang, Y., Chen, T., Gao, F., Gong, J., Abramczyk, D., Walker, R., Zhao, H., Chen, S., Liu, W., et al. (2017). Deep functional analysis of synII, a 770-kilobase synthetic yeast chromosome. Science *355*, eaaf4791.
8. Xie, Z.-X., Li, B.-Z., Mitchell, L.A., Wu, Y., Qi, X., Jin, Z., Jia, B., Wang, X., Zeng, B.-X., Liu, H.-M., et al. (2017). "Perfect" designer chromosome V and behavior of a ring derivative. Science *355*, eaaf4704.
9. Zhang, W., Zhao, G., Luo, Z., Lin, Y., Wang, L., Guo, Y., Wang, A., Jiang, S., Jiang, Q., Gong, J., et al. (2017). Engineering the ribosomal DNA in a megabase synthetic chromosome. Science *355*, eaaf3981.
10. Mercy, G., Mozziconacci, J., Scolari, V.F., Yang, K., Zhao, G., Thierry, A., Luo, Y., Mitchell, L.A., Shen, M., Shen, Y., et al. (2017). 3D organization of synthetic and scrambled chromosomes. Science *355*, eaaf4597.
11. Annaluru, N., Muller, H., Mitchell, L.A., Ramalingam, S., Stracquadanio, G., Richardson, S.M., Dymond, J.S., Kuang, Z., Scheifele, L.Z., Cooper, E.M., et al. (2014). Total Synthesis of a Functional Designer Eukaryotic Chromosome. Science *344*, 55–58. https://doi.org/10.1126/science.1249252.
12. Dymond, J., and Boeke, J. (2012). The Saccharomyces cerevisiae SCRaMbLE system and genome minimization. Bioeng. Bugs *3*, 168–171. https://doi.org/10.4161/bbug.19543.
13. Dymond, J.S., Richardson, S.M., Coombes, C.E., Babatz, T., Muller, H., Annaluru, N., Blake, W.J., Schwerzmann, J.W., Dai, J., Lindstrom, D.L., et al. (2011). Synthetic chromosome arms function in yeast and generate phenotypic diversity by design. Nature *477*, 471–476. http://www.nature.com/nature/journal/v477/n7365/abs/nature10403.html#supplementary-information.
14. Jovicevic, D., Blount, B.A., and Ellis, T. (2014). Total synthesis of a eukaryotic chromosome: Redesigning and SCRaMbLE-ing yeast. Bioessays *36*, 855–860. https://doi.org/10.1002/bies.201400086.
15. Shen, Y., Stracquadanio, G., Wang, Y., Yang, K., Mitchell, L.A., Xue, Y., Cai, Y., Chen, T., Dymond, J.S., Kang, K., et al. (2016). SCRaMbLE generates designed combinatorial stochastic diversity in synthetic chromosomes. Genome Res. *26*, 36–49. https://doi.org/10.1101/gr.193433.115.
16. Vickers, C.E., Williams, T.C., Peng, B., and Cherry, J. (2017). Recent advances in synthetic biology for engineering isoprenoid production in yeast. Curr. Opin. Chem. Biol. *40*, 47–56. https://doi.org/10.1016/j.cbpa.2017.05.017.
17. Williams, T.C., Pretorius, I.S., and Paulsen, I.T. (2016). Synthetic Evolution of Metabolic Productivity Using Biosensors. Trends Biotechnol. *34*, 371–381. https://doi.org/10.1016/j.tibtech.2016.02.002.
18. Jia, B., Wu, Y., Li, B.-Z., Mitchell, L.A., Liu, H., Pan, S., Wang, J., Zhang, H.-R., Jia, N., Li, B., et al. (2018). Precise control of SCRaMbLE in synthetic haploid and diploid yeast. Nat. Commun. *9*, 1933. https://doi.org/10.1038/s41467-018-03084-4.
19. Shen, M.J., Wu, Y., Yang, K., Li, Y., Xu, H., Zhang, H., Li, B.-Z., Li, X., Xiao, W.-H., Zhou, X., et al. (2018). Heterozygous diploid and interspecies

SCRaMbLEing. Nat. Commun. *9*, 1934. https://doi.org/10.1038/s41467-018-04157-0.

20. Liu, W., Luo, Z., Wang, Y., Pham, N.T., Tuck, L., Pérez-Pi, I., Liu, L., Shen, Y., French, C., Auer, M., et al. (2018). Rapid pathway prototyping and engineering using in vitro and in vivo synthetic genome SCRaMbLE-in methods. Nat. Commun. *9*, 1936. https://doi.org/10.1038/s41467-018-04254-0.
21. Merz, S., and Westermann, B. (2009). Genome-wide deletion mutant analysis reveals genes required for respiratory growth, mitochondrial genome maintenance and mitochondrial protein synthesis in Saccharomyces cerevisiae. Genome Biol. *10*, R95. https://doi.org/10.1186/gb-2009-10-9-r95.
22. Parisi, M.A., Xu, B., and Clayton, D.A. (1993). A human mitochondrial transcriptional activator can functionally replace a yeast mitochondrial HMG-box protein both in vivo and in vitro. Mol. Cell Biol. *13*, 1951–1961. https://doi.org/10.1128/MCB.13.3.1951.
23. Williams, T.C., Averesch, N.J.H., Winter, G., Plan, M.R., Vickers, C.E., Nielsen, L.K., and Krömer, J.O. (2015). Quorum-sensing linked RNA interference for dynamic metabolic pathway control in Saccharomyces cerevisiae. Metab. Eng. *29*, 124–134. https://doi.org/10.1016/j.ymben.2015.03.008.
24. Williams, T.C., Xu, X., Ostrowski, M., Pretorius, I.S., and Paulsen, I.T. (2017). Positive-feedback, ratiometric biosensor expression improves high-throughput metabolite-producer screening efficiency in yeast. Synth. Biol. *2*, ysw002. https://doi.org/10.1093/synbio/ysw002.
25. Peng, B., Williams, T.C., Henry, M., Nielsen, L.K., and Vickers, C.E. (2015). Controlling heterologous gene expression in yeast cell factories on different carbon substrates and across the diauxic shift: a comparison of yeast promoter activities. Microb. Cell Fact. *14*, 91. https://doi.org/10.1186/s12934-015-0278-5.
26. Saint-Georges, Y., Garcia, M., Delaveau, T., Jourdren, L., Le Crom, S., Lemoine, S., Tanty, V., Devaux, F., and Jacq, C. (2008). Yeast Mitochondrial Biogenesis: A Role for the PUF RNA-Binding Protein Puf3p in mRNA Localization. PLoS One *3*, e2293. https://doi.org/10.1371/journal.pone.0002293.
27. Badis, G., Fromont-Racine, M., and Jacquier, A. (2003). A snoRNA that guides the two most conserved pseudouridine modifications within rRNA confers a growth advantage in yeast. RNA *9*, 771–779. https://doi.org/10.1261/rna.5240503.
28. Morgan, J.T., Fink, G.R., and Bartel, D.P. (2019). Excised linear introns regulate growth in yeast. Nature *565*, 606–611. https://doi.org/10.1038/s41586-018-0828-1.
29. Bonawitz, N.D., Chatenay-Lapointe, M., Wearn, C.M., and Shadel, G.S. (2008). Expression of the rDNA-encoded mitochondrial protein Tar1p is stringently controlled and responds differentially to mitochondrial respiratory demand and dysfunction. Curr. Genet. *54*, 83–94. https://doi.org/10.1007/s00294-008-0203-0.
30. Poole, A.M., Kobayashi, T., and Ganley, A.R.D. (2012). A positive role for yeast extrachromosomal rDNA circles? Bioessays *34*, 725–729. https://doi.org/10.1002/bies.201200037.
31. Li, C., Mueller, J.E., and Bryk, M. (2006). Sir2 represses endogenous polymerase II transcription units in the ribosomal DNA nontranscribed spacer. Mol. Biol. Cell *17*, 3848–3859. https://doi.org/10.1091/mbc.e06-03-0205.
32. Jazwinski, S.M., and Kriete, A. (2012). The Yeast Retrograde Response as a Model of Intracellular Signaling of Mitochondrial Dysfunction. Front. Physiol. *3*, 139. https://doi.org/10.3389/fphys.2012.00139.
33. Borghouts, C., Benguria, A., Wawryn, J., and Jazwinski, S.M. (2004). Rtg2 Protein Links Metabolism and Genome Stability in Yeast Longevity. Genetics *166*, 765–777. https://doi.org/10.1093/genetics/166.2.765.
34. Kroukamp, H., den Haan, R., van Zyl, J.-H., and van Zyl, W.H. (2017). Rational strain engineering interventions to enhance cellulase secretion by Saccharomyces cerevisiae. Biofuel. Bioprod. Biorefin. *12*, 108–124. https://doi.org/10.1002/bbb.1824.
35. Strome, E.D., Wu, X., Kimmel, M., and Plon, S.E. (2008). Heterozygous screen in Saccharomyces cerevisiae identifies dosage-sensitive genes that affect chromosome stability. Genetics *178*, 1193–1207. https://doi.org/10.1534/genetics.107.084103.
36. Wightman, E.L.I., Kroukamp, H., Pretorius, I.S., Paulsen, I.T., and Nevalainen, H.K.M. (2020). Rapid Colorimetric Detection of Genome Evolution in SCRaMbLEd Synthetic Saccharomyces cerevisiae Strains. Microorganisms *8*, 1914. https://doi.org/10.3390/microorganisms8121914.
37. Fukasawa, Y., Tsuji, J., Fu, S.-C., Tomii, K., Horton, P., and Imai, K. (2015). MitoFates: improved prediction of mitochondrial targeting sequences and their cleavage sites. Mol. Cell. Proteomics *14*, 1113–1126. https://doi.org/10.1074/mcp.M114.043083.
38. Lapointe, C.P., Stefely, J.A., Jochem, A., Hutchins, P.D., Wilson, G.M., Kwiecien, N.W., Coon, J.J., Wickens, M., and Pagliarini, D.J. (2018). Multi-omics Reveal Specific Targets of the RNA-Binding Protein Puf3p and Its Orchestration of Mitochondrial Biogenesis. Cell Syst. *6*, 125–135.e6. https://doi.org/10.1016/j.cels.2017.11.012.
39. Tsvetanova, N.G., Klass, D.M., Salzman, J., and Brown, P.O. (2010). Proteome-Wide Search Reveals Unexpected RNA-Binding Proteins in Saccharomyces cerevisiae. PLoS One *5*, e12671. https://doi.org/10.1371/journal.pone.0012671.
40. van Leeuwen, C., Destrac-Irvine, A., Dubernet, M., Duchêne, E., Gowdy, M., Marguerit, E., Pieri, P., Parker, A., de Rességuier, L., and Ollat, N. (2019). An Update on the Impact of Climate Change in Viticulture and Potential Adaptations. Agronomy *9*, 514. https://doi.org/10.3390/agronomy9090514.
41. Saveanu, C., Bienvenu, D., Namane, A., Gleizes, P.E., Gas, N., Jacquier, A., and Fromont-Racine, M. (2001). Nog2p, a putative GTPase associated with pre-60S subunits and required for late 60S maturation steps. EMBO J. *20*, 6475–6484.
42. Walker, M. (2015). The Function of TAR1 and the Evolution of the Retrograde Response.
43. Ostrov, N., Landon, M., Guell, M., Kuznetsov, G., Teramoto, J., Cervantes, N., Zhou, M., Singh, K., Napolitano, M.G., Moosburner, M., et al. (2016). Design, synthesis, and testing toward a 57-codon genome. Science *353*, 819–822. https://doi.org/10.1126/science.aaf3639.
44. Wannier, T.M., Kunjapur, A.M., Rice, D.P., McDonald, M.J., Desai, M.M., Church, G.M., and Church, G.M. (2018). Adaptive evolution of genomically recoded Escherichia coli</i&gt. Proc. Natl. Acad. Sci. USA *115*, 3090–3095. https://doi.org/10.1073/pnas.1715530115.
45. Gietz, R.D., and Schiestl, R.H. (2007). Quick and easy yeast transformation using the LiAc/SS carrier DNA/PEG method. Nat. Protoc. *2*, 35–37.
46. Lõoke, M., Kristjuhan, K., and Kristjuhan, A. (2011). Extraction of genomic DNA from yeasts for PCR-based applications. Biotechniques *50*, 325–328. https://doi.org/10.2144/000113672.
47. Pédelacq, J.D., Cabantous, S., Tran, T., Terwilliger, T.C., and Waldo, G.S. (2005). Engineering and characterization of a superfolder green fluorescent protein. Nat. Biotechnol. *24*, 79–88. https://doi.org/10.1038/nbt1172.
48. Waterhouse, A., Bertoni, M., Bienert, S., Studer, G., Tauriello, G., Gumienny, R., Heer, F.T., de Beer, T.A.P., Rempfer, C., Bordoli, L., et al. (2018). SWISS-MODEL: homology modelling of protein structures and complexes. Nucleic Acids Res. *46*, W296-w303. https://doi.org/10.1093/nar/gky427.
49. Jores, T., Klinger, A., Groß, L.E., Kawano, S., Flinner, N., Duchardt-Ferner, E., Wöhnert, J., Kalbacher, H., Endo, T., Schleiff, E., and Rapaport, D. (2016). Characterization of the targeting signal in mitochondrial β-barrel proteins. Nat. Commun. *7*, 12036. https://doi.org/10.1038/ncomms12036.
50. Edwards, W.R., Busse, K., Allemann, R.K., and Jones, D.D. (2008). Linking the functions of unrelated proteins using a novel directed evolution domain insertion method. Nucleic Acids Res. *36*, e78. https://doi.org/10.1093/nar/gkn363.

51. Teste, M.-A., Duquenne, M., François, J.M., and Parrou, J.-L. (2009). Validation of reference genes for quantitative expression analysis by real-time RT-PCR in Saccharomyces cerevisiae. BMC Mol. Biol. *10*, 99.

52. Kearse, M., Moir, R., Wilson, A., Stones-Havas, S., Cheung, M., Sturrock, S., Buxton, S., Cooper, A., Markowitz, S., Duran, C., et al. (2012). Geneious Basic: an integrated and extendable desktop software platform for the organization and analysis of sequence data. Bioinformatics *28*, 1647–1649. https://doi.org/10.1093/bioinformatics/bts199.

53. Rosebrock, A.P. (2017). Analysis of the Budding Yeast Cell Cycle by Flow Cytometry. Cold Spring Harb. Protoc. *2017*. pdb.prot088740.

STAR★METHODS

KEY RESOURCES TABLE

REAGENT or RESOURCE	SOURCE	IDENTIFIER
Bacterial and virus strains		
NEB 5-alpha Competent E. coli (High Efficiency)	New England Biolabs	C2987H
Chemicals, peptides, and recombinant proteins		
Mitotracker Red FM	ThermoFisher	M22425
B-Estradiol	Merck	E8875
hygromycin B	Merck	10843555001
Deposited data		
SynXIV 29 I J1.4 raw genome sequencing data	This study	Biosample: SAMN28591717
SynXIV 29 I J1.8 raw genome sequencing data	This study	Biosample: SAMN28591718
BYe1 raw genome sequencing data	This study	Biosample: SAMN28591711
BYe2 raw genome sequencing data	This study	Biosample: SAMN28591712
BYe3 raw genome sequencing data	This study	Biosample: SAMN28591713
J1.8e1 raw genome sequencing data	This study	Biosample: SAMN28591714
J1.8e2 raw genome sequencing data	This study	Biosample: SAMN28591715
J1.8e3 raw genome sequencing data	This study	Biosample: SAMN28591716
SynXIV.17c NOG2 wt, A2-A3, V4, V1, R1, *YNL114W*, Chr12, K3, *YNL116W*^{L697I}, E3 raw genome sequencing data	This study	Biosample: SAMN28591719
Experimental models: Organisms/strains		
BY4741	Euroscarf	Strain 1, Table S1
SynXIV 29 I J1.4	This study	Strain 39, Table S1
SynXIV 29 I J1.8	This study	Strain 40, Table S1
BYe1	This study	Strain 41, Table S1
BYe2	This study	Strain 42, Table S1
BYe3	This study	Strain 43, Table S1
J1.8e1	This study	Strain 44, Table S1
J1.8e2	This study	Strain 45, Table S1
J1.8e3	This study	Strain 46, Table S1
SynXIV 7c	This study	Strain 52, Table S1
SynXIV 12c	This study	Strain 53, Table S1
SynXIV.17	This study	Strain 55, Table S1
SynXIV.17.c	This study	Strain 60, Table S1
SynXIV.17.c NOG2 wt	This study	Strain 63, Table S1
SynXIV.17c NOG2 wt, A2-A3, M3	This study	Strain 68, Table S1
SynXIV.17c NOG2 wt, A2-A3, V4, V1, *YNL114W*	This study	Strain 69, Table S1
SynXIV.17c NOG2 wt, A2-A3, V4, V1, R1, *YNL114W*, Chr12, K3, *YNL116W*^{L697I}, E3, R1	This study	Strain 78, Table S1
WWWW	This study	Strain 90, Table S4
WWWS	This study	Strain 91, Table S4
WWSS	This study	Strain 92, Table S4
WSSS	This study	Strain 93, Table S4
Oligonucleotides		
ACTCCACTTCAAGTAAGAGTTTG	Integrated DNA Technologies	Mat-A_F
GCACGGAATATGGGACTACTTCG	Integrated DNA Technologies	Mat-alpha_F

(Continued on next page)

Continued

REAGENT or RESOURCE	SOURCE	IDENTIFIER
AGTCACATCAAGATCGTTTATGG	Integrated DNA Technologies	Mat-locus_R
AGCTTGGTGAGCGCTAGGGAG	Integrated DNA Technologies	his3up-F
GTTCTTACGGAATACCACTTGCC	Integrated DNA Technologies	his3up-R
AACCGGCTTTTCATATAGAATAGAGAAGC	Integrated DNA Technologies	leu2up-F
GAGGTCGACTACGTCGTTAAGG	Integrated DNA Technologies	leu2up-R
Recombinant DNA		
pHK-Cre-EBDh	This study	pHK-Cre-EBDh
TAR1-pRS413	This study	TAR1-pRS413
MRPL19-GFP-MRPL19-LoxP-pRS416	This study	MRPL19-GFP-MRPL19-LoxP-pRS416
MRPL19-GFP-MRPL19-Native-pRS416	This study	MRPL19-GFP-MRPL19-Native-pRS416
NOG2wt-GFP-pRS416	This study	NOG2wt-GFP-pRS416
NOG2syn-GFP-pRS416	This study	NOG2syn-GFP-pRS416
Software and algorithms		
Image J	National Institutes of Health	github.com/imagej/ImageJ
Prism 9	GraphPad	GraphPad Prism 9.3.1
Geneious Prime	Biomatters Ltd	2022.1.1
Albacore	www.albacorebuild.net	Albacore v2.3.1
Porechop	https://github.com/rrwick/Porechop	Porechop v0.2.3
Canu	https://github.com/marbl/canu	Canu v1.7.1
Nanopolish	https://github.com/jts/nanopolish	Nanopolish 0.10.1
Microsoft Powerpoint	Microsoft Office Professional Plus 2019	Version 1808
Microsoft Excel	Microsoft Office Professional Plus 2019	Version 1808
Guppy	Oxford nanopore Technologies	v4.2.3

RESOURCE AVAILABILITY

Lead contact

Further information and requests for resources and reagents should be directed to and will be fulfilled by the lead contact, Ian Paulsen (ian.paulsen@mq.edu.au).

Materials availability

All plasmids and yeast strains generated during this study are available on request.

Data and code availability

- Genome sequencing data has been deposited at GenBank: BioProject ID PRJNA841391 and will be made publicly available as of the date of publication. Accession numbers will be listed in the key resources table. Microscopy data reported in this paper will be shared by the lead contact upon request.
- This paper does not report original code.
- Any additional information required to reanalyze the data reported in this paper is available from the lead contact upon request.

EXPERIMENTAL MODEL AND SUBJECT DETAILS

S. cerevisiae strains (Tables S1 and S4) are all derivatives of BY4741 (MATa *his3Δ1 leu2Δ0 met15Δ0 ura3Δ0*), a haploid auxotrophic laboratory strain of mating type 'a'. Yeast strains were grown in synthetic dropout (SD) media containing Yeast Nitrogen Base Without Amino Acids mix (Sigma-Aldrich Y0626) supplemented with 10 g/L glucose, and amino acids at 100 mg/L to complement auxotrophies as appropriate. Alternatively, yeast were grown in 10 g/L yeast extract, 20 g/L peptone, 20 g/L dextrose (YPD), or YP-glycerol (20 g/L glycerol in place of dextrose). *E. coli* DH5α strains were used to store and propagate plasmids (Table S3), and were grown in Lysogeny Broth medium with 50 mg/mL ampicillin.

Liquid growth of *E. coli* and *S. cerevisiae* strains was carried out in an Infors 25 mm orbital shaking incubator set to 30°C or 37°C and 200 rpm. Cells were cultured in either sterile 50 mL falcon tubes or 250 mL baffled shake-flasks where medium did not comprise more than 10 % of the total vessel volume.

METHOD DETAILS

Growth media

S. cerevisiae strains were grown in medium containing synthetic complete (SC) media containing 1x Yeast Nitrogen Base Without Amino Acids mix (Sigma-Aldrich Y0626) supplemented with 10 g/L glucose, and amino acids at 100 mg/L to complement auxotrophies as appropriate. Alternatively, yeast were grown in 10 g/L yeast extract, 20 g/L peptone, 20 g/L dextrose (YPD), or YP-glycerol (20 g/L glycerol in place of dextrose).

Growth conditions

Liquid growth was carried out in a 25 mm orbital shaking incubator (Infors Multitron Pro) set to 30°C and 200 rpm. Cells were cultured in either sterile 50 mL Falcon conical tubes or 250 mL baffled shake-flasks where medium did not comprise more than 10 % of the total vessel volume.

Chunk preparation

DNA chunks comprising ~5-10 Kb of each megachunk were synthesized and sequence verified by Genscript (megachunks A-K and N-X), GeneArt (megachunks L, M), and GeneWiz (chunks E1, E2, E3, S4). Chunks were then either restriction digested using terminal, complementary sites incorporated in the design changes, or PCR amplified using primers that anneal to the 5′ or 3′ ends of each chunk with Phusion polymerase (New England Biolabs). Plasmid digested or PCR amplified chunks were excised from agarose gels or column purified, and quantified. Chunks were pooled together such that the relative amounts of each chunk were approximately halved so that chunk 1 > chunk 2 > chunk 3 > chunk 4, with the amount of chunk 4 being 400-800 ng. Restriction digested chunks were ligated over night at 16°C using T4 DNA ligase (New England Biolabs).

Yeast transformation and marker-loss screening

Cells were transformed using the lithium acetate/polyethylene glycol/ssDNA transformation method.[45] After 2-5 d of incubation on selective media at 30°C, colonies were replica-plated onto media selective for the marker gene used to integrate the previous megachunk, with those not able to grow used for further analysis.

DNA extraction and PCR-tag analysis

Genomic DNA was extracted using a lithium acetate-SDS solution for cell disruption followed by ethanol mediated DNA precipitation as previously described.[46] Crude DNA extracts were transferred to a 384-well plate compatible with the Echo 550 acoustic liquid transfer system (Labcyte), as were primer pairs for each PCR-tag in synXIV (File S4) (15 μM). 4.75 μL aliquots of 1x SYBR green mastermix were added to each well of a 384-well qPCR plate using an epMotion liquid handling robot. 200 nL of crude gDNA and 50 nL of each primer-pair was transferred to each 384-well qPCR plate well using an Echo550 (Labcyte Inc.). The plate was centrifuged briefly to ensure transferred droplets were suspended in the SYBR-green mix. qPCR was carried out using a Lightcycler 480 with an initial 95°C denaturation of 3 minutes followed by 15 cycles of 30 s at 95°C, 30 s at 70°C with a decrease of 1°C each cycle, and extension at 72°C for 30 s. The same denaturation and extension condition were then used for a further 20 cycles, except with constant annealing at 55°C. SYBR-green fluorescence was measured at the end of each extension step. After cycling, a melt curve was generated by heating from 50°C to 95°C with fluorescence measurements every 5 s. For each megachunk, positive and negative controls were used that comprised of mixed synthetic chunk DNA or BY4741 DNA respectively. Any PCR-tags resulting in aberrant amplification were excluded from analysis of transformant DNA. Megachunk integration was accepted when all synthetic PCR-tags and no wild-type PCR-tags resulted in amplification.

CRISPR-Cas9 mediated genome modification

CRISPR-Cas9 mediated homologous recombination was carried out by using a previously reported strategy that utilizes a single episomal plasmid (pRS423) that contains both guide RNA and Cas9 expression cassettes.[24] New 20 bp guide RNA sequences were encoded in 5′ extensions of primers that target the 3′ end of the *SNR52* promoter (reverse primers) and the 5′ end of the structural CRISPR RNA (forward primer). ~ 100 ng of the linear PCR product resulting from this reaction was used to co-transform yeast with 1-5 μg of donor DNA with homology to the target guide-RNA locus. Colonies growing on SC –histidine media were screened for desired mutations using PCR-tag analysis and/or loci specific primers.

Fitness testing

Spot assay test strains were inoculated into 5 mL of medium in 50 mL Falcon conical tubes and grown overnight at 30°C with 200 rpm shaking. Each culture was then passaged to a fresh tube with 5 mL of medium at an A_{600nm} of 0.4 – 0.5 and grown for a further 3-4 h. The A_{600nm} of each culture was adjusted to be the same, and each culture was 10-fold serially diluted in sterile MilliQ water down to 10,000-fold. 3 uL of each dilution was then spotted onto the indicated agar plates and incubated at 30 or 37°C for 4 d. Plates were imaged using a Singer Phenobooth, contrasts adjusted in Microsoft Powerpoint, and each dilution series cropped, resized, and repositioned without any non-proportional resizing. Only cultures that were grown on the same plate for the same amount of time were directly compared and shown together. Each image is representative of at least two repeated experiments. Liquid growth assay

strains were precultured the same way as spot assay strains except that were inoculated into 200 uL of liquid medium in a 96-well flat-bottom plate and incubated with double orbital shaking at 269 cycles per minute at 30°C for 24 hours with A_{600} measurements taken every 15 minutes.

SynXIV discrepancy repair

As a default option, sequence discrepancies (Table S2) were repaired using our previously developed CRISPR-Cas9 system,[24] whereby synthetic chunk DNA served as donor for homologous recombination. Discrepancies 1-10 (Table S2) were repaired by targeting the *EGT2* ORF with CRISPR-Cas9 and synthetic chunks A3-A4 as donor DNA. The *EGT2* ORF was fully synonymously recoded as part of an error in the synXIV design phase, and we subsequently reverted this sequence to wild-type. However, this made no difference to fitness, so the wild-type sequence was used as a guide-RNA target during the repair of discrepancies 1-10, leaving the sequence in its original fully recoded state. Two non-TAA stop codons on the *YNL114C* and *RPC19* genes on chunk M3 were re-inserted by integrating a chemically synthesized *URA3* marker flanked by 796 bp 5′ and 1236 bp 3′ homology to the region. The *URA3* marker was then replaced by homologous integration of donor DNA with either both stop codons swapped, or with only the *RPC19* stop codon swapped (discrepancy 17, along with 18) using a *URA3*-specific CRISPR guide RNA and Cas9. Discrepancies 15, 16, and 19 were repaired using a similar approach, whereby a synthetic *URA3* marker was inserted, then replaced with PCR amplified DNA containing the desired changes. Discrepancies that were located on terminal, marker-containing or overwriting chunks (numbers 11-14 and 20-23, Table S2) were repaired by integrating the relevant chunk using selection for its marker (*LEU2* or *URA3*). The marker was then removed by targeting it using a *LEU2* or *URA3* specific CRISPR-Cas9 cassette and 3′ chunk donor DNA. Primers used to amplify the CRISPR-Cas9 cassette and encode *URA3* or *LEU2* specific guide RNAs, with guide RNA sequences in lower case (URA3 crRNA F: agcttggcagcaacaggactGTTTTAGAGCTAGAAATAGCAAGTTA.

URA3 crRNA R: agtcctgttgctgccaagctGATCATTTATCTTTCACTGCG, LEU2 crRNA F: ggcaacaaacccaaggaaccGTTTTAGAGCTAGAAATAGCAAGTTAAA, LEU2 crRNA R: acggttccttgggtttgttgccGATCATTTATCTTTCACTGCGGA). A duplication between the *ECM22* and *HAP1* genes on chromosome XII was discovered after sequencing of strain 55. This discrepancy was repaired by inserting a *URA3* marker cassette immediately after the stop codon of *EMC22*, growing without *URA3* selection overnight in YPD medium, plating for single colonies, and replica plating onto YNB glucose medium with 5FOA to select for colonies that had randomly looped out the chromosome XII duplication. Colonies were screened for loss of the *URA3* marker using PCR, and for single copies of the *SYM1* and *EST1* genes using RT-qPCR on genomic DNA with the *ALG9* gene used for normalization as previously described.[23] Removal of this duplication was subsequently confirmed using whole-genome sequencing, and had no effect on strain fitness. Fully synthetic versions of synXIV that were whole genome sequenced are described in Table S5.

MRPL19-GFP fusion protein design

File S5 contains annotated GenBank files of the plasmids and genes for the *MRPL19* protein internally tagged with super-folder Green Fluorescent Protein (sfGFP)[47] into its coding sequence with and without the loxPsym site in the 3′ UTR, and a cytosol-localized GFP control,[24] respectively. We inserted an in-frame *sfGFP* sequence inside the coding sequence of *MRPL19* (between position 282 and 283) because this gene encodes a predicted mitochondrial N-terminal peptide targeting signal[37] and a 3′ UTR mRNA signal that mediates mRNA localization to mitochondria-bound polysomes involved in mitochondria protein import.[26] The mitochondrial N-terminal peptide targeting signal was identified using MitoFates[37] and by generating and analyzing a 3D protein model of Mrpl19p using SWISS-MODEL,[48] which also revealed an N-terminal β-hairpin motif predicted to target proteins to mitochondria.[49] These mitochondrial targeting signals would have been disrupted by placing the sfGFP at either the N- or C-terminus of Mrpl19p. To promote proper folding of this fusion protein, we flanked the sfGFP with flexible linkers (L)[50] halfway through the *MRPL19* ORF. The resulting fusion protein had the following design: *MRPL1994-L-sfGFP-L-95MRPL19*. The native promoter and terminator regions were maintained, except for the version containing a loxPsym site 3 bp after the stop codon. These two cassettes were synthesized by Genscript Inc. and cloned onto pRS416 vectors using XhoI and NotI.

TAR1 expression construct cloning

The *TAR1* gene and its native promoter and terminator were synthesized as an IDT gBlock and cloned onto the pRS413 vector using BamHI and NotI restriction sites. The annotated vector map is included as File S6.

NOG2-GFP expression construct design and cloning

Expression constructs for *NOG2-GFP* fusion genes were synthesized by Genewiz and cloned onto pRS416 using. The native *NOG2* promoter and terminator were used, and two versions were made, with and without the *snr191* encoding *NOG2* intron sequence. Annotated GenBank files of these two plasmids are included in File S7.

tRNA-array design and cloning

As per Sc2.0 design principles, all tRNA genes are to be relocated.[4] To complement their loss from SynXIV, the synthetic ~9kb ChrXIV tRNA array was designed to house all 14 tRNA genes relocated from the wild-type chromosome XVI of *S. cerevisiae*. Each tRNA gene was assigned 500 bp 5’ and 40 bp 3’ flanking sequences recovered from the yeasts *Ashbya gossypii* or *Eremothecium cymbalariae* to reduce homology to the host genome. tRNA flanking sequence assignment was automated using Python

programming scripts based on an algorithm that matched tRNA genes to their flanking sequences preferentially by anticodon, and additionally altered unwanted artefacts (such as transcriptional gene starts) from the 5' flanking sequence. Furthermore, rox recombination sites were designed to be placed at 5' and 3' intervals and all tRNA introns were removed. Following synthesis (Wuxi Qinglan Biotech Co. Ltd (Yixing City, China)), the ChrXIV tRNA array was clone into a pRS413 centromeric vector with NotI restriction sites introduced for subsequent removal (File S8). There are no single-copy or otherwise essential tRNAs in this array.

Confocal microscopy

BY4741 strains transformed with *MRPL19-sfGFP-loxP-pRS416*, *MRPL19-sfGFP-Native 3′ UTR-pRS416,* or cytosol localized GFP expression (*pPDR12-GFP-pRS416*[24]) were pre-cultured twice in minimal medium without uracil before being inoculated at an A_{600nm} of 0.4 in fresh medium. Cells were treated with 100 nM Mitotracker Red FM (ThermoFisher M22425) for 3-4 h with shaking at 30°C. Cells were kept on ice prior to microscopic examination. Visualization of GFP and Mitotracker Red FM signals was performed using an Olympus FV 1000 confocal laser-scanning microscope. Microscopy images were analyzed using ImageJ (https://imagej.nih.gov/ij/index.html). Images shown are representative of cells in independent biological triplicate populations.

Diploid formation

Strains of opposite mating type and with complementary auxotrophies were grown overnight separately in 5 mL of selective SD media. 500 μL of each culture was used to inoculate the same non-selective 5 mL of SC medium, which was incubated overnight at 30°C without shaking. The overnight culture was washed twice in sterile MilliQ water before being plated on solid medium selective for the respective auxotrophies in each strain, such that only diploids would form colonies. Putative diploid colonies were checked using 'mating type' primers (key resources table) to verify the presence of both 'a' and 'alpha' alleles at the *MAT* locus, indicating the formation of a diploid.

Sporulation, random spore isolation, and random spore screening

To initiate sporulation, diploid colonies were grown overnight in 5 mL of selective liquid SD medium, washed once with sterile MilliQ water, and plated on 10 g/L potassium acetate medium. Plates were incubated in the dark at room temperature for 4-7 d. Once asci were visible under light microscopy, as many cells as possible were scraped from the potassium acetate plate and resuspended in 500 μL of sterile MilliQ water with 10 units of zymolyase and 20 μL of beta-mercaptoethanol. This solution was incubated at 37°C for 3-4 h before being transferred to a 250 mL flask containing 20 mL of 425-600 μm glass beads (Merck G9268) and 30 mL of sterile MilliQ water. Flasks were incubated at 30°C overnight with 200 rpm shaking. The liquid fraction was recovered, washed once in sterile MilliQ, and a dilution series down to 10^{-3} plated on YPD with incubation at 30°C for 1-2 d. Colonies were replica plated onto SD plates selective for each of the auxotrophic markers present in the haploid parent strains, and any colonies found not growing on each plate type were selected for PCR-tag analysis using the 2nd tag of each of the 22 synXIV megachunks. Colonies with synthetic PCR-tag amplification and without wild-type PCR-tag amplification were deemed likely to contain the corresponding megachunk, and further screened using all PCR-tags for the relevant megachunks.

RNA extraction

1.5 mL samples of mid-exponential phase cultures (A_{600} of 0.5 – 2.5) were pelleted by spinning at 12,000 x g for 2 min and removing the supernatant. Pellets were resuspended in 1 mL of RNAlater (ThermoFisher Scientific catalog number AM7020) and stored at -20°C. RNA was extracted after pelleting cells and removing RNAlater solution using the Zymo Research YeaStar RNA extraction kit (catalog number R1002) according to the user manual. Co-purified DNA was removed from RNA extracts using TURBO™ DNase (ThermoFisher Scientific catalog number AM2238) according to the user manual.

RT-qPCR

100 – 1000 ng of purified RNA was used for reverse transcription using an 18 nucleotide poly-T primer and SuperScript™ III Reverse Transcriptase (ThermoFisher Scientific 18080093) according to the user manual. A no-enzyme control was included for each RNA sample and subsequently used for qPCR to verify that no genomic DNA was contributing to cDNA concentration estimates. Reverse transcribed samples were diluted 1:100 in MilliQ water prior to qPCR analysis. Relative expression was performed using the modified-Livak method (amplification efficiency measured for each primer-pair and not assumed to be log2) with *ALG9* as a housekeeping gene,[51] as previously described.[23]

Whole-genome sequencing

A yeast genomic DNA extraction kit (ThermoFisher catalog number 78870) was used to isolate DNA according to the manufacturer's instructions. Sequencing and library preparation were carried out by Macrogen Inc. using a True-Seq Nano kit with 470 bp inserts, and paired-end Illumina HiSeq 2500 sequencing, or by the Ramaciotti Centre for Genomics using Nextera XT library preparation and 2x 150 bp paired end sequencing using a NextSeq500 (Sequencing of samples from the adaptive laboratory evolution experiment). Reads were analyzed using Geneious Pro v10.2.2 software.[52] Paired-end reads were mapped to an edited version of the S288C reference genome where native chromosome XIV was replaced with synthetic chromosome XIV (File S9). The Geneious alignment algorithm was used to map reads to the reference genome using default settings. Analysis of the resultant assembly was completed

visually by assessing read coverage, and read disagreement with the reference sequence. The raw reads were of high-quality (Q30 > 91 %, Q20 > 95 %), and were therefore not trimmed prior to assembly. Average read depth of 190 was typically achieved from the Macrogen sequencing, while 50-fold coverage was used for the samples sequenced at the Ramaciotti Centre for Genomics. Single Nucleotide Polymorphisms (SNPs) and their effect on ORF reading frames and codons were detected using the Geneious "Find Variations/SNPs" function with a variant p-value threshold of 10^{-6} and variant frequency of $\geq$ 50 %.

Nanopore sequencing

YP-glycerol evolved lineages had genomic DNA extracted as for Illumina sequencing. Barcoded nanopore sequencing libraries (SQK-LSK109) were prepared according to the manufacturer's instructions and sequenced on a single flowcell (FLO-MIN106) using a MinION sequencer. Basecalling of raw FAST5 files was performed using albacore (v2.3.1), with subsequent barcode demultiplexing using Porechop (v0.2.3). Demultiplexed reads in fastq format were assembled with Canu (v1.7.1), with initial assemblies for each strain polished using nanopolish (0.10.1). Assembled contigs were annotated using the Geneious Prime 'annotate from file' feature, with the S288C genome with SynXIV used as a reference.

The penultimate synXIV strain (74, Table S1) was whole-genome sequenced after DNA extraction as above. Genomic DNA (1-2 μg) was prepared for ligation sequencing (SQK-LSK109) with native barcoding (EXPNBD104 and EXPNBD114) as per the manufacturer instructions. Following preparation, 200-300 ng of DNA was loaded onto a MinION flowcell (FLO-MIN106) and basecalling was performed with Guppy v4.0.11 or v4.2.3 (Oxford Nanopore Technologies).

Flow cytometry

GFP was measured using exponentially growing cultures at an A_{600} of 0.5 using a Becton Dickinson Accuri C6 flow cytometer. GFP fluorescence was measured using a 488 nm laser and a 533/30 emission filter. Mean GFP values were divided by the mean autofluorescence of an empty vector control strain.

Polyploid strain construction

Polyploid strains were constructed through sequential rounds of synthetic mating type switching and strain mating. In short, *his3* and *leu2* auxotrophies were complemented in independent synthetic yLM896 and BY4742 strains. This was achieved by PCR amplification of the relevant gene loci from extracted prototrophic S288c genomic DNA using the sets his3up-F/R and leu2up-F/R primers (key resources table), and subsequent transformation into the respective strains to effectively restore the function of each respective auxotrophic loci. The relevant genotypes of the auxotrophic complementation strains are given in Table S4.

The WS and WW diploid strains were generated by co-inoculation of 1 mL of an exponential BY4741(k) culture (diluted to an A_{600} of 0.5) and 1 mL of an exponentially grown yLM896 or BY4742 strain, into 5 mL of YPD media and grown for 16 h at 30°C. The cultures were streaked out on SC –Met medium supplemented with 200 μg Geneticin to isolate single colonies. Diploid colonies were confirmed by the presence of both mating type loci via PCR amplification using the *MAT**a***, *MATα* and MATlocus primers (key resources table).

Diploid strains with heterozygous *MAT* loci are unable to mate spontaneously. To facilitate intermediate triploid strain generation, chemically synthesized *MAT**a*** DNA was transformed into the WS and WW strains. This allowed the generation of a small number of homozygous *MAT**a*** diploids within the population. After the heat shock step, transformed WS cells were co-inoculated with BY4742(L) cells and statically grown overnight in 5 mL fresh YPD medium at ambient temperature. The cell pellet was washed with sterile water, and the cell suspension was diluted and spread out to isolate single colonies on SC –Met – Leu agar plates. Putative triploid colonies resulting from mating were confirmed by the presence of both mating type loci via PCR amplification and named WWS. The same procedure was followed to generate the WWW and WSS strains, by mating the WW x BY4742(L) and WS x yLM896(L) strains respectively. The triploid genomic nature of each strain was verified through propidium iodide staining of nucleic acids and flow cytometry analysis.

The WWW, WWS, and WSS strains displaying a triploid genomic profile were selected for a subsequent round of strain transformation and mating as described above. The triploid strains had a *MATα*/*MAT**a***/*MAT**a*** active mating locus genotype, allowing the conversion of a small number of cells within the population to homozygous *MAT**a*** strains. After transformation, cell pellets were washed and combined with either yLM896(H) or BY4742(H) to allow mating during the stationary overnight incubation. Putative tetraploid colonies were selected on SC -His -Leu agar plates. The tetraploid strains resulting from mating between WWW x BY4742(H), WWS x BY4742(H), WWS x yLM896(H) and WSS x yLM896(H) were selected based on their DNA content flow cytometry profiles and ability to grow on selective plates without leucine, histidine, methionine and with 200 μg/mL Geneticin (data not shown). The verified strains were designated WWWW, WWWS, WWSS and WSSS respectively (Table S4).

For subsequent SCRaMbLE and growth characterization, the four constructed tetraploid strains, and the haploid (W, S) and diploid (WW, WS) strains were transformed with pHK-Cre-EBDh,[36] containing the Cre-EBD fusion-protein expression cassette. Strains containing the pHK-Cre-EBDh were selected on YDP agar plates supplemented with 200 μg/mL hygromycin B (Invivogen, USA).

Ploidy determination

The relative cell DNA content determination protocol was adapted from Rosebrock.[53] Overnight cultures were inoculated into fresh growth media to an A_{600} of 0.2 and were grown to mid-exponential phase. Adequate cell culture was harvested to obtain 500 μL of

culture at 2x10^{7} cells/mL. The cell pellet was washed with ice-cold water, and then fixed in 500 μL of ice-cold 70 % EtOH and incubated for at least 16 h at -20°C. The pellet was resuspended in Tris/$MgCl_2$-buffer (50 mM Tris-HCl, pH 7.7, and 15 mM $MgCl_2$) supplemented with RNase A to achieve a final concentration of 1mg/mL and incubated at 37°C for 90 min with gentle shaking. The pellet was then resuspended in 100 μL of 0.05 mM propidium iodide (PI) in Tris/ $MgCl_2$-buffer and allowed to strain for 48 h at 4°C. The sample was then diluted and analyzed with a BD FACSAria using the 488nm laser for PI excitation and the PE filter to measure red light emission. PI stained BY4742 and BY4743 were used as haploid and diploid control samples, respectively. The BY4742 G1 cell cycle fluorescence peak was used as reference to estimate a haploid DNA complement, and its G2 peak as estimate for a diploid DNA complement. The BY4743 G2 peak was used as estimate for cells with a tetraploid DNA complement. Cells with a G1 peak that corresponded with the BY4743 G2 peak were considered to have a tetraploid DNA content, while cells with a G1 peak intensity in between the G1 and G2 peaks of the BY4743 strain were considered triploid.

SCRaMbLE-in

A gene cassette for the expression of *URA3* was synthesized by Genewiz with loxPsym sites flanking the cassette. Exponential cultures of the S and WS, containing the Cre-recombinase expression vector, pLM006[36] were prepared and ~ 1.5 x 10^{7} cells were combined with 1 μg of loxPSym-flanked *URA3* cassette DNA using the LiAc/PEG transformation method. After heat shock, cells were washed in SC -His media, and resuspended in either 5 mL of SC -His media or SC-His medium supplemented with estradiol to achieve a final concentration of 1 μM for Cre-recombinase induction. Cells were recovered in these media for 1 h and the cell pellet washed twice with sterile water. The pellet was resuspended in sterile water, half was plated on SC -URA agar media (to select URA3 transformants) and the rest on YPD agar (to assess population viability). These were incubated at 30°C for two days.

Exponential cultures of the tetraploid strains WWWW, WWWS, WWSS and WSSS, containing the Cre-recombinase expression vector, pHK-Cre-EBDh plasmid were prepared and ~ 1.5 x 10^{7} cells were combined with 20μL of loxPSym-flanked *URA3* cassette PCR DNA using the LiAc/PEG transformation method. After heat shock, cells were washed in YPD media, and resuspended in either 5 mL of YPD + hygromycin media or YPD + hygromycin media supplemented with estradiol to achieve a final concentration of 1 μM to allow induction of the Cre-recombinase. Cells were recovered in these media for 1 h and the pellet washed twice with sterile water. The pellet was resuspended in sterile water, half was plated on SC -Ura agar media (to select URA3 transformants) and the rest on YPD agar (to assess population viability). These were incubated at 30°C for two days.

All transformations were done in triplicate. Colony forming units (cfu) were calculated from colony counts on two-day old plates and transformation efficiency calculated according to the online NEB calculator (www.nebiolabs.com.au) as cfu per μg of DNA used. Transformants per viable population was calculated by dividing the number of transformants (on -URA selection plates from induced cultures) by the number of colonies on non-selective YPD agar plated (from induced cultures).

Growth analysis of the tetraploid strains

To evaluate the effect of increased synthetic chromosomes on cell growth, the optical densities of W, S, WW, WS and the four tetraploid strains (containing the pHK-Cre-EBDh plasmid) were measured over time. Baffled flasks containing 15 mL fresh YPD containing 200 μg/mL hygromycin B were inoculated in triplicate to an A_{600} of 0.2 using stationary cultures of each respective strain. These cultures were incubated at 30°C with shaking at 250 rpm (Infors Multitron Pro). Optical density samples were taken every 2 h, after the first 8 h of growth. These represented the 'non-SCRaMbLE' reference samples. The viability of each strain after inducing SCRaMbLE through estradiol activated Cre-recombinase expression was also evaluated. In parallel to the growth analysis, W, S, WW, WS and the four tetraploid strains were grown as described above, except with the addition of estradiol (Sigma-Aldrich) at t = 0 to achieve a final concentration of 1 μM. These represented the 'SCRaMbLE' samples. Viability was reported as the difference between the estradiol untreated and treated samples of the same strain at each given time point.

QUANTIFICATION AND STATISTICAL ANALYSIS

Statistical analysis was performed using Prism 9 and Microsoft Excel software. All of the statistical details of experiments can be found in the figure legends and results, including the statistical tests used, exact value of n, what n represents, definition of centre, and dispersion and precision measures. Significance was defined using p-values of less than 0.05 with the tests indicated in the results section, no data or subjects were excluded.

Cell Genomics, Volume *3*

Supplemental information

Parallel laboratory evolution and rational debugging reveal genomic plasticity to *S. cerevisiae* synthetic chromosome XIV defects

Thomas C. Williams, Heinrich Kroukamp, Xin Xu, Elizabeth L.I. Wightman, Briardo Llorente, Anthony R. Borneman, Alexander C. Carpenter, Niel Van Wyk, Felix Meier, Thomas R.V. Collier, Monica I. Espinosa, Elizabeth L. Daniel, Roy S.K. Walker, Yizhi Cai, Helena K.M. Nevalainen, Natalie C. Curach, Ira W. Deveson, Timothy R. Mercer, Daniel L. Johnson, Leslie A. Mitchell, Joel S. Bader, Giovanni Stracquadanio, Jef D. Boeke, Hugh D. Goold, Isak S. Pretorius, and Ian T. Paulsen

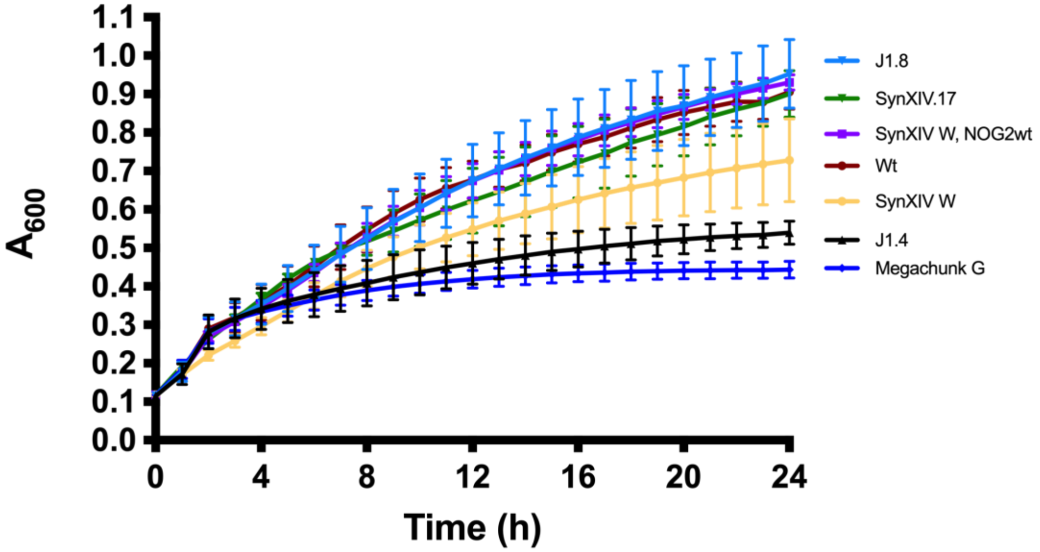

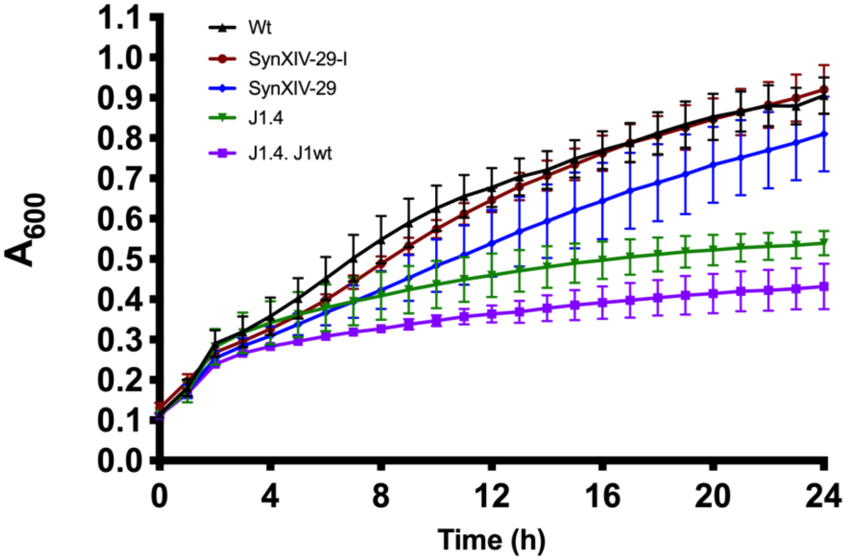

Figure S1. Fitness testing of major intermediate and defective strains made during synXIV construction

(**A**) Strains J1.8 (strain 40, Table S1), SynXIV.17 (strain 55, Table S1), SynXIV W, NOG2wt (strain 63, Table S1), Wt (strain 1 Table S1), SynXIV W (strain 57, Table S1), J1.4 (strain 39, Table S1), and megachunk G (strain 22, Table S1) were tested for fitness in liquid YPG medium. (**B**) Liquid medium fitness test of strains involved in the lead-up to the discovery of the *MRPL19* loxPsym defect (strains 37, 38, 39, 79 Table S1). Lines and error bars represent mean and ± 1 standard deviation of biological triplicate cultures. Related to Figure 1.

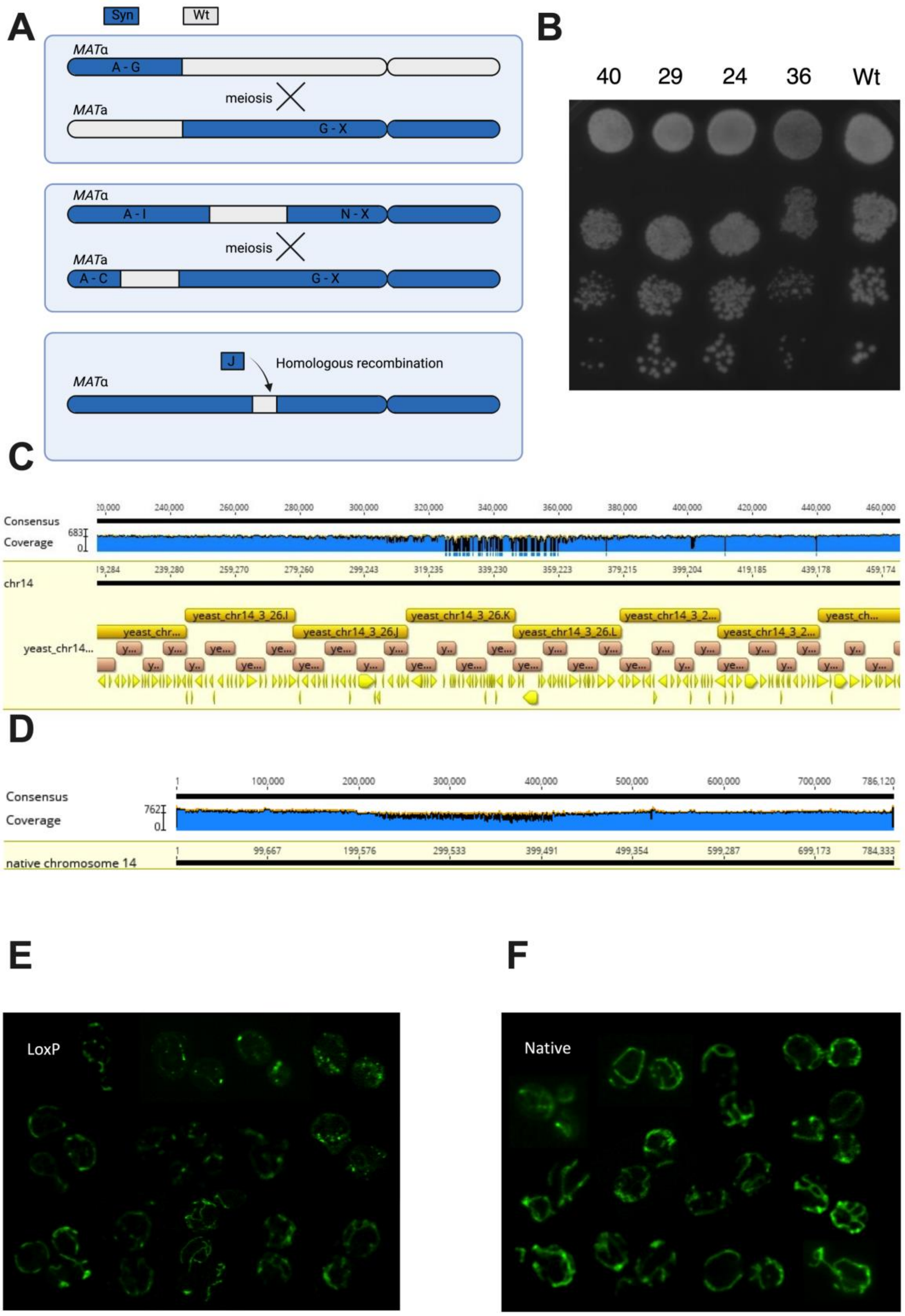

Figure S2. Pooled backcross sequencing and synthetic chromosome strain crossing reveals a fitness defect in megachunk J

(**A**) Megachunks A to X were integrated across two separate strains in parallel, with *URA3* and *MET17* markers integrated at the *YNL223W* and *YNR063W* wild-type loci of the synthetic A-G strain (strain 31, Table 3), respectively, prior to crossing with a synthetic G-X strain (strain 22, Table 3). Haploid strains resulting from the first cross (34 and 35, Table 3) were crossed together with screening for *LEU2*

(*YNL125C* locus) and *URA3* marker (*YNL269W* locus) loss from wild-type regions to give a fully synthetic version of chromosome XIV. (**B**) Haploid progeny of a meiotic cross between two partially synthetic versions (A-G and G-X) of synXIV (colonies 40, 29, 24, 36) were tested for fitness alongside a BY4741 control (Wt) on YPD agar at 30°C for 3 d. Colonies 40 and 36 contain fully synthetic chromosome XIV, while colonies 29 and 24 contain synthetic DNA in all megachunk regions except I-J and J respectively. (**C**) Pooled sequencing of 'fast' growing haploid progeny of a cross between synthetic chromosome XIV strain G-X and BY4742. Each set of reads were aligned to a reference S288c genome that also had a copy of the synthetic chromosome XIV sequence. (**D**) 'Slow' reads mapping to native chromosome XIV, with low-coverage regions equivalent to synXIV megachunk regions H to L. Dark yellow annotations correspond to megachunk regions while pink represent chunks, and yellow coding sequences. Average sequence coverage was 183 for the 'Fast' pool and 218 for the 'Slow'. Chromosome annotation and coverage graphs were generated using Geneious Pro Software. (**E**) Versions of the *MRPL19*-GFP fusion genes were designed with LoxP and (**F**) without LoxPsym motifs in the 3´ UTR GFP and expressed from pRS416 in the wild-type BY4741 strain. GFP fluorescence and distribution was visualised using an Olympus FV 1000 confocal laser-scanning microscope. Microscopy images were analysed using ImageJ (https://imagej.nih.gov/ij/index.html). Images shown are representative of cells in independent biological triplicate populations. Related to Figure 2.

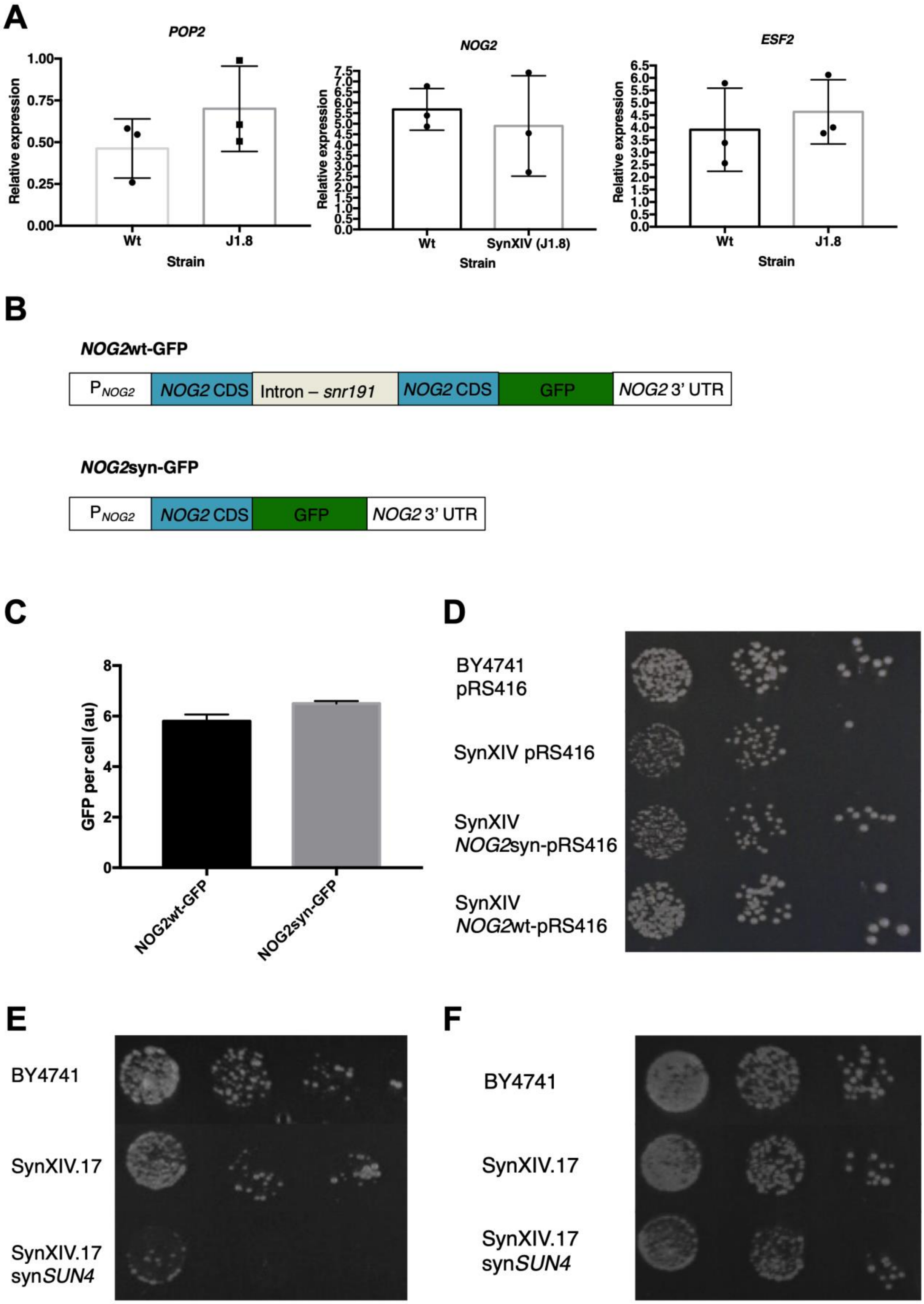

Figure S3. Expression of the *NOG2* and *SUN4* introns restores fitness

(**A**) mRNA levels of the three genes on chunk W1 were quantified using RT-qPCR. (**B**) Synthetic *NOG2-GFP* fusion expression constructs with and without the intron encoded *snr191* gene were designed and (**C**) tested for changes in protein expression in the wild-type (strain 1, Table 3). (**D**)

Expression of *NOG2-GFP* with its intron (strain 61, Table 3) but not without it (strain 62, Table 3) restores growth to wild-type levels in synXIV on YP-glycerol medium at 37°C. Images were taken after three days. RT-qPCR and GFP fluorescence levels are reported as the mean of triplicate cultures with error bars plus or minus one standard deviation. (**E**) Serial 10-fold dilutions of wild-type (BY4741), SynXIV (strain 63, Table 3), and SynXIV with the *SUN4* intron removed (strain 64, Table 3) on powdered yeast extract, and (F) on granulated yeast extract. Strains were plated on YP-glycerol (YPG) with non-granulated yeast extract (Merck 70161) at 37°C for 4 d prior to imaging. Related to Figure 4.

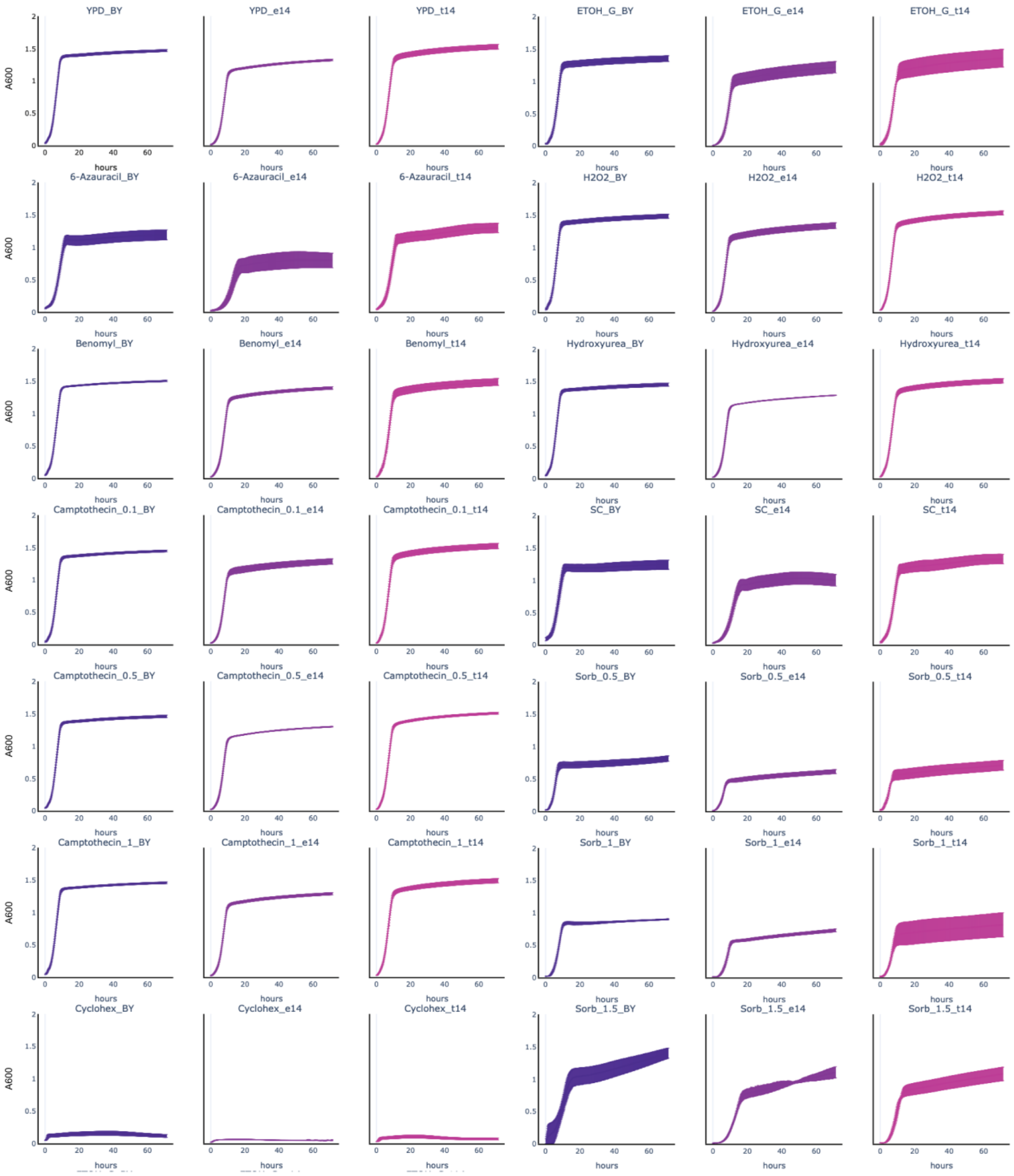

Figure S4. Fitness testing of final synXIV strain on different liquid media

Wild-type (BY4741 strain 75, Table S1), synXIV (strain 76, Table S1), and synXIV with tRNA genes reintroduced (strain 77, Table S1) were fitness tested in parallel on solid YPD, YPGE (1% Yeast extract 2% Peptone, 2% Glycerol, 2% Ethanol), or SC (Synthetic Complete) media with the indicated additives. YPD was used as the base medium for testing sorbitol, hydroxyurea, hydrogen peroxide, benomyl, 6-Azauracil, camptothecin, and cycloheximide. Lines and error bars represent mean and standard deviation from three biological replicates. Related to Figure 5.

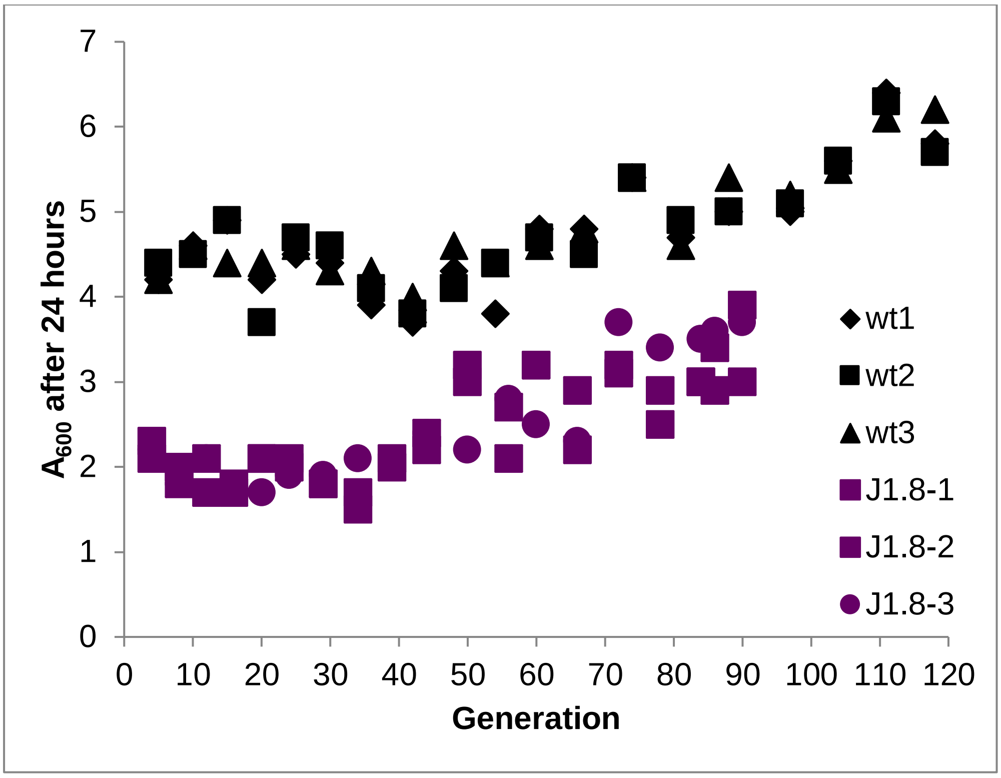

Figure S5. Fitness tracking of Adaptive Laboratory Evolution strains
A_{600} of three independent lineages for the BY4741 wild-type (strain 1 Table S1) and syn14 J1.8 strains (strain 40, Table S1) was measured every 24 h to track fitness and generation numbers in YP-glycerol medium. Related to Figure 6.

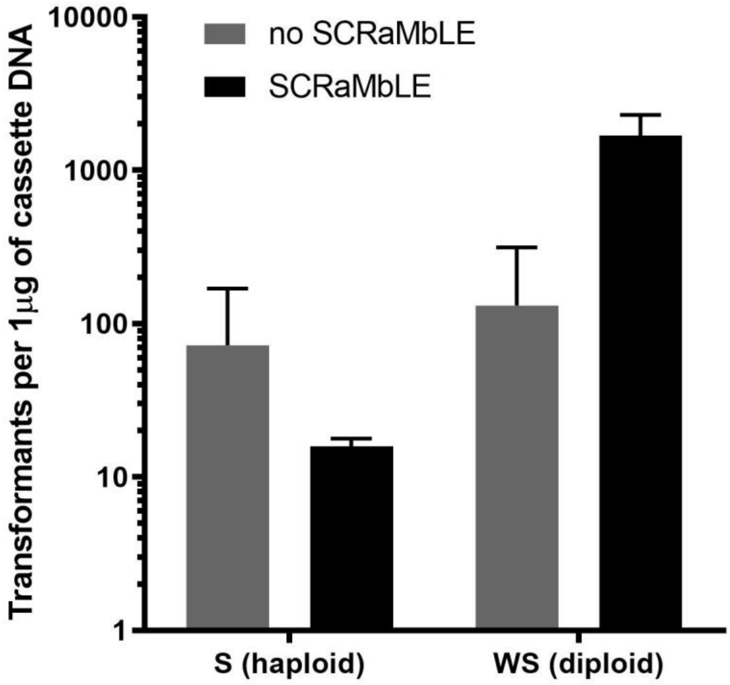

Figure S6. SCRaMbLE-mediated genome integration of a transformed *URA3* marker gene

Transformation efficiency in SCRaMbLE induced and uninduced haploid (strain 80, Table S4) and diploid strains (strain 86, Table S4). Both strains have the same number of synthetic chromosomes and, by extension, same number of available loxPsym integration sites. Related to Figure 7.

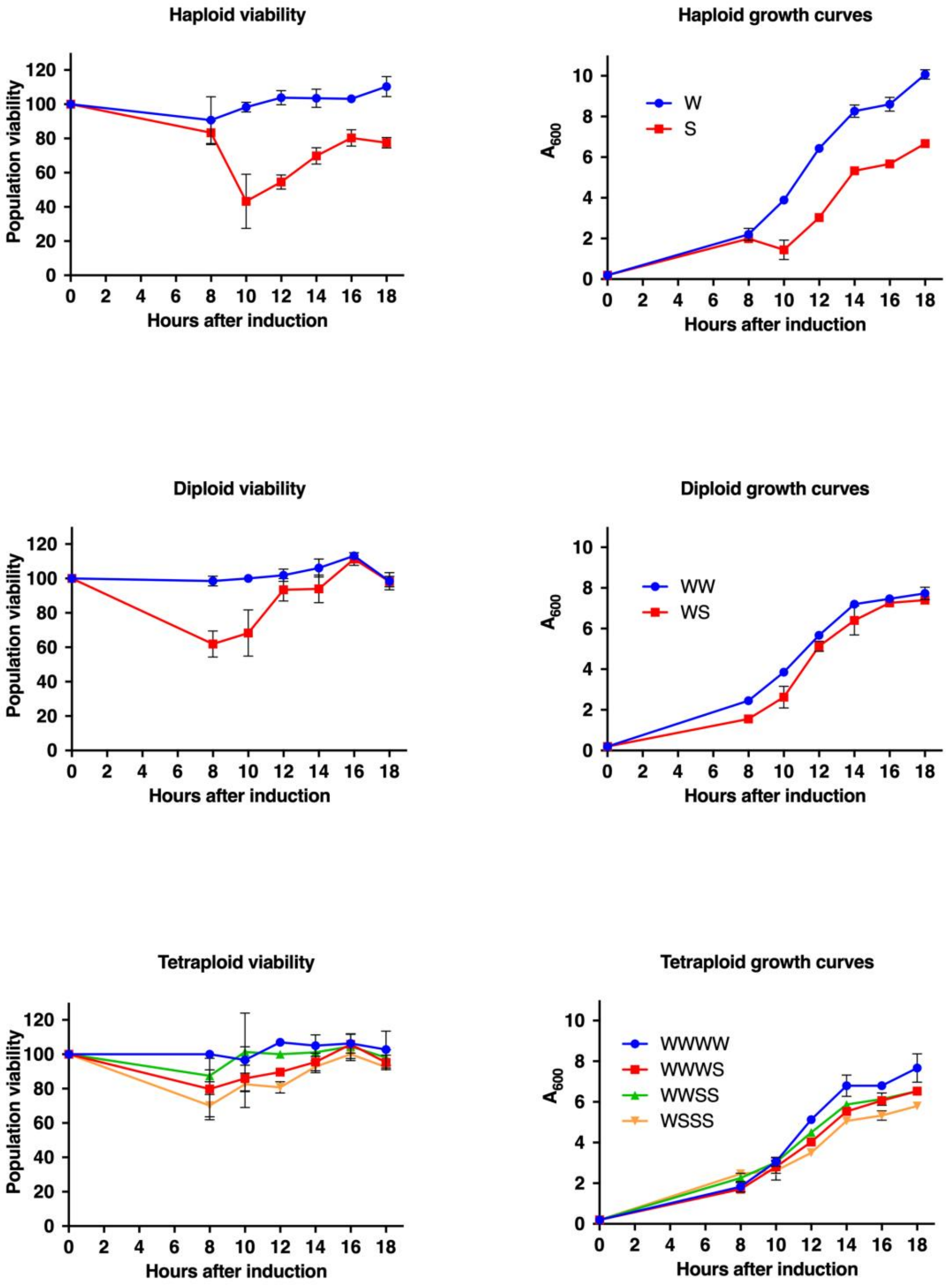

Figure S7. Growth and viability after SCRaMbLE of synthetic polyploid strains
The difference in cell density between SCRaMbLE'd and non-SCRaMbLE'd synthetic and wild-type cells of each strain were plotted for the haploid (A), diploid (C) and tetraploid (E) strains. The growth profiles of haploid (B), diploid (D) and tetraploid (F) strains in the presence of 1uM estradiol are shown. Data points and errors bars represent mean and standard deviation from three biological replicates. Related to Figure 7.

Table S2. Corrected sequence discrepancies in final synXIV strain, relates to STAR Methods.

Whole genome sequencing of synXIV strain revealed a number of missing Sc2.0 features and point mutations that deviated from the intended synXIV sequence. A subset of these were selected for repair according to their relative importance to the project, and ease of re-introduction. All missing TAA stop codons were repaired, as this modification will serve to free up the TAA codon to encode for non-natural amino acids in the future. All non-synonymous mutations in open reading frames were repaired to enable functional expression of the relevant proteins. Some missing loxPsym sites were corrected if they were nearby other features already being repaired, but were otherwise left as-is due to the fact that SCRaMbLE has a high degree of redundancy. PCR-tags are only used to verify the correct insertion of megachunks during the construction phase and were therefore left unaltered if missing, unless they were nearby another fix. Synonymous point mutations in open reading frames were also left unaltered.

Discrepancy Number	Discrepancy type	Original chunk/chromosome location	Gene(s)	Protein affect
1	T to C substitution	A3, 12735 bp	*YNL329C*	R276G
2	T insertion	A3, 13932 bp	*YNL328C*	Frame-shift
3	T to G substitution	A3, 13944 bp	*YNL328C*	N98H
4	C to T substitution	A3, 18438 bp	*YNL326C*	R302Q
5	G to T substitution	A3, 18512 bp	*YNL326C*	F277L
6	A to G substitution	A3, 18709 bp	*YNL326C*	Y212H
7	T insertion	A4, 19902 bp	*YNL325C*	Frame-shift
8	A to G substitution	A4, 22042 bp	*YNL325C*	synonymous
9	G to A transition	A4, 25505 bp	*intergenic*	-
10	G to A substitution	A4, 26370 bp	*YNL321W*	synonymous
11	Missing TAA stop codon	B4, 52811 bp	*YNL304W*	synonymous
12	Missing LoxP site	D4, 121257 bp	*YNL270C*	-
13	Missing TAA stop codon and LoxP site	E1, 125772 bp	*YNL268W*	synonymous

14	T to A substitution	E1, 125952 bp	*intergenic*	-
15	C to T substitution	E3, 110,759 bp	*YNL261W*	T377I
16	T to C substitution	K3, 336, 837 bp	*YNL149C*	None, TAA to TAG stop codon
17	Missing TAA stop codon	M3, 400807 bp	*YNL113C*	synonymous
18	Missing *Bsu36I* restriction site	M3-M4, 401355 bp	*YNL112W*	synonymous
19	T to C substitution	R1, 539,674 bp	*YNL037C*	T295A
20	Chunk V1 missing	V1, 2972 bp deletion beginning at 663,572 bp		YNR034 and YNR034W looped out between LoxP sites
21	Missing LoxP site	V4, 690721 bp	*YNR051C*	-
22	Missing TAA stop codon	V4, 690758 bp	*YNR051C*	synonymous
23	Missing PCR-tag	V4, 690776 bp	*YNR051C*	synonymous
24	Duplication on chromosome 12	*ECM22* to *HAP1*		

Table S3. Plasmids used in this study, relates to STAR Methods

**Name**	**Details**	**Origin**
pRS415	Yeast centromeric plasmid, *LEU2* marker	Euroscarf (Sikorski and Hieter 1989)
pRS416	Yeast centromeric plasmid, *URA3* marker	Euroscarf (Sikorski and Hieter 1989)
pRS413	Yeast centromeric plasmid, *HIS3* marker	Euroscarf (Sikorski and Hieter 1989)
TAR1-pRS413	*TAR1* expression from native promoter on the pRS416 plasmid	This study
pWAR1-crRNA-cas9-pRS423	*WAR1* promoter targeting guide RNA and Cas9 expression from the pRS423 plasmid. Template DNA for new crispr guide creation via PCR.	(Williams, Xu et al. 2017)
pPDR12-yEGFP-pRS416	*PDR12* promoter mediated expression of cytosol localised GFP. Positive 'Free GFP' control for confocal microscopy	(Williams, Xu et al. 2017)
MRPL19-GFP-MRPL19-LoxP-pRS416	MRPL19-GFP fusion protein expression construct with LoxPsym present in 3′ UTR	This study
MRPL19-GFP-MRPL19-Native-pRS416	*MRPL19*-GFP fusion protein expression construct with native 3′ UTR	This Study
NOG2wt-GFP-pRS416	Native *NOG2* promoter, intron, ORF, and terminator with in-frame ORF-yEGFP fusion	This study
NOG2syn-GFP-pRS416	Native *NOG2* promoter, no intron, ORF, and terminator with in-frame ORF-yEGFP fusion	This study
tRNA-pRS413		This study
pHK-Cre-EBDh	amp, CEN6/ARS4, SCW11p-CRE_EBD-ter, hphMX4 - For estradiol induced expression of Cre-recombinase. Contains a hygromycin resistance marker	This study
pLM006	amp, CEN6/ARS4, SCW11p-CRE_EBD-ter, HIS3 - For estradiol induced expression of Cre-recombinase. Contains a histidine auxotrophic marker	(Hochrein, Mitchell et al. 2018)

Table S4. Yeast strains used for polyploid construction, relates to Figure 7 and STAR Methods

Strain number	Strain name	Ploidy	Relevant genotype	Reference
79	BY4741(k)	n	*MAT*a, *his3Δ1, leu2Δ0, met15Δ0, ura3Δ0, mnn9::kanMX4*	Brachmann et al. (1998)
80	yLM896	n	*MAT*α, *leu2Δ0, MET15, his3Δ1, ura3Δ0;* synIII *HO::syn.SUP61;* SynIXL-synIXR; synVI *WT.PRE4*	Annaluru et al. (2014)
81	yLM896(L)	n	*MAT*α, *LEU2, MET15, his3Δ1, ura3Δ0;* synIII *HO::syn.SUP61;* SynIXL-synIXR; synVI *WT.PRE4*	This study
82	yLM896(H)	n	*MAT*α, *leu2Δ0, MET15, HIS3, ura3Δ0;* synIII *HO::syn.SUP61;* SynIXL-synIXR; synVI *WT.PRE4*	This study
83	BY4742(L)	n	*MAT*α, *his3Δ1, LEU2, lys2Δ0, ura3Δ0*	This study
84	BY4742(H)	n	*MAT*α, *HIS3, leu2Δ0, lys2Δ0, ura3Δ0*	This study
85	BY4742 x BY4741(k)<br><br>(WW)	2n	*MAT*α/a, *his3Δ1/his3Δ1, leu2Δ0/leu2Δ0, lys2Δ0/LYS2, MET15/met15, ura3Δ0/ura3Δ0, MNN9/mnn9::kanMX4*	This study
86	yLM896 x BY4741(k)<br><br>(WS)	2n	*MAT*α/a, *his3Δ1/his3Δ1, leu2Δ0/leu2Δ0, lys2Δ0/LYS2, MET15/met15, ura3Δ0/ura3Δ0, MNN9/mnn9::kanMX4;* chr III/synIII *HO::syn.SUP61;* chr IX/SynIXL-synIXR; chr VI/synVI *WT.PRE4*	This study
87	BY4742 x BY4741(k) x BY4742(L)<br><br>(WWW)	3n	*MAT*a/a/α, *his3Δ1/his3Δ1/his3Δ1, leu2Δ0/leu2Δ0/LEU2, lys2Δ0/LYS2/lys2Δ0, MET15/met15/MET15, ura3Δ0/ura3Δ0/ura3Δ0/, MNN9/mnn9::kanMX4/MNN9*	This study
88	BY4742 x BY4741(k) x yLM896(L)<br><br>(WWS)	3n	*MAT*a/a/α, *his3Δ1/his3Δ1/his3Δ1, leu2Δ0/leu2Δ0/LEU2, lys2Δ0/LYS2/lys2Δ0, MET15/met15/MET15, ura3Δ0/ura3Δ0/ura3Δ0/, MNN9/mnn9::kanMX4/MNN9;* chrIII/chrIII/synIII *HO::syn.SUP61;* chrIX/chrIX/SynIXL-synIXR; chrVI/chrVI/synVI *WT.PRE4*	This study
89	yLM896 x BY4741(k) x yLM896(L)	3n	*MAT*a/a/α, *his3Δ1/his3Δ1/his3Δ1, leu2Δ0/leu2Δ0/LEU2, lys2Δ0/LYS2/lys2Δ0,*	This study

	(WSS)		*MET15/met15/MET15, ura3Δ0/ura3Δ0/ura3Δ0/, MNN9/mnn9::kanMX4/MNN9;* chr III/synIII *HO::syn.SUP61//*synIII *HO::syn.SUP61;* chr IX/SynIXL-synIXR/SynIXL-synIXR; chr VI/synVI *WT.PRE4/*synVI *WT.PRE4*	
90	BY4742 x BY4741(k) x BY4742(L) x BY4742(H) (WWWW)	4n	*MAT*a/a/a/α, *his3Δ1/his3Δ1/his3Δ1/HIS3, leu2Δ0/leu2Δ0/LEU2/leu2Δ0, lys2Δ0/LYS2/lys2Δ0/lys2Δ0, MET15/met15/MET15/MET15, ura3Δ0/ura3Δ0/ura3Δ0/ura3Δ0, MNN9/mnn9::kanMX4/MNN9/MNN9*	This study
91	BY4742 x BY4741(k) x BY4742(L) x yLM896(H)<br><br>(WWWS)	4n	*MAT*a/a/a/α, *his3Δ1/his3Δ1/his3Δ1/HIS3, leu2Δ0/leu2Δ0/LEU2/leu2Δ0, lys2Δ0/LYS2/lys2Δ0/LYS2, MET15/met15/MET15/MET15, ura3Δ0/ura3Δ0/ura3Δ0/ura3Δ0, MNN9/mnn9::kanMX4/MNN9/MNN9;* chrIII/chrIII/chrIII/synIII *HO::syn.SUP61;* chrIX/chrIX/chrIX/SynIXL-synIXR; chrVI/chrVI/chrVI/synVI *WT.PRE4*	This study
92	yLM896 x BY4741(k) x yLM896(L) x BY4742(H)<br><br>(WWSS)	4n	*MAT*a/a/a/α, *his3Δ1/his3Δ1/his3Δ1/HIS3, leu2Δ0/leu2Δ0/LEU2/leu2Δ0, lys2Δ0/LYS2/lys2Δ0/lys2Δ0, MET15/met15/MET15/MET15, ura3Δ0/ura3Δ0/ura3Δ0/ura3Δ0, MNN9/mnn9::kanMX4/MNN9/MNN9; chrIII/*chr III/synIII *HO::syn.SUP61//*synIII *HO::syn.SUP61;* chrIX/SynIXL-synIXR/SynIXL-synIXR/chrIX; chrVI/synVI *WT.PRE4/*synVI *WT.PRE4/*chrVI	This study
93	yLM896 x BY4741(k) x yLM896(L) x yLM896(H)<br><br>(WSSS)	4n	*MAT*a/a/a/α, *his3Δ1/his3Δ1/his3Δ1/HIS3, leu2Δ0/leu2Δ0/LEU2/leu2Δ0, lys2Δ0/LYS2/lys2Δ0/lys2Δ0, MET15/met15/MET15/MET15, ura3Δ0/ura3Δ0/ura3Δ0/ura3Δ0, MNN9/mnn9::kanMX4/MNN9/MNN9; chrIII/*synIII *HO::syn.SUP61/*synIII *HO::syn.SUP61//*synIII *HO::syn.SUP61;* chrIX/SynIXL-synIXR/SynIXL-synIXR/ SynIXL-synIXR/ chrVI/synVI *WT.PRE4/*synVI *WT.PRE4/*synVI *WT.PRE4*	This study

Table S5. Strain version table, relates to STAR Methods

Version name	Strain number	Comment	Details
yeast_chr14_0_00	NA	Wild-type chromosome 14 sequence	GenBank: BK006947.3
yeast_chr14_3_26	NA	Original design sequence	Final design by BioStudio
yeast_chr14_9_01	SynXIV 29 I J1.4, Strain 39, Table S1	synXIV Draft strain, with 5 TAG stop codons, 10 loxPsym sites missing, 23 wild-type, 4 point mutations causing amino acid changes, *YNL066W* intron present.	**Remaining TAG stop codons**: 125772 A->G, 336549 T->C, 400292 T->C, 400807 A->G, 690758 T->C. **Missing loxPsym sites**: 120565-120598, 125051-125084, 250019-250052, 337131-337164, 374188-374221, 438943-438976, 603336-603369, 690721-690754, 746919-746952, 747387-747420. **Wild-type PCRTags**: 108692-109106 YNL273W_525, 131105-131132 YNL264C_193,174923-174950 YNL243W_1380, 175389-175416 YNL243W_2607, 180372-180399 YNL242W_3522, 249297-249324 YNL201C_2362, 250194-250221 YNL200C_139, 250428-250455 YNL200C_373, 281076-281103 YNL183C_1189, 281403-281430 YNL183C_1516, 337177-337204 YNL148C_10, 337465-337492 YNL148C_298, 341073-341100 YNL144C_1021, 410750-410777 YNL106C_3133, 489097-489118 YNL066W_673, 491634-491661 YNL065W_609, 491925-491952 YNL065W_900, 495487-495514 YNL063W_360, 595657-595684 YNL005C_388, 658820-658847 YNR031C_4591, 689809-689836 YNR051C_19, 725470-725497 YNR065C_2761, 747793-747820 YNR073C_370, 748138-748165 YNR073C_715. **Point mutations that cause amino acid changes**: 12071 G->A, 140826 C->T, 396611 T->A, 450624 T->C.
yeast_chr14_9_02	SynXIV 29 I J1.8, Strain 40, Table S1	synXIV Draft strain, removed *MRPL19* LoxP site	**Remaining TAG stop codons**: 125772 A->G, 336549 T->C, 400292 T->C, 400807 A->G, 690758 T->C. **Missing loxPsym sites**: 120565-120598, 125051-125084, 250019-250052, 337131-337164, 374188-374221, 438943-438976, 603336-603369, 690721-690754, 746919-746952, 747387-747420, 278873-278906. **Wild-type PCRTags**: 108692-109106 YNL273W_525, 131105-131132 YNL264C_193,174923-174950 YNL243W_1380, 175389-175416 YNL243W_2607, 180372-180399 YNL242W_3522, 249297-249324 YNL201C_2362, 250194-250221 YNL200C_139, 250428-250455 YNL200C_373, 281076-281103 YNL183C_1189, 281403-281430 YNL183C_1516, 337177-337204 YNL148C_10, 337465-337492

			YNL148C_298, 341073-341100 YNL144C_1021, 410750-410777 YNL106C_3133, 489097-489118 YNL066W_673, 491634-491661 YNL065W_609, 491925-491952 YNL065W_900, 495487-495514 YNL063W_360, 595657-595684 YNL005C_388, 658820-658847 YNR031C_4591, 689809-689836 YNR051C_19, 725470-725497 YNR065C_2761, 747793-747820 YNR073C_370, 748138-748165 YNR073C_715. **Point mutations that cause amino acid changes**: 12071 G->A, 140826 C->T, 396611 T->A, 450624 T->C.
yeast_chr14_9_03	SynXIV.17c NOG2 wt, A2-A3, V4, V1, R1, *YNL114W*, Chr12, K3, *YNL116W*^{L697I}, E3. Strain 77, Table S1	SynXIV draft strain. 3 TAG stop codons changed to TAA, 4 LoxPsym sites added, 4 non-synonymous point mutations corrected, 3 PCR-tags added, and 18 PCR tags removed during reconstruction and repair. *NOG2* intron re-inserted,	**Remaining TAG stop codons**: 400292 T->C. **Missing loxPsym sites**: 125051-125084, 250019-250052, 278873-278906, 337131-337164, 374188-374221, 438943-438976, 603336-603369, 715110-715143. **Wild-type PCRTags**: 108692-109106 YNL273W_525,116368-116395 YNL271C_1528, 124292-124319 YNL268W_390, 124640-124667 YNL268W_738, 124700-124727 YNL268W_798, 124901-124928 YNL268W_999, 126914-126941 YNL267W_684, 127169-127196 YNL267W_939, 128195-128222 YNL267W_1965, 128627-128654 YNL267W_2397, 129047-129074 YNL267W_2817, 129242-129269 YNL267W_3012, 131105-131132 YNL264C_193,174923-174950 YNL243W_1380, 175389-175416 YNL243W_2607, 180372-180399 YNL242W_3522, 249297-249324 YNL201C_2362, 250194-250221 YNL200C_139, 250428-250455 YNL200C_373, 337177-337204 YNL148C_10, 337465-337492 YNL148C_298, 341073-341100 YNL144C_1021, 410750-410777 YNL106C_3133, 489097-489118 YNL066W_673, 491634-491661 YNL065W_609, 491925-491952 YNL065W_900, 495487-495514 YNL063W_360, 595657-595684 YNL005C_388, 658820-658847 YNR031C_4591, 691283-691310 YNR051C_484, 691337-691364 YNR051C_538, 691670-691697 YNR051C_871, 696935-696962 YNR054C_58, 697238-697265 YNR054C_361, 715318-715345 YNR061C_172, 715507-715534 YNR061C_361. **Point mutations that cause amino acid changes**: 450624 T->C.
yeast_chr14_9_04	SynXIV.17c NOG2 wt, A2-A3, V4, V1, R1, *YNL114W*, Chr12, K3, *YNL116W*^{L697I},	SynXIV final strain. Non-synonymous point mutation in *IDH1* reverted to wild-type sequence	**Remaining TAG stop codons**: 400292 T->C. **Missing loxPsym sites**: 125051-125084, 250019-250052, 278873-278906, 337131-337164, 374188-374221, 438943-438976, 603336-603369, 715110-715143. **Wild-type PCRTags**: 108692-109106 YNL273W_525,116368-116395 YNL271C_1528, 124292-124319 YNL268W_390, 124640-124667 YNL268W_738, 124700-124727 YNL268W_798, 124901-124928 YNL268W_999, 126914-126941 YNL267W_684, 127169-127196

	E3, R1. Strain 78, Table S1		YNL267W_939, 128195-128222 YNL267W_1965, 128627-128654 YNL267W_2397, 129047-129074 YNL267W_2817, 129242-129269 YNL267W_3012, 131105-131132 YNL264C_193,174923-174950 YNL243W_1380, 175389-175416 YNL243W_2607, 180372-180399 YNL242W_3522, 249297-249324 YNL201C_2362, 250194-250221 YNL200C_139, 250428-250455 YNL200C_373, , 337177-337204 YNL148C_10, 337465-337492 YNL148C_298, 341073-341100 YNL144C_1021, 410750-410777 YNL106C_3133, 489097-489118 YNL066W_673, 491634-491661 YNL065W_609, 491925-491952 YNL065W_900, 495487-495514 YNL063W_360, 595657-595684 YNL005C_388, 658820-658847 YNR031C_4591, 691283-691310 YNR051C_484, 691337-691364 YNR051C_538, 691670-691697 YNR051C_871, 696935-696962 YNR054C_58, 697238-697265 YNR054C_361, 715318-715345 YNR061C_172, 715507-715534 YNR061C_361.
